# A Randomized, Double Blind, Placebo-Controlled Trial of Pioglitazone in Combination with Riluzole in Amyotrophic Lateral Sclerosis

**DOI:** 10.1371/journal.pone.0037885

**Published:** 2012-06-08

**Authors:** Luc Dupuis, Reinhard Dengler, Michael T. Heneka, Thomas Meyer, Stephan Zierz, Jan Kassubek, Wilhelm Fischer, Franziska Steiner, Eva Lindauer, Markus Otto, Jens Dreyhaupt, Torsten Grehl, Andreas Hermann, Andrea S. Winkler, Ulrich Bogdahn, Reiner Benecke, Bertold Schrank, Carsten Wessig, Julian Grosskreutz, Albert C. Ludolph, the GERP ALS Study Group

**Affiliations:** 1 Department of Neurology, University of Ulm, Ulm, Germany; 2 U692 - Laboratoire de Signalisations Moléculaires et Neurodégénérescence, INSERM, Strasbourg, France; 3 UMRS692, Faculté de Médecine, Université de Strasbourg, Strasbourg, France; 4 Department of Neurology, Hannover Medical School, Hannover, Germany; 5 Department of Neurology, University of Bonn, Bonn, Germany; 6 Department of Neurology, Charité University Hospital, Berlin, Germany; 7 Department of Neurology, University of Halle-Wittenberg, Halle, Germany; 8 Institute of Epidemiology and Medical Biometry, University of Ulm, Ulm, Germany; 9 Department of Neurology, Ruhr-Universität, Bochum, Germany; 10 Department of Neurology, Dresden University of Technology, Dresden, Germany; 11 Department of Neurology, Technical University, Munich, Germany; 12 Department of Neurology, University of Regensburg, Regensburg, Germany; 13 Neurology clinic, University of Rostock, Rostock, Germany; 14 Deutsche Klinik für Diagnostik, Wiesbaden, Germany; 15 Department of Neurology, University of Würzburg, Würzburg, Germany; 16 Department of Neurology, University Hospital, Jena, Germany; University G. D’Annunzio, Italy

## Abstract

**Background:**

Pioglitazone, an oral anti-diabetic that stimulates the PPAR-gamma transcription factor, increased survival of mice with amyotrophic lateral sclerosis (ALS).

**Methods/Principal Findings:**

We performed a phase II, double blind, multicentre, placebo controlled trial of pioglitazone in ALS patients under riluzole. 219 patients were randomly assigned to receive 45 mg/day of pioglitazone or placebo (one: one allocation ratio). The primary endpoint was survival. Secondary endpoints included incidence of non-invasive ventilation and tracheotomy, and slopes of ALS-FRS, slow vital capacity, and quality of life as assessed using EUROQoL EQ-5D. The study was conducted under a two-stage group sequential test, allowing to stop for futility or superiority after interim analysis. Shortly after interim analysis, 30 patients under pioglitazone and 24 patients under placebo had died. The trial was stopped for futility; the hazard ratio for primary endpoint was 1.21 (95% CI: 0.71–2.07, p = 0.48). Secondary endpoints were not modified by pioglitazone treatment. Pioglitazone was well tolerated.

**Conclusion/Significance:**

Pioglitazone has no beneficial effects on the survival of ALS patients as add-on therapy to riluzole.

**Trial Registration:**

Clinicaltrials.gov NCT00690118.

## Introduction

Amyotrophic lateral sclerosis (ALS) is a lethal neurological disease with limited therapeutic options. Only riluzole demonstrated efficacy in prolonging survival of ALS patients [1]. Inflammatory mediators are heavily produced in the CNS of ALS patients, and decreasing inflammation is protective in mouse models of ALS [2].

Pioglitazone is an oral anti-diabetic that stimulates the transcriptional activity of peroxisome proliferation activated receptors (PPARs), most notably of the PPAR subtype. Pioglitazone possesses anti-inflammatory properties that might be beneficial in ALS patients [3]. Interestingly, three independent groups reported beneficial effects of pioglitazone in ALS mouse models associated with decreased levels of inflammatory mediators [4], [5], [6]. Pioglitazone displays pleiotropic effects on energy metabolism through insulin sensitization, decreased glycaemia, and decreased circulating levels of liver enzymes and has been shown to be safe even in non-diabetic patients [7], [8], [9], [10]. A widely documented side effect of pioglitazone is a robust (3–5 kg) weight gain dependent upon neuronal, presumably hypothalamic, PPAR-γ receptors [7], [8], [9], [10], [11], [12]. These metabolic effects, and especially weight gain, might be clinically relevant for ALS since rescuing energy deficit in animal models alleviates neurodegeneration [13]. Furthermore, moderate obesity [14] and hyperlipemia [15], [16] have been associated with improved survival in ALS, suggesting that increased weight gain might be protective.

In all, pioglitazone represented a candidate drug able to act through multiple protective mechanisms. Here, we sought to test whether pioglitazone is beneficial in ALS by performing a phase II, multicentre, stratified, parallel-group, placebo-controlled trial of pioglitazone in ALS to assess the potential efficacy of pioglitazone as an add-on therapy to riluzole on survival as a primary endpoint. Incidence of non-invasive ventilation (NIV) and tracheotomy, and slopes of revised ALS functional rating scale (ALS-FRS-R), slow vital capacity (SVC), and quality of life (using EUROQoL EQ-5D; http: www.euroqol.org) were secondary endpoints.

## Methods

This phase II clinical trial was designed as a multicentre, stratified, parallel-group, placebo-controlled trial of pioglitazone in patients with ALS as an add-on therapy to riluzole. The trial protocol can be accessed at http://www.clinicaltrials.gov (NCT00690118). This protocol and supporting CONSORT checklist are available as supporting information; see Checklist S1 and Protocol S1.

### Participants

Patients with possible, probable (clinically or laboratory-supported) or definite ALS according to the revised version of the El Escorial World Federation of Neurology criteria were considered for enrolment into the study. Included patients displayed onset of progressive weakness within 36 months prior to study and had a disease duration of more than six months and less than three years (inclusive) with disease onset defined as date of first muscle weakness, excluding fasciculation and cramps. They reached a best-sitting SVC between 50% and 95% of predicted normal. They were capable of thoroughly understanding the information provided and giving full informed consent. Included women of childbearing age were non-lactating, and surgically sterile, or used a highly effective method of birth control, and had a negative pregnancy test. All included patients had been treated with 100 mg riluzole daily for at least three months prior to inclusion.

Exclusion criteria were: Participation in another clinical study within the preceding 12 weeks; tracheotomy or assisted ventilation during the preceding three months; gastrostomy; any medical condition known to have an association with motor neuron dysfunction which might confound the diagnosis of ALS; presence of any life-threatening disease or impairment likely to interfere with functional assessment; confirmed hepatic insufficiency or abnormal liver function (ASAT and/or ALAT >1.5 upper limit of normal); renal insufficiency (serum creatinine >2.26 mg/dL); evidence of major psychiatric disorder or clinically evident dementia; known hypersensitivity to the study drugs; patient likely to be not cooperative, not comply with the trial requirements (as assessed by the investigator), or unable to be reached in emergency; use of other antidiabetic drugs; heart failure, or history of heart failure (NYHA I to IV); history of macular oedema; treatment with thiazolidinediones within three months prior to screening; known or suspected history of alcohol and/or drug abuse; treatment with gemfibrozil within three months prior to screening.

### Ethics

All patients gave written informed consent. The ethics committee of the University of Ulm approved the study protocol.

### Interventions

The two treatment groups were 100 mg riluzole plus 45 mg pioglitazone (pioglitazone group), and 100 mg riluzole plus placebo (placebo group).

After inclusion, patients underwent a four-week screening phase, and a treatment phase (18 months). The dosage was increased stepwise: 15 mg once daily (od) during the first two weeks, 30 mg od during week three and four and 45 mg od from week five. Clinical and physical examinations, blood sampling, and drug compliance were recorded at on-site visits (1, 2, 6, 12 and 18 months after baseline visit). Body weight, and functional status (including NIV and ALS-FRS-R) were also recorded at 9 and 15 months after baseline visit through telephone contacts. The investigator observed patients for adverse events (AEs), and instructed patients to report any events. A period of 14 days for follow-up of AEs after the patient had routinely or prematurely terminated the study was performed additionally.

### Objectives

We sought to test whether pioglitazone 45 mg daily is beneficial in ALS patients under riluzole.

### Outcomes

The primary endpoint was survival time, *i.e*. time between inclusion in the study and death or last patient contact. Secondary efficacy outcomes were incidence of tracheotomy, and of NIV during study period, ALSFRS-R, and its changes, Quality of life using the EUROQoL EQ-5D and its changes.

### Sample Size

Sample size was calculated according to a previously published clinical trial in ALS [17]. The study was conducted using a two-stage group sequential test design within the Δ-class of critical values [18] with possible sample size adaptation after the planned interim analysis. A p-value of 0.5 was assumed as stopping for futility bound in interim analysis. For sample size calculation the following assumptions were made: One-sided alpha = 0.025 and 18-month survival rates of 60% in the placebo group and 78% in the pioglitazone group (hazard ratio = 0.486) [17], the statistical power is 80% if the log-rank test is performed after observation of 31 deaths at the first stage and after 62 deaths at the second stage according to the formula of Schoenfeld (using ADDPLAN® version 3.1.2, ADDPLAN GmbH, Germany).

Pioglitazone was originally developed as oral antidiabetic drug and clinical trials showed that pioglitazone was well tolerated. At present, it has been prescribed to over 3.5 million patients. Thus, the use of a one-sided statistical test for primary efficacy analysis was considered as acceptable. Assuming an accrual time of six months, a treatment period of 18 months, and a constant accrual rate, a total of 176 subjects (88 subjects per group) was expected to yield the necessary number of deaths. Under these assumptions, the time points of analyses were expected to be 12.3 month’s observation time (interim analysis) and 24 months observation time (final analysis). Considering a dropout rate of 10%, inclusion of 196 patients was planned.

### Randomization- sequence Generation, Allocation and Implementation

At the randomisation visit, each patient eligible for study participation was randomised to one of the two treatment groups, and received the next consecutive randomisation/patient number according to her/his stratum from a block of randomisation numbers per site. The code was broken only for interim analysis, and at the end of study.

Haupt Pharma Brackenheim GmbH (Brackenheim, Germany) generated the randomisation list. Block-randomisation with a block size of four was performed. A pseudo-random number generator ensured that the resulting treatment sequence was reproducible. Patients were assigned to their stratum according to the location of the earliest ALS symptom. Patients with simultaneous spinal and bulbar onset were defined as bulbar. Cervical and respiratory onsets were stratified to the spinal-onset stratum.

### Blinding

The trial was double-blinded. Study medication was packed and blinded by Haupt Pharma Brackenheim.

### Statistical Methods

The study population was analysed according to the intention-to-treat principle. All randomized patients who received at least one intake of study medication were analysed for efficacy and safety.

To investigate efficacy, the unstratified log rank test was used to compare groups. The statistical hypotheses in terms of the hazard ratio were the following:

H0: λ2/λ1> = 1 (i.e. identical or poorer survival in the pioglitazone group).

H1: λ2/λ1<1 (i.e. better survival in the pioglitazone group),

Where λ2/λ1 is the hazard ratio, λ1 denotes the hazard in the placebo group; λ1 denotes the hazard in the pioglitazone group.

The overall type-I-error rate was set at α = 0.025 (one-sided).

For comparison of baseline characteristics, we performed group comparisons using Mann-Whitney test for continous items and χ^2^ test for binary outcomes. Binary secondary outcomes (incidence of tracheotomy or NIV) were calculated both as total number and as percentage including the 95% confidence interval and group comparisons were performed using the χ^2^ test. For continuous secondary outcomes (ALSFRS-R, EUROQoL EQ-5D) group comparisons were performed using mixed effects regression model analysis. All secondary endpoints were analysed descriptively. All statistical tests performed were two-sided at a significance level of 5%. All results from analysis of the secondary endpoints were regarded as hypothesis generating only, and not as proof of efficacy.

An independent statistician performed the interim analysis at July 17th 2009 at the Institute of Epidemiology and Medical Biometrics at the University of Ulm. The interim analysis was performed with data from all available patients on June 10th, 2009 (197 patients, pioglitazone n = 99, placebo n = 98). Interim analysis was initially planned after observation of 31 deaths. However, because of the unexpected low number of deaths from start of trial until March 2009, the protocol was amended to perform the interim analysis either after observation of a total of 31 deaths or in June 2009, depending on what condition happens first. Statistical analyses were performed at the Institute of Epidemiology and Medical Biometrics at the University of Ulm using the statistical software package SAS Version 9.2 under Windows.

## Results

### Participant Flow and Recruitment

**Figure 1:**
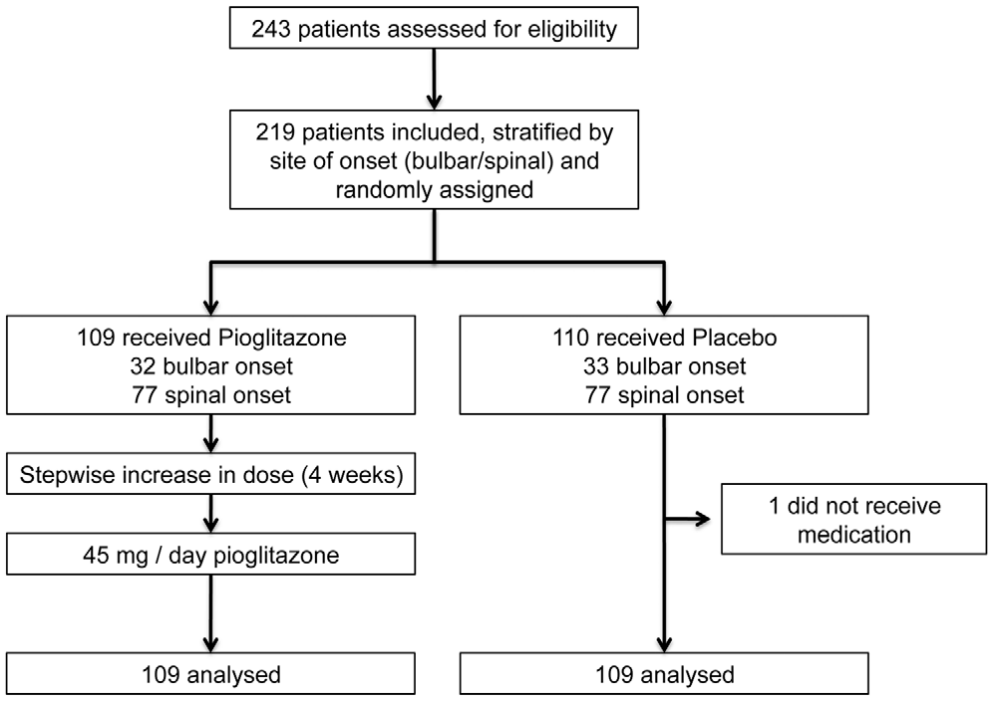
Study flow chart. The figure presents the numbers of participants who were randomly assigned, received pioglitazone or placebo, and were analysed for the primary outcome.

Between the 29^th^ of May 2008 and the 14th of August 2009, 219 ALS patients were enrolled, and randomly allocated to either placebo (n = 110; bulbar: n = 33, spinal: n = 77), or pioglitazone (n = 109; bulbar: n = 32, spinal: n = 77) treatment after stratification based on site of onset (bulbar or spinal). One patient in the placebo group did not take any dose of study medication and was therefore excluded from the study. Figure 1 shows the study flow chart.

### Baseline Data

Baseline characteristics of both groups were similar (Table 1). In particular, both groups of patients showed similar sex ratio, age and functional status as measured by revised ALS functional rating scale (ALSFRS-R) and slow vital capacity (SVC, a measure of respiratory function). Since energy metabolism is a modifyer of ALS disease [19] and pioglitazone interferes with energy metabolism, we also monitored BMI, glycaemia, circulating liver enzymes (ASAT and ALAT) and found these different parameters similar at inclusion between both groups (Table 1). Blood pressure showed a trend to be higher in the pioglitazone group.

**Table 1:** Baseline characteristics of ALS patients.

	Pioglitazone (n = 109)	Placebo (n = 109)	*p* value
Sex ratio (M/F)	63/46	71/38	0.33
Age at enrolment (years)	58.9 (10.6)	59.0 (10.4)	0.94
Site of onset (bulbar/spinal)	31/77	33/76	0.88
ALSFRS-R	37.5 (6.0)	37.0 (5.6)	0.51
SVC (%)	76.2 (16.6)	73.5 (15.4)	0.21
Body weight (kg)	72.8 (12.7)	74.1 (13.1)	0.46
BMI (kg/m2)	24.7 (3.8)	24.8 (3.7)	0.84
Glycaemia (mg/dL)	95.9 (13.3)	94.3 (14.8)	0.40
ASAT (U/L)	35.8 (12.1)	32.7 (11.3)	0.06
ALAT (U/L)	40.1 (17.9)	37.8 (21.3)	0.39
Blood pressure (systolic/diastolic, mm Hg)	133.2 (16.9)/83.0 (9.5)	138.5 (19.7)/87.0 (11.3)	0.07/0.02
Time from symptom onset to diagnosis (months)	9.6 (5.6)	8.7 (5.7)	0.25
Time from symptom onset to baseline screening (months)	18.9 (8.6)	18.6 (9.0)	0.81

Values are indicated as mean (SD).

### Primary Outcome

**Figure 2:**
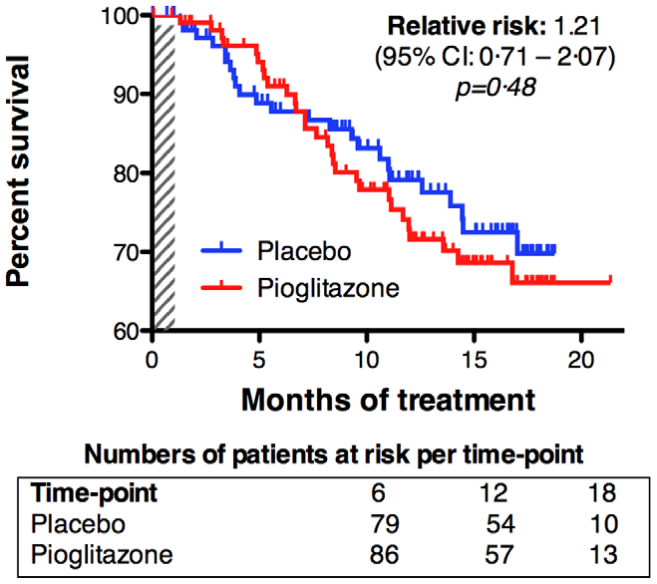
Survival upon pioglitazone. Kaplan-Meier plot for survival, the primary endpoint of the trial. Placebo-treated patients are in blue and pioglitazone treated patients are in red. Ticks represent censored patients. The shaded box indicates the first month of treatment during which a stepwise increase in pioglitazone dosage was performed. Numbers below the X axis indicate the number of patients still alive (“at risk”, *i.e.* living and not censored) at entry, 6, 12 and 18 months after randomization.

In interim analysis, which was performed about one year after start of recruiting patients, we observed death of 24 patients, 14 in the pioglitazone group and 10 in the placebo group. There was no difference between pioglitazone group and placebo group. Since the number of deaths was unexpectedly low, the data monitoring and safety committee (DMSC) requested continuation of the trial. Number of deaths and death rate were provided monthly and the DMSC recommended to stop the trial for futility on the 28^th^ of April 2010 due to the increased death rate in the pioglitazone group (pioglitazone, 30 deaths; placebo, 24 deaths). In final statistical analysis, the hazard ratio was 1.21 (95%CI: 0.71–2.07, *p* = 0.48). This means that the hazard for death was increased of 21% in the pioglitazone group and the difference between both groups was not significant. The comparison of survival curves for both treatment groups showed no difference in survival (Figure 2).

### Secondary Outcomes

**Figure 3:**
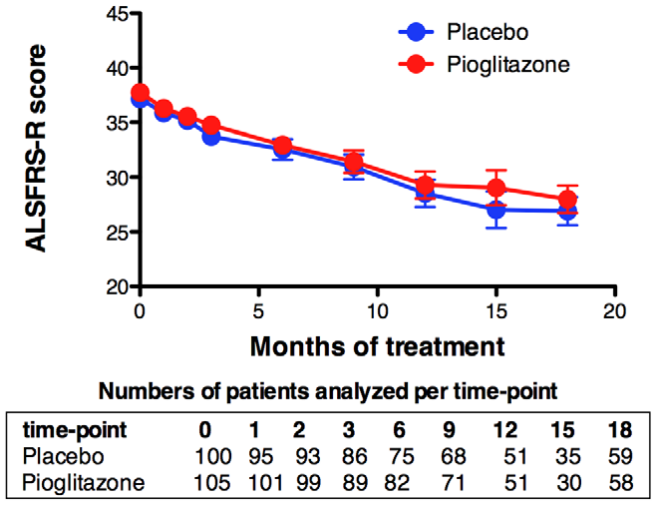
ALS-FRS upon pioglitazone. Total changes in ALS-FRS-R score after initiation of the treatment. Placebo-treated patients are in blue and pioglitazone treated patients are in red. The table below indicates the number of patients (n) per time point. Error bars are standard errors.

Secondary variables for efficacy were also non-significantly affected by pioglitazone treatment. ALS-FRS-R score and slope was not affected by pioglitazone (*p* = 0.66, Figure 3), and this was also the case for quality of life and slow vital capacity (not shown). The incidence of tracheotomy during the study period was unchanged by pioglitazone (pioglitazone: 6.4%, placebo: 4.6%, *p* = 0.54). So was the incidence of NIV (pioglitazone: 20.2%, placebo: 26.6%, *p* = 0.28).

### Adverse Events

Pioglitazone was well tolerated and most adverse events were due to ALS disease progression, *e.g.* dysphagia, dyspnoea or respiratory failure rather than to pioglitazone treatment, *e.g.* oedema (table 2).

**Table 2:** Adverse events.

Adverse event	Pioglitazone (n = 109)	Placebo (n = 109)	*p* value
Dysphagia	32 (29.4%)	34 (31.2%)	0.88
Dyspnoea	35 (32.1%)	31 (28.4%)	0.66
Respiratory failure	33 (30.3%)	31 (28.4%)	0.88
Oedema	26 (23.9%)	18 (16.5%)	0.24
Depression	14 (12.8%)	13 (11.9%)	1.00
Weight loss	18 (16.5%)	9 (8.3%)	0.10
Constipation	15 (13.8%)	11 (10.1%)	0.53
Fatigue	12 (11.0%)	14 (12.8%)	0.83
Nasopharyngitis	13 (11.9%)	12 (11.0%)	0.83
Fall	11 (10.1%)	11 (10.1%)	1.00
Back pain	9 (8.3%)	10 (9.2%)	1.00
Musculoskeletal pain	9 (8.3%)	7 (6.4%)	0.80
Headache	7 (6.4%)	7 (6.4%)	1.00
Muscle spasms	7 (6.4%)	7 (6.4%)	1.00
Salivary hyper secretion	6 (5.5%)	8 (7.3%)	0.78
Sleep disorder	6 (5.5%)	8 (7.3%)	0.78
Bronchitis	7 (6.4%)	5 (4.6%)	0.77
Dizziness	8 (7.3%)	4 (3.7%)	0.37
Pain in extremity	10 (9.2%)	2 (1.8%)	0.03
Pneumonia	7 (6.4%)	5 (4.6%)	0.77
Urinary tract infection	9 (8.3%)	3 (2.8%)	0.13
Nausea	6 (5.5%)	5 (4.6%)	1.00
Pain	6 (5.5%)	5 (4.6%)	1.00

## Discussion

In this randomized clinical trial, we could not show that pioglitazone is a valid therapeutic option for ALS patients under riluzole.

A number of clinical trials have been performed in ALS since the positive trial of riluzole (Table S1 and references therein) [1]. Except the recent small trial on dexpramipaxole [20], these trials were all negative. Most of them were based on preclinical results obtained in SOD1 mouse models that were either single studies in animals, that have not been reproduced by other laboratories (creatine [21], [22], celecoxib [21], [22], [23]), or controversial studies (lithium [24], [25], [26], IGF-1 [27], [28], [29]) (Table S1 and references therein). To our knowledge, only minocycline [30], [31], [32] and pioglitazone [5], [6] have been shown to display protection in mutant SOD1 mice by at least two independent laboratories and have been tested in clinical trials (this study and [33]). These disappointing results claim strongly for the generation of alternative models of ALS. Indeed, the recent identification of new genetic causes of ALS, in particular intronic expansions in the Chr9 ORF 72 gene that account for more than 30% of familial ALS, and a subset of sporadic ALS [34], [35], could provide unvaluable alternative preclinical models. Mutant SOD1 mice currently represent the only animal model that reliably reproduces the phenotype of ALS patients, but one should keep in mind that SOD1 mutations only represent 2% of total ALS cases and do not lead to TDP43/FUS/optineurin inclusions [36], [37], [38].

In the specific case of pioglitazone, three groups independently reported protection by pioglitazone in mutant SOD1 mice [4], [5], [6]. The causes of the discrepancy between our negative clinical trial result, and these promising preclinical trials are still elusive. First, preclinical trials might have been inadequately designed since they did not follow guidelines published after their completion [22], [39]. In particular these guidelines now indicate to use at least 12 animals per group and per gender in a fully blinded manner [39]. Both studies used 7–12 mice per group for survival analysis, with Schutz and collaborators [5] using only male mice while Kiaei and collaborators [6] did not specify gender. Blinding, mandatory in the new guidelines, was performed for one of the studies [5], but is not mentionned for the other one. Second, the animal model might be relevant for studying mechanisms, but of poor relevance for preclinical trials. Third, we cannot completely exclude that riluzole masks beneficial effects of pioglitazone, for instance through modification of its brain disposal. Indeed, riluzole interferes with P-glycoprotein efflux pomp and modifies brain disposal of minocycline [40]. Thus, despite pioglitazone is known to cross partially the blood brain barriers in mammals [41], it may not have reached the concerned brain regions in concentrations that would have allowed for neuroprotection due to interference with riluzole. Of note, a poor translation of the dosage is unlikely since doses used correspond to dosages recently described for their efficacy in non-diabetic patients. However, preclinical trials have been performed in mice before disease onset, and the time window of pioglitazone administration might well be inadequate. Indeed, PPARγ and inflammation-promoting transduction pathways such as NF-κB are antagonistic [42], [43]. Given the robust inflammatory reaction in the ventral horn of ALS patients [2] initiation of treatment after several months of disease progression might be too late. Our study illustrates the difficult translation from mouse models to human ALS, and strongly claims for the generation of new SOD1-independent animal models.

Summarizing, we could not find any protective effect of pioglitazone in ALS patients under riluzole. The cause for this failed clinical translation remains elusive.

## Supporting Information

Table S1 **Previous clinical trials in ALS since riluzole trials (1994–1996).**
(DOC)

Checklist S1 **CONSORT Checklist.**
(DOC)

Protocol S1 **Trial Protocol.**
(PDF)
