# Supplementary material for: A Randomized, Double Blind, Placebo-Controlled Trial of Pioglitazone in Combination with Riluzole in Amyotrophic Lateral Sclerosis
Source: PLoS One. 2012 Jun 8;7(6):e37885. doi: 10.1371/journal.pone.0037885 (PMC3371007; doi:10.1371/journal.pone.0037885)
Supplement: Table S1 — Previous clinical trials in ALS since riluzole trials (1994–1996). (DOC) [file pone.0037885.s001.doc]

Supplementary table 1: previous clinical trials in ALS since riluzole trials (1994-1996)

| **Drug** | **Study** | **Patients** | **Intervention** | **Add-on to riluzole** | **Design** | **Results** | **Preclinical trials in mSOD1 mice** |
| --- | --- | --- | --- | --- | --- | --- | --- |
| **Reproduced preclinical evidence** | | | | | | | |
| Minocycline | Gordon et al., 2007[1] | 412 patients | placebo or minocycline in escalating doses of up to 400 mg/day for 9 months | Possible | multicentre, randomised placebo-controlled phase III trial | Harmful effect | Three consistent studies showing benefit in mSOD1 mice |
| Pioglitazone | This study | 219 patients | Placebo or 45 mg Pioglitazone per day | Yes | double-blind, placebo-controlled, multicenter, randomized clinical trial | No efficacy, CNS resistance to drug | Two consistent studies showing survival benefit in mSOD1 mice |
| **Single study preclinical evidence** | | | | | | | |
| celecoxib | Cudkowicz, 2006[2] | 300 patients | celecoxib (800 mg/day) or placebo for 12 months | Possible | double-blind, placebo-controlled, clinical trial | No efficacy on muscle strength | One study, no replication |
| CoQ 10 | Kaufmann et al., 2009[3] | 185 patients in two stages | Placebo or 1800mg/day, or 2700 mg/day of CoQ10 during 9 months | Possible | adaptive, two-stage, bias-adjusted, randomized, placebo-controlled, double-blind trial | No benefit | One study, no replication |
| Creatine | Shefner, 2004[4] | 104 patients | Placebo or 5g creatine per day during 6 months | Possible | randomized,  double-blind, placebo-controlled trial | No efficacy on muscle strength | One study, no replication |
| Creatine | Groeneveld, 2003[5] | 175 patients | Placebo or 10g creatine per day during 16 months | Yes | Randomized sequential trial | No efficacy on survival | One study, no replication |
| Gabapentin | Miller et al., 2001[6] | 204 patients | oral gabapentin 3,600 mg or placebo daily for 9 months | No | double-blind, placebo-controlled, multicenter randomized | No efficacy on muscle strength | One study, no replication |
| Gabapentin | Miller et al., 1996[7] | 152 patients | Gabapentin (800 mg) or placebo tid | No | multicenter, double-blind, randomized, placebo-controlled trial | No effect on functional decline | One study, no replication |
| Tocopherol | Desnuelle et al., 2001[8] | 289 patients | alpha-tocopherol (500 mg daily) or placebo | Yes | double-blind, placebo-controlled, randomized | No efficacy on survival | One study, no replication |
| **Controversial preclinical evidence** | | | | | | | |
| Glatiramer acetate | Meininger et al., 2009[9] | 366 patients | placebo or 40 mg glatiramer acetate daily | Yes | double- blind, randomized, multicentre, placebo-controlled trial | No efficacy on ALS-FRS-R | Controversial |
| Lithium | Chio et al., 2010[10] | 171 patients | Two doses: subtherapeutic dose (pseudo-placebo, 0.2-0.4 mEq/L) and therapeutic dose (0.4-0.8 mEq/L) for 15 months | Possible | multicenter, single-blind, randomized, dose-finding trial | Reduced tolerability, No efficacy | Controversial |
| Lithium | Aggarwal et al., 2010[11] | 84 patients | Lithium carbonate (target concentration: 0.4-0.8mEq/L) or placebo. | Yes | multicenter, double-blind, randomized, placebo-controlled trial | No efficacy | Controversial |
| Lithium | Miller et al., 2011[12] | 356 patients | Lithium carbonate (300-450 mg/d, target concentration: 0.3-0.8mEq/L) or placebo. | Possible | multicenter, double-blind, randomized, placebo-controlled trial | No efficacy | Controversial |
| Subcutaneous IGF-1 | Sorenson et al., 2008[13] | 330 patients | 0.05 mg/kg body weight of human recombinant IGF-1 given subcutaneously twice daily or placebo for 2 years | Possible | Randomized, double-blind, placebo-controlled study | No benefit | Controversial, no study using this route of administration |
| Topiramate | Cudkowicz, 2003[14] | 296 patients | Placebo or topiramate (800mg/day) for 12 months | Possible | double-blind, placebo-controlled, multicenter randomized | Harmful effect on function, no effect on survival | No effect on survival |
| Valproic acid | Piepers et al, 2009[15] | 163 ALS patients | VPA 1,500mg or placebo daily for 16 months | Yes | Randomized sequential trial | No beneficial effect on survival or disease progression | Controversial |
| **No preclinical evidence** | | | | | | | |
| Talampanel | Pascuzzi et al., 2010[16] | 59 patients | 40 subjects receiving talampanel 50 mg p.o. t.i.d,  19 subjects receiving placebo for 9 months | Possible | double-blind, placebo-controlled, multicenter, randomized clinical trial | No efficacy in terms of isometric muscle arm strength | None |
| TCH346 | Miller et al., 2007[17] | 591 patients | placebo or one of four doses of TCH346 (1.0, 2.5, 7.5, or 15 mg/day) administered orally once daily for at least 24 weeks | Possible | Randomized, double-blind, placebo-controlled trial | No efficacy on ALS-FRS deterioration | None |
| pentoxyfilline | Meininger, 2006[18] | 400 patients | placebo or 1.2 g pentoxifylline daily during 18 months (547 days) | Yes | double-blind, randomized, placebo-controlled, multicenter trial | Harmful effect on survival, No effect on function | None |
| Indinavir | Scelsa et al., 2005[19] | 46 patients | 2400 mg / day indinavir per os or placebo | Possible | randomized, doubleblind,  placebo-controlled trial | No efficacy in ALS-FRS | None |
| Xaliproden | Meininger et al., 2004[20] | 867 patients | placebo, 1 mg or 2 mg xaliproden orally once daily as monotherapy | No | randomized, double-blind, placebo-controlled, multi-centre, multi-national studies | No effect on efficacy; trends towards improvement in some secondary outcomes measures. | None |
| Xaliproden | Meininger et al., 2004[20] | 1210 patients | placebo, 1 mg or 2 mg xaliproden orally once daily with addition of riluzole 50 mg bid background therapy | Yes | randomized, double-blind, placebo-controlled, multi-centre, multi-national studies | No effect on efficacy; trends towards improvement in some secondary outcomes measures. | None |
| rhCNTF | Miller et al., 1996[21] | 570 patients | 0.5, 2, or 5 micrograms/kg/day rhCNTF, or placebo, for 6 months | No | double-blind, placebo-controlled | Harmful at highest doses | None |
| BDNF | BDNF Study group, 1999[22] | 1135 patients | placebo, or 25 or 100 microg/kg BDNF for 9 months. | Possible | double-blind, placebo-controlled | No effect | None |

**Supplementary references**

1. Gordon PH, Moore DH, Miller RG, Florence JM, Verheijde JL, et al. (2007) Efficacy of minocycline in patients with amyotrophic lateral sclerosis: a phase III randomised trial. Lancet Neurol 6: 1045-1053.

2. Cudkowicz ME, Shefner JM, Schoenfeld DA, Zhang H, Andreasson KI, et al. (2006) Trial of celecoxib in amyotrophic lateral sclerosis. Ann Neurol 60: 22-31.

3. Kaufmann P, Thompson JL, Levy G, Buchsbaum R, Shefner J, et al. (2009) Phase II trial of CoQ10 for ALS finds insufficient evidence to justify phase III. Ann Neurol 66: 235-244.

4. Shefner JM, Cudkowicz ME, Schoenfeld D, Conrad T, Taft J, et al. (2004) A clinical trial of creatine in ALS. Neurology 63: 1656-1661.

5. Groeneveld GJ, Veldink JH, van der Tweel I, Kalmijn S, Beijer C, et al. (2003) A randomized sequential trial of creatine in amyotrophic lateral sclerosis. Ann Neurol 53: 437-445.

6. Miller RG, Moore DH, 2nd, Gelinas DF, Dronsky V, Mendoza M, et al. (2001) Phase III randomized trial of gabapentin in patients with amyotrophic lateral sclerosis. Neurology 56: 843-848.

7. Miller RG, Moore D, Young LA, Armon C, Barohn RJ, et al. (1996) Placebo-controlled trial of gabapentin in patients with amyotrophic lateral sclerosis. WALS Study Group. Western Amyotrophic Lateral Sclerosis Study Group. Neurology 47: 1383-1388.

8. Desnuelle C, Dib M, Garrel C, Favier A (2001) A double-blind, placebo-controlled randomized clinical trial of alpha-tocopherol (vitamin E) in the treatment of amyotrophic lateral sclerosis. ALS riluzole-tocopherol Study Group. Amyotroph Lateral Scler Other Motor Neuron Disord 2: 9-18.

9. Meininger V, Drory VE, Leigh PN, Ludolph A, Robberecht W, et al. (2009) Glatiramer acetate has no impact on disease progression in ALS at 40 mg/day: a double- blind, randomized, multicentre, placebo-controlled trial. Amyotroph Lateral Scler 10: 378-383.

10. Chio A, Borghero G, Calvo A, Capasso M, Caponnetto C, et al. (2010) Lithium carbonate in amyotrophic lateral sclerosis: lack of efficacy in a dose-finding trial. Neurology 75: 619-625.

11. Aggarwal SP, Zinman L, Simpson E, McKinley J, Jackson KE, et al. (2010) Safety and efficacy of lithium in combination with riluzole for treatment of amyotrophic lateral sclerosis: a randomised, double-blind, placebo-controlled trial. Lancet Neurol 9: 481-488.

12. Miller RG, Moore DH, Forshew DA, Katz JS, Barohn RJ, et al. (2011) Phase II screening trial of lithium carbonate in amyotrophic lateral sclerosis: examining a more efficient trial design. Neurology 77: 973-979.

13. Sorenson EJ, Windbank AJ, Mandrekar JN, Bamlet WR, Appel SH, et al. (2008) Subcutaneous IGF-1 is not beneficial in 2-year ALS trial. Neurology 71: 1770-1775.

14. Cudkowicz ME, Shefner JM, Schoenfeld DA, Brown RH, Jr., Johnson H, et al. (2003) A randomized, placebo-controlled trial of topiramate in amyotrophic lateral sclerosis. Neurology 61: 456-464.

15. Piepers S, Veldink JH, de Jong SW, van der Tweel I, van der Pol WL, et al. (2009) Randomized sequential trial of valproic acid in amyotrophic lateral sclerosis. Ann Neurol 66: 227-234.

16. Pascuzzi RM, Shefner J, Chappell AS, Bjerke JS, Tamura R, et al. (2010) A phase II trial of talampanel in subjects with amyotrophic lateral sclerosis. Amyotroph Lateral Scler 11: 266-271.

17. Miller R, Bradley W, Cudkowicz M, Hubble J, Meininger V, et al. (2007) Phase II/III randomized trial of TCH346 in patients with ALS. Neurology 69: 776-784.

18. Meininger V, Asselain B, Guillet P, Leigh PN, Ludolph A, et al. (2006) Pentoxifylline in ALS: a double-blind, randomized, multicenter, placebo-controlled trial. Neurology 66: 88-92.

19. Scelsa SN, MacGowan DJ, Mitsumoto H, Imperato T, LeValley AJ, et al. (2005) A pilot, double-blind, placebo-controlled trial of indinavir in patients with ALS. Neurology 64: 1298-1300.

20. Meininger V, Bensimon G, Bradley WR, Brooks B, Douillet P, et al. (2004) Efficacy and safety of xaliproden in amyotrophic lateral sclerosis: results of two phase III trials. Amyotroph Lateral Scler Other Motor Neuron Disord 5: 107-117.

21. Miller RG, Petajan JH, Bryan WW, Armon C, Barohn RJ, et al. (1996) A placebo-controlled trial of recombinant human ciliary neurotrophic (rhCNTF) factor in amyotrophic lateral sclerosis. rhCNTF ALS Study Group. Ann Neurol 39: 256-260.

22. (1999) A controlled trial of recombinant methionyl human BDNF in ALS: The BDNF Study Group (Phase III). Neurology 52: 1427-1433.
