# Supplementary material for: A Randomized, Double Blind, Placebo-Controlled Trial of Pioglitazone in Combination with Riluzole in Amyotrophic Lateral Sclerosis
Source: PLoS One. 2012 Jun 8;7(6):e37885. doi: 10.1371/journal.pone.0037885 (PMC3371007; doi:10.1371/journal.pone.0037885)
Supplement: Protocol S1 — Trial Protocol. (PDF) [file pone.0037885.s003.pdf]

CLINICAL TRIAL PROTOCOL

PROTOCOL NUMBER: GERP ALS

EUDRACT NUMBER: 2006-005410-13

EFFICACY, SAFETY AND TOLERABILITY STUDY OF 45 mg PIOGLITAZONE IN PATIENTS WITH AMYOTROPHIC LATERAL SCLEROSIS (ALS) RECEIVING STANDARD THERAPY (RILUZOLE)

Sponsor	University Hospital of Ulm<br>Prof. Reinhard Marre, MD<br>Principal Medical Director<br>Albert-Einstein-Allee 29<br>89081 Ulm / Germany
Coordinating investigator:	Prof. A. C. Ludolph, MD<br>Department of Neurology<br>University of Ulm<br>Oberer Eselsberg 45<br>D-89081 Ulm / Germany

Confidentiality

The information provided in this document is the property of Professor Dr. A.C. Ludolph. It is strictly confidential but is available for review to investigators, potential investigators, appropriate Ethics Committees or Investigational Review Boards and Competent Authorities. No disclosure is allowed without written authorization from A.C. Ludolph except to the extent necessary to obtain informed consent from potential study patients.

SIGNATURE PAGE

Prof. R. Marre, MD

Principal Medical Director

University of Ulm

Germany

(Sponsor)	(Signature)	(Date)

Prof. A. C. Ludolph, MD

Director Department of Neurology

University of Ulm

Germany

(Coordinating investigator)	(Signature)	(Date)

Werner Baurecht

acromion GmbH

Frechen / Germany

(Statistician)	(Signature)	(Date)

THE TRIAL HAS TO BE CONDUCTED ACCORDING TO THIS PROTOCOL, THE GOOD CLINICAL PRACTICE AND THE APPLICABLE REGULATORY REQUIREMENTS

CONTACT ADDRESSES AND RESPONSIBILITIES

Sponsor	University Hospital of Ulm<br>Prof. Reinhard Marre, MD<br>Principal Medical Director<br>Albert-Einstein-Allee 29<br>89081 Ulm / Germany<br>Phone: +49 (0)731 500-43001<br>Fax: +49 (0)731 500-43002
Legal Representative of the sponsor and Coordinating Investigator	Prof. Albert Christian Ludolph, MD<br>Department of Neurology<br>University of Ulm<br>Oberer Eselsberg 45<br>89081 Ulm / Germany<br>Phone: +49 (0)731 177 1200<br>Fax: +49 (0)731 177 1202
Safety Contact	Prof. Jan Kassubek, MD<br>Department of Neurology<br>University of Ulm<br>Oberer Eselsberg 45<br>89081 Ulm<br>Phone: +49 (0)731 177 1206<br>Fax: +49 (0)731 177 1202
Central Laboratory	Prof. Hayrettin Tumani, MD / Prof. Markus Otto, MD<br>Department of Neurology, Neurochemical Laboratory<br>University of Ulm<br>Oberer Eselsberg 45<br>89081 Ulm / Germany<br>Phone: +49 (0)731 177 1207<br>Fax: +49 (0)731 177 1519

CRO / Project Management	Ulrike Schomaker, MD<br>acromion GmbH<br>Europaallee 27-29<br>50226 Frechen<br>Phone: +49 (0)2234 203737 16<br>Fax: +49 (0)2234 203737 9<br>Email: ulrike.schomaker@acromion-gmbh.com
Statistical Evaluation	PD Rainer Muche, PhD<br>Department of Biometry and Medicinal Documentation<br>University of Ulm<br>Schwabstrasse 33<br>89075 Ulm / Germany<br>Phone: +49 (0)731 500 26903<br>Fax: +49 (0)731 500 26902
Statistical Planning	Werner Baurecht, Dipl. Stat.<br>acromion GmbH<br>Europaallee 27-29<br>50226 Frechen<br>Phone +49 (0)2234 203737 17<br>Fax +49 (0)2234 203737 9

TABLE OF CONTENTS

SIGNATURE PAGE 2

CONTACT ADDRESSES AND RESPONSIBILITIES 3

LIST OF ABBREVIATIONS 9

1 TRIAL SUMMARY 11

2 BACKGROUND AND RATIONALE 14

2.1 NAME AND DESCRIPTION OF THE INVESTIGATIONAL PRODUCT 14

2.2 FINDINGS FROM NON-CLINICAL AND CLINICAL STUDIES RELEVANT TO THE TRIAL 14

2.3 KNOWN AND POTENTIAL RISKS AND BENEFITS, IF ANY, TO HUMAN SUBJECTS 15

2.4 DESCRIPTION OF AND JUSTIFICATION FOR THE ROUTE OF ADMINISTRATION, DOSAGE, DOSAGE REGIMEN, AND TREATMENT PERIOD(S) 16

3 STUDY PURPOSE, TRIAL OBJECTIVES AND ENDPOINTS 16

4 SUBJECT SELECTION 17

4.1 TRIAL POPULATION 17

4.2 TRIAL ADMISSION 17

4.3 SELECTION CRITERIA 17

4.3.1 INCLUSION CRITERIA 17

4.3.2 EXCLUSION CRITERIA 18

4.4 PROTOCOL VIOLATIONS 18

5 TRIAL DESIGN 19

5.1 DESIGN OVERVIEW 19

5.2 TRIAL PROCEDURES 19

5.3 TRIAL VISITS SCHEDULE **21**

5.4 UNSCHEDULED VISITS **24**

5.5 VISITS FOLLOWING PREMATURE DISCONTINUATION OF STUDY TREATMENT **24**

5.6 SEQUENCE AND DURATION OF TRIAL PERIODS **24**

6 STUDY TREATMENTS **25**

6.1 TEST PREPARATIONS **25**

6.2 TREATMENT PACKAGING **25**

6.3 TREATMENT LABELLING **25**

6.4 STORAGE OF DRUG SUPPLIES **26**

6.5 TREATMENT ASSIGNMENT **27**

6.6 TREATMENT ADMINISTRATION **27**

6.7 OVERDOSE **27**

6.8 COMPLIANCE **28**

6.9 CONCOMITANT TREATMENT **28**

6.10 RANDOMISATION AND UNBLINDING PROCEDURES **28**

6.10.1 RANDOMISATION 28

6.10.2 BLINDING 29

6.10.3 EMERGENCY PROCEDURE FOR UNBLINDING 29

6.11 DRUG ACCOUNTABILITY **29**

7 ADVERSE EVENTS **30**

7.1 DEFINITIONS **30**

7.1.1 ADVERSE EVENT (AE) 30

7.1.2 SERIOUS ADVERSE EVENT (SAE) 30

7.1.3 NON SERIOUS ADVERSE EVENTS 31

7.1.4 SEVERITY (INTENSITY) AND OUTCOME 31

7.2 MONITORING AND RECORDING OF ADVERSE EVENTS **32**

7.3 ASSESSMENT OF ADVERSE EVENTS 33

7.3.1 EVALUATION CRITERIA 33

7.3.2 POINTS TO CONSIDER 34

7.4 DOCUMENTATION AND REPORTING OF SERIOUS ADVERSE EVENTS 35

7.4.1 REPORTING RESPONSIBILITY 35

7.4.2 REPORTING PROCEDURES 35

7.4.3 RESPONSIBLE NOMINATED SAFETY CONTACT 36

7.5 UNBLINDING OF TREATMENT / EMERGENCY IDENTIFICATION 36

7.6 PREMATURE DISCONTINUATION / PATIENT REPLACEMENT 36

7.6.1 INVESTIGATOR'S RESPONSIBILITY FOLLOWING PREMATURE DISCONTINUATION OF PATIENTS 37

7.6.2 STUDY TERMINATION 37

8 STATISTICS 37

8.1 SAMPLE SIZE AND POWER CONSIDERATIONS 37

8.2 STATISTICAL METHODS 38

8.2.1 POPULATIONS 38

8.2.2 BACKGROUND AND DEMOGRAPHIC CHARACTERISTICS 39

8.2.3 STUDY MEDICATION 39

8.2.4 CONCOMITANT THERAPY 39

8.2.5 EFFICACY EVALUATION 39

8.2.6 SAFETY EVALUATION 40

8.2.7 INTERIM ANALYSIS 41

8.2.8 OTHER EVALUATIONS 41

8.3 DATA HANDLING AND RECORD KEEPING 41

9 ETHICAL ASPECTS 42

9.1 INDEPENDENT ETHICS COMMITTEE APPROVAL 42

9.2 INFORMED CONSENT **42**

9.3 DATA CONFIDENTIALITY **43**

9.4 INSURANCE **43**

10 ADMINISTRATIVE PROCEDURES **43**

10.1 REGULATORY ASPECTS **43**

10.2 QUALITY ASSURANCE **44**

10.2.1 MONITORING 44

10.2.2 DATA MONITORING AND SAFETY COMMITTEE (DMSC) 44

10.2.3 SOURCE DATA DEFINITION AND VERIFICATION (SDV) 45

10.2.4 DATA RECORDING 45

10.2.5 RETENTION OF DOCUMENTS 45

10.2.6 AUDITING PROCEDURES 46

10.3 DISCLOSURE AND CONFIDENTIALITY **46**

10.3.1 CONFIDENTIALITY OF DATA 46

10.4 PROTOCOL AMENDMENTS **47**

10.5 CLINICAL TRIAL REPORT AND PUBLICATION **47**

11 REFERENCES **47**

12 APPENDICES **49**

LIST OF ABBREVIATIONS

AE	Adverse Event
ALAT	Alanine Aminotransferase
ALS	Amyotrophic Lateral Sclerosis
ALS FRS-R	ALS Functional Rating Scale, revised version
AMG	"Arzneimittelgesetz" *German Drug Law*
ASAT	Aspartate Aminotransferase
ATC	Anatomical therapeutic chemical classification
BfArM	„Bundesinstitut für Arzneimittel und Medizinprodukte" *German CA*
bid	Twice daily
CA	Competent Authority
CRF	Case Report Form
CRO	Contract Research Organization
DMSC	Data Monitoring and Safety Committee
Euroqol EQ-5D	Quality of Life Questionnaire
FVC	Forced Vital Capacity
GCP	Good Clinical Practice
GCP-V	"GCP-Verordnung" *German GCP Regulation*
GP	General Practitioner
Hb	Haemoglobin
HCG	Human Chorion Gonadotropin
Hct	Haematocrit
ICH	International Conference of Harmonisation
IEC	Independent Ethics Committee
IRB	Institutional Review Board
MCH	Mean Corpuscular Haemoglobin
MCHC	Mean Corpuscular Haemoglobin Concentration
MCV	Mean Corpuscular Volume
MedDRA	Medical Dictionary for Regulatory Activities
NIV	Non-invasive ventilation

od	Once daily
NYHA	New York Heart Association
RBC	Red Blood Cells
SAE	Serious Adverse Event
SC	Steering Committee
SOP	Standard Operating Procedures
WBC	White Blood Cells
WHO	World Health Organization

1 TRIAL SUMMARY

Title

Efficacy, safety, and tolerability study of 45 mg pioglitazone in patients with amyotrophic lateral sclerosis (ALS) receiving standard therapy (riluzole)

Primary objective

Efficacy of pioglitazone (45 mg/day) as add-on therapy to standard therapy with riluzole in patients with amyotrophic lateral sclerosis compared to placebo in terms of survival (mortality defined exclusively as death)

Secondary objectives

Secondary efficacy variables will include incidence of tracheotomy or non-invasive ventilation (NIV), ALS Functioning Rating Scale (ALSFRS-R), and measurement of quality of life using the Euroqol EQ-5D. Clinical tolerability and safety will be assessed by findings of physical examination, adverse events incidence, and clinical laboratory values.

Study design and overview

This is a prospective, multicentre, randomized, stratified, parallel-group, double-blind trial comparing placebo with 45 mg pioglitazone as add-on therapy to 100 mg riluzole in amyotrophic lateral sclerosis (ALS) in 220 enrolled patients. For entry, the El Escorial Criteria for the diagnosis of ALS will be used. The patients have to be stable on riluzole at least 3 months prior to randomization. After patients are screened for inclusion and exclusion criteria, they will be randomly assigned to treatment with either placebo or 45 mg pioglitazone according to their stratum (bulbar onset vs. spinal onset). The trial requires an initial screening visit (V0), one baseline visit (V1), 4 subsequent control visits (V2 – V5), and a final visit (V6).

Number of patients

The population consists of 220 enrolled ALS patients as defined by the El Escorial criteria, and modified by the Airlie House criteria (http://www.wfnals.org). 110 patients will be randomized to treatment with 45 mg pioglitazone and 110 patients to treatment with placebo, respectively.

Number of study centers

This is a multicentre study including approximately 16 sites in Germany. Each trial site will recruit 10 to 50 patients (see Appendix II).

Study duration

The duration of treatment will be 18 months resulting in duration of the trial of about 24 months; 1 month screening, 3 for recruitment, 18 months for the treatment period, and 2 for an observational follow-up period after the interventional period.

Study collective

Eligible patients must meet the following requirements: Possible, probable (clinically or laboratory) or definite ALS with a disease duration > 6 months and < 3 years, treatment with riluzole > 3 month, best sitting FVC >50% of predicted normal, onset of progressive weakness within 36 months prior to study.

Justification for gender selection

Pioglitazone is prescribed to male and female patients with type 2 diabetes. In this study male and female patients should be enrolled. The proportion of the genders is not determined and occurs by chance. A selection of the gender of the patients participating in this trial is not necessary, because male and female patients have to fulfil the same inclusion criteria.

Investigational medicinal product (IMP), dosage and administration

The IMP will be pioglitazone in a dose of 45 mg. As comparative preparation matching placebo will be used. The study medication (IMP or placebo) will be provided as identical tablets and has to be taken by the patients once daily and orally. The dosage will be increased step wise: 15 mg once daily will be applied during the first 2 weeks, 30 mg once daily during week 3 and 4 and 45 mg once daily from week 5 on.

Efficacy variables

<u>Primary</u>

Survival in patients treated with pioglitazone compared to placebo

<u>Secondary</u>

- Incidence of tracheotomy or non-invasive ventilation
- ALS Functional Rating Scale (ALSFRS-R; the revised version including the respiratory parameters)
- Quality of life using the Euroqol EQ-5D

Safety variables

- Physical examination including vital signs (BW, BH, BMI, BP, HR)
- Safety laboratory parameters (blood cell count; clinical chemistry including glucose, ASAT, ALAT, CK, creatinine)
- Frequency, relationship and seriousness of adverse events (AEs)

Statistical methods

The primary endpoint will be subjected to a confirmatory analysis. All secondary and safety parameters will be evaluated and reported descriptively.

All individual data as well as results of statistical analyses – whether explicitly discussed in the following sections or not – will be presented in individual subject data listings and statistical summary tables.

Continuous variables will be summarised using the following standard descriptive summary statistics: number of observations, arithmetic mean, standard deviation, minimum, lower quartile, median, upper quartile, maximum. In addition, geometric mean and coefficient of variation derived from log-normally distributed data will be calculated for serum concentrations. Categorical data will be described using absolute and relative frequencies.

Schedule of study procedures

Visits	Screening (V0)	Baseline (V1)	V2	T1	V3	T2	V4	T3	V5	T4	V6
Months		Inclusion	1	1	2	3	6	9	12	15	18
Informed consent	X										
Inclusion/non-inclusion criteria checking	X	X									
Medical history	X										
Vital capacity	X										
Status (El Escorial Criteria)	X										
Concomitant treatment	X	X		X	X	X	X	X	X	X	X
Physical examination	X	X			X		X		X		X
Weight	X			X		X		X		X	
ALS FRS-R		X		X	X	X	X	X	X	X	X
Euroqol EQ-5D		X			X		X		X		X
Study drug delivery		X			X		X		X		
Drug accountability					X		X		X		X
Laboratory tests	X	X	X		X		X		X		X
Pregnancy test (serum HCG level)	X										
Compliance (pioglitazone level)		X			X		X		X		X
Compliance (asked for)				X		X		X		X	
Adverse events		X		X	X	X	X	X	X	X	X

2 BACKGROUND AND RATIONALE

2.1 Name and description of the investigational product

Pioglitazone treatment improves insulin sensitivity by a novel mode of action, the activation of PPARγ-receptors, in animal models of type 2 diabetes with insulin resistance. The improved insulin sensitivity results in a lowering of glucose and insulin concentrations in non-insulinopenic diabetic animals, but does not affect on those levels in normal animals. Therefore, this compound does not have a potential for hypoglycaemia and can avoid the adverse consequences of long-term hyperinsulinaemia.

Pioglitazone has been shown to lower hyperglycaemia, triglycerides and non-esterified fatty acids (NEFA) and increases insulin sensitivity in animal models of type 2 diabetes mellitus but affects neither normoglycaemia in normal rats and dogs nor hyperglycaemia in insulin-deficient rats treated by streptozocin.

The potential for pioglitazone to elicit unwanted pharmacological effects on the central nervous, cardiovascular, autonomic, renal and digestive systems has been investigated in detail and there is a satisfactory margin of safety with respect to clinically attainable plasma concentrations of pioglitazone.

The maximum dose of pioglitazone administered in clinical trials is 60 mg od, which was well tolerated. There was no evidence of a reduction in blood glucose in healthy volunteers but significant reductions in blood glucose were observed throughout the clinical studies conducted in patients with type 2 diabetes.

Following oral administration, pioglitazone is rapidly absorbed and peak concentrations of unchanged pioglitazone are usually achieved 2 hours after administration. Proportional increases of the plasma concentration were observed for doses from 2 mg – 60 mg. Steady state is achieved after 4-7 days of dosing. Repeated dosing does not result in accumulation of the compound or metabolites. Absorption is not influenced by food intake. Absolute bioavailability is greater than 80 %.

2.2 Findings from non-clinical and clinical studies relevant to the trial

The primary pathological feature of ALS is the loss of motor neurons[1], which is accompanied by a robust glial response including activation of microglia and astrocytes as well as the expression of COX-2 and iNOS in the spinal cord[2-5]. Both pathologies, motor neuron loss and neuroinflammation can be found in transgenic mice over-expressing mutant variants of the human gene encoding for copper/zinc superoxide dismutase (SOD1), which have been linked to inherited ALS[6-9].

The massive appearance of activated microglia and astrocytes already at an early, pre-symptomatic stage of disease in SOD1 transgenic mice suggests that inflammation may contribute to motor neuron degeneration and suppression of the inflammatory component could be neuroprotective. PPARγ activation in microglia and macrophages results in inhibition of pro-inflammatory gene expression

through various mechanisms[10-12]. It has been demonstrated that PPARγ agonists reduce amyloid beta peptide and cytokine mediated neuroinflammation and neurotoxicity in vitro[13,14] and in vivo[15].

Originally developed as oral antidiabetics, agonists of PPARγ have been found to exert potent anti-inflammatory effects in several models of neuroinflammation[10,16]. Importantly, pioglitazone-mediated reduction of inflammation was neuroprotective in the SOD1-G93A transgenic mouse model of ALS as reported independently by two groups[17,18]. Within the thiazolidinedione class of PPARγ agonists, pioglitazone is the only substance, which penetrates the blood brain barrier to a significant extent[19] and has therefore been chosen for these animal experiments. The results strongly suggest that pioglitazone-mediated suppression of inflammation may offer a new therapeutic avenue in ALS treatment, as supported by findings in animal models of multiple sclerosis[20-22], Parkinson’s disease[23], stroke[24], and Alzheimer’s disease[25,26].

2.3 Known and potential risks and benefits, if any, to human subjects

The clinical trial programme showed that pioglitazone is well tolerated. With pioglitazone treatment, oedema can occur but is not usually a cause of withdrawal. Weight may increase during treatment, most likely as a result of increases in subcutaneous fat. Despite weight gain the effects of pioglitazone to improve glycaemic control and lipid profile were maintained. There was no evidence of any toxic effect of pioglitazone on the liver. Increases in CPK and LDH can occur transiently during treatment with pioglitazone but are not of clinical significance.

Pioglitazone has been prescribed to over 3.5 million patients worldwide with over 2 million patient years of usage. The majority of use has been in USA. In Europe, it is estimated that more than 200,000 patients have been prescribed the drug on the market.

Liver toxicity similar in severity and outcome to that reported from the market with troglitazone has not been reported with pioglitazone. In some cases of hepatic reactions, including hepatitis, a role for pioglitazone in their aetiology cannot be ruled out but their nature and incidence is similar to those also reported with other oral hypoglycaemic agents including the sulphonylureas and metformin.

Cases of congestive heart failure (CHF) have been reported in temporal association with pioglitazone use. However, since the background rate of this condition is so high in type 2 diabetes (2-3 cases per 100 per year as reported in the literature), it is not possible to ascribe a clear role for pioglitazone in the development of CHF.

Post-marketing surveillance has thus far not detected any life-threatening suspected ADRs with clear causal relationship to pioglitazone not seen in the clinical trials and has therefore confirmed the ADR profile of pioglitazone reported from clinical trials.

Pioglitazone is contraindicated in patients with:

- Known hypersensitivity to pioglitazone or to any of the excipients of the tablet

- Cardiac failure or history of cardiac failure (NYHA stages I to IV)
- Hepatic impairment

Pioglitazone is also contraindicated for use in combination with insulin.

Expected Adverse Events: Anaemia, back pain, cardiac failure, CPK increased, fatigue, headache, haematocrit decreased, haemoglobin decreased, hepatic congestion, hepatocellular dysfunction, LDH increased, leg cramps, liver enzymes increased, myalgia, pharyngitis, oedema (generalised and peripheral), RBC decreased, sinusitis, tooth disorder, upper respiratory tract infection, urinary tract infection, weight increased, resumption of ovulation, vision abnormal, flatulence

2.4 Description of and justification for the route of administration, dosage, dosage regimen, and treatment period(s)

The route of administration (oral) is supported by established clinical experience with pioglitazone. Dosage is built on the comparison of effects and pharmacokinetics in men and mice. The dosage regimen is based on the experience with the use of pioglitazone as an antidiabetic. Therefore, in a limited degree we can extrapolate the data from diabetes treatment to ALS.

3 STUDY PURPOSE, TRIAL OBJECTIVES AND ENDPOINTS

As the efficacy of riluzole to prolong survival in ALS is limited, there is an urgent need to identify other compounds and to evaluate their potential in clinical studies. The pharmacological profile of pioglitazone suggests that this compound may be effective in slowing down the neurodegenerative process observed in ALS and it is therefore important to study this drug in ALS patients. As a significant percentage of patients with ALS will receive riluzole, the study population will consist of patients stabilised on standard riluzole therapy at time of inclusion. After completing this study, patients may have the option to participate in another planned, longer, safety study with pioglitazone.

This study will evaluate the efficacy, safety and tolerability of pioglitazone (45 mg/day) versus placebo in patients with ALS receiving standard, approved therapy (riluzole, 50 mg bid). Mortality defined exclusively as death represents the primary endpoint. Secondary efficacy endpoints will include incidence of tracheotomy or non-invasive ventilation (NIV), ALS Functional Rating Scale (ALSFRS-R; the revised version including the respiratory parameters) and measurement of quality of life using the Euroqol EQ-5D. Assessment of clinical tolerability and safety will be performed by comparison of findings on physical examination, adverse events incidence, and clinical laboratory values across treatment groups.

4 SUBJECT SELECTION

4.1 Trial population

The population consists of 220 enrolled ALS patients as defined by the El Escorial criteria, and modified by the Airlie House criteria (http://www.wfnals.org).

4.2 Trial admission

A patient will only be enrolled in the study after obtaining his or her written consent to participation. The investigator must first provide a complete explanation of the study (benefits, risks, rights and obligations) and check that the patient satisfies all the inclusion criteria and none of the exclusion criteria.

4.3 Selection criteria

The following inclusion and exclusion criteria are believed to represent the ALS population; in particular they have been thoroughly discussed by European experts (including the coordinating investigator) in a conference held in the European Neuromuscular Center (ENMC) Naarden, Holland in October 2003. They were used in a similar way in the previous large European ALS trials (Exonhit, Ono, Novartis); the coordinating investigator was involved in the protocol development of each of these trials.

4.3.1 Inclusion criteria

To be considered eligible to participate in this trial a patient must meet the inclusion criteria listed below.

- Possible, probable (clinically or laboratory) or definite ALS according to the revised version of the El Escorial World Federation of Neurology criteria
- Disease duration more than 6 months and less than 3 years (inclusive). Disease onset defined as date of first muscle weakness, excluding fasciculation and cramps
- Best-sitting SVC between 50 % and 95 % of predicted normal
- Continuously treated with 100 mg riluzole daily, for at least 3 months
- Capable of thoroughly understanding all information given and giving full informed consent according to GCP
- Women of childbearing age must be non-lactating and surgically sterile or using a highly effective method of birth control and have a negative pregnancy test. Acceptable methods of birth control with a low failure rate i.e. less than 1% per year) when used consistently and correct are such as implants, injectables, combined oral contraceptives, hormonal intrauterine devices (IUDs), or double-barrier methods (condom or diaphragm with spermicidal agent or IUD), sexual abstinence or vasectomised partner.
- Onset of progressive weakness within 36 months prior to study

Written informed consent must be obtained from all patients prior to any study-related procedure.

4.3.2 Exclusion criteria

In addition, to be eligible for entry into the trial, a patient may *not* meet the exclusion criteria listed below.

- Previous participation in another clinical study within the preceding 12 weeks
- Tracheotomy or assisted ventilation of any type during the preceding three months
- Gastrostomy
- Any medical condition known to have an association with motor neuron dysfunction which might confound or obscure the diagnosis of ALS
- Presence of any concomitant life-threatening disease or impairment likely to interfere with functional assessment
- Confirmed hepatic insufficiency or abnormal liver function (ASAT and/or ALAT > 1.5 upper limit of normal)
- Renal insufficiency (serum creatinine > 2.26 mg/dL)
- Evidence of major psychiatric disorder or clinically evident dementia precluding evaluation of symptoms
- Known hypersensitivity to any component of the study drugs
- Likely to be not cooperative or comply with the trial requirements (as assessed by the investigator), or unable to be reached in the case of an emergency
- Other antidiabetics
- Heart failure or heart failure in the patients history (NYHA I to IV)
- History of macular oedema
- Treatment with thiazolidinediones within 3 months prior to screening
- Known or suspected history of alcohol and/or drug abuse
- Treatment with gemfibrozil within 3 months prior to screening

4.4 Protocol violations

Except in the case of a medical emergency, no protocol violation is authorised outside amendments made by the sponsor and validated by an IEC/IRB. Protocol violations may affect the conduct of the study from legal and ethical points of view and also influence the statistical analysis and pertinence of the study. If a medical emergency or event occurs in a patient leading to a protocol violation this will

only be allowed for this patient. The investigator must contact the sponsor to see if the patient may continue the study. Any protocol violation must be reported on the case report form.

5 TRIAL DESIGN

5.1 Design overview

This is a prospective, multicentre, randomized, stratified, parallel-group, double-blind trial comparing placebo with 45 mg pioglitazone as add-on therapy to 100 mg riluzole in amyotrophic lateral sclerosis (ALS) in 220 enrolled patients. For entry, the El Escorial Criteria for the diagnosis of ALS will be used (http://www.wfnals.org). The patients have to be stable on riluzole at least three months prior to randomization. The trial requires an initial screening visit (V0), one baseline visit (V1), 4 subsequent control visits (V2 – V5), and a final visit (V6). The duration of study participation for patients completing the study is 18 months.

After patients are screened for inclusion and exclusion criteria, they are randomly assigned to treatment with either placebo or 45 mg pioglitazone according to their stratum (see section 6.10.1). The dosage will be increased step wise: 15 mg once daily will be applied during the first two weeks, 30 mg once daily during week 3 and 4 and 45 mg once daily from week 5 on up to the end of the study. The study drug will be tablets of 15/30/45 mg base or matching placebo. The drug will be administered orally. In patients with gastrostomy, tablets will be crushed and administered via the feeding tube.

5.2 Trial procedures

On enrolment in the trial, a patient will undergo a physical examination and a detailed medical history will be obtained.

Efficacy Variables

Efficacy variables will be determined at the study site by using standard methods.

- Survival / Date of death requiring a death certificate
- ALS-FRS-R
- Euroqol EQ-5D
- Tracheotomy or NIV (yes/no, date of event, reason for intervention, duration per day, if applicable)

Safety Variables

- Blood cell count (RBC, WBC, Hb, Hct, MCV, MCH, MCHC)
- ASAT
- ALAT
- CK

- Creatinine
- Fasting blood glucose
- Beta-HCG

Table 5.1: Blood Samples and Volumes

SAFETY	Sample Volume (ml)	V0	V1	V2 (GP)	V3	V4	V5	V6
		Scree-ning	Day 1	Month 1	Month 2	Month 6	Month 12	Month 18
blood cell count	EDTA (2.7)	X	X		X	X	X	X
ALAT	Serum (4.9)	X	X	X	X	X	X	X
ASAT		X	X	X	X	X	X	X
CK		X	X	X	X	X	X	X
Creatinine		X	X	X	X	X	X	X
Fasting blood glucose	NAF (1.2)	X	X	X	X	X	X	X
beta-HCG	Serum (1.1) or urine	X						
Pioglitazone-level	Serum (9.0)		X		X	X	X	X
**Total Blood Volume (ml)**		9.9	17.8	4.9	17.8	17.8	17.8	17.8

The calculated volume of blood needed for the different laboratory determinations at the different visits is given in table 5.1. The maximal calculated volume of blood taken of patients who have completed the whole study is approximately 105 ml within 18 months.

Any deviation of laboratory parameters from normal ranges will be documented and followed up as appropriate. Clinically significant deviations observed in the screening visit and during the study will be documented in the Case Report Form.

Adverse Events

Adverse events observed by the investigators or reported by the patients will be documented as described in chapter 7.

Demographic Data

- Date of Birth
- Sex
- Origin
- Familial status

Medical History

Clinically significant conditions that stopped within the last 5 years and concomitant diseases will be documented. In addition, following items have to be addressed specifically:

- Smoking habits within the last 12 months
- Alcohol consumption

Physical Examination

- Height
- Weight
- Radial pulse (with the patient in sitting position for at least 5 minutes)
- Blood pressure (according to Riva Rocci using calibrated sphygmomanometers, the patient has to be in a sitting position for at least 5 minutes, blood pressure will be measured twice within 5 minutes, the mean of both measurements will be calculated)
- Any abnormalities detected in:

  - Head and neck
  - Ear, nose, throat
  - Eyes
  - Cardiovascular system
  - Respiratory system
  - Abdomen
  - Extremities and back
  - Lymph nodes
  - Skin
  - Neurological
  - other relevant abnormalities

5.3 Trial visits schedule

For the planned trial visits schedule the inclusion date is considered to be the reference date. The dates of the following visits specified in the protocol are calculated in relation to the date of this visit.

If the time of a visit is modified in relation to the visits schedule, the times of the following visits will always be determined with respect to the inclusion visit. The time window for each planned visit will be

± one week. The investigator must always ensure that the patient receives a sufficient quantity of treatment units to cover the period up to the next visit. The visits schedule is not modified by any unscheduled visits.

In the case of premature interruption of the study treatment, a complete examination identical to visit V6 should be carried out. Premature study discontinuation must be noted as such on the case report form. Patients prematurely leaving the study must not rejoin the trial.

Additionally a study physician will contact the patient by telephone 1, 3, 9, and 15 months after study enrolment.

An overview including the time schedule for all visits is given in table 5.2.

Screening Visit (V0)

A signed “Informed Consent” will be obtained from each patient prior to initiating any trial procedure. Presence of ALS diagnostic criteria will be documented as well as site of onset and ALS status at entry according to El Escorial criteria, and modified by the Airlie House criteria (http://www.wfnals.org). Venous blood will be taken for the determination of blood cell count, creatinine, CK, ASAT, ALAT, fasting blood glucose, and HCG or urine pregnancy test (only in women with child bearing potential). The patients´ eligibility will be determined in accordance with the inclusion/exclusion criteria. A detailed medical history will be taken, and concomitant medication will be recorded. A physical examination, including body weight, height, blood pressure, and radial pulse will be performed. Additionally lung function will be assessed by measuring the patient’s vital capacity.

Visit 1 – Baseline visit (V1)

Patients eligible for study participation will enter the study site within 4 weeks after the screening visit and will be randomised to one of the treatment arms.

Blood samples for the determination of blood cell count, creatinine, CK, ASAT, ALAT, fasting blood glucose, and pioglitazone baseline level will be obtained. A physical examination, ALS FRS-R, and EuroQol EQ-5D will be performed. The patient will receive study medication according to the randomization list and a study participant card (see appendix IX).

Any changes in the concomitant medication and adverse events before and during visit 1 will be documented.

Visit 2 – Control for ASAT and ALAT at the patient’s general practitioner (V2)

Visit 2 will be performed one month after V1.

The patient’s general practitioner will collect blood from the patient to determine creatinine, CK, ASAT, ALAT, and fasting blood glucose. Values will be transmitted to the local study site by fax.

Visit 3-5 – Control Visits (V3, V4, V5)

Visit 3, 4, and 5 will be performed 2, 6, and 12 months after visit 1 respectively.

Venous blood samples will be taken for the determination of blood cell count, creatinine, CK, ASAT, ALAT, fasting blood glucose, and pioglitazone level. A physical examination, ALS FRS-R, and EuroQol EQ-5D will be performed. Drug accountability will be performed and the patient will be supplied with new study medication. Any changes in the concomitant medication and adverse events will be documented.

Visit 6 – Final Visit (V6)

Visit V6 will be performed 18 months after visit V1. Any adverse events and changes in concomitant medication will be documented.

Blood samples will be taken for the determination of blood cell count, creatinine, CK, ASAT, ALAT, fasting blood glucose, and pioglitazone level. Patients will undergo a physical examination and ALS FRS-R and EuroQol EQ-5D will be performed. The patient will return the study medication of V5 and drug accountability will be performed.

Telephone contacts (T1 – T4)

At months 1, 3, 9, 15, or if a visit has been missed, the patient or its relative will be contacted by telephone to assess the following items:

- ALSFRS-R
- Weight
- Adverse events
- Changes in concomitant therapies
- Compliance to study drug regimen

Table 5.2: Study Schedule

Visits	Screening (V0)	Baseline (V1)	V2	T1	V3	T2	V4	T3	V5	T4	V6
Months		Inclusion	1	1	2	3	6	9	12	15	18
Informed consent	X										
Inclusion/non-inclusion criteria checking	X	X									
Medical history	X										

Visits	Screening (V0)	Baseline (V1)	V2	T1	V3	T2	V4	T3	V5	T4	V6
Vital capacity	X										
Status (El Escorial Criteria)	X										
Concomitant treatment	X	X		X	X	X	X	X	X	X	X
Physical examination	X	X			X		X		X		X
Weight	X			X		X		X		X	
ALS FRS-R		X		X	X	X	X	X	X	X	X
Euroqol EQ-5D		X			X		X		X		X
Study drug delivery		X			X		X		X		
Drug accountability					X		X		X		X
Laboratory tests	X	X	X		X		X		X		X
Pregnancy test (serum HCG level or urine test)	X										
Compliance (pioglitazone level)		X			X		X		X		X
Compliance (asked for)				X		X		X		X	
Adverse events		X		X	X	X	X	X	X	X	X

5.4 Unscheduled visits

Unscheduled visits may be carried out at any time during the study in the case of a medical emergency, when a patient would like to report a medical problem or when the investigator considers this necessary for the patient’s well being. These unscheduled visits must be recorded on the case report form. In no case must they lead to a modification in the study visits schedule stipulated by the protocol.

5.5 Visits following premature discontinuation of study treatment

If a patient discontinues participation in the study for whatever reason, every effort has to be undertaken to perform an early termination visit. In this case, the same investigations have to be performed as in visit V6.

5.6 Sequence and duration of trial periods

The duration of treatment will be 18 months resulting in a duration of the trial of about 24 months; 1 month will be reserved for screening, three for recruitment, 18 months for the treatment period, and two for an observational follow-up period after the interventional period. Then the functional and survival data will be analysed.

6 STUDY TREATMENTS

6.1 *Test preparations*

Investigative Products

Pioglitazone Tablets

INN:	Pioglitazone
Trade name:	Actos
Dosage:	Tablets containing 15 mg, 30 mg, and 45 mg pioglitazone respectively
Daily dose:	1 tablet/day oral in the morning before breakfast
Manufacturer:	Haupt Pharma Brackenheim GmbH, Germany
Batch No.:	See trial master file

Placebo (3 dosages for pioglitazone)

INN:	Not applicable
Trade name:	Not applicable
Dosage:	Not applicable
Daily dose:	1 tablet/day oral in the morning before breakfast
Manufacturer	Haupt Pharma Brackenheim GmbH, Germany
Batch No.:	See trial master file

6.2 *Treatment packaging*

Haupt Pharma, Brackenheim/Germany will supply the study drugs. The study drugs will be packed in accordance with AMG. All drug supplies must be stored in accordance with the manufacturer's instructions and must be stored separately from normal clinic (study centre) stocks. The investigator shall be responsible for assuring that the study medication is stored in a dry location and not above 25 C, protected from exposure to any environmental changes and is locked, so that only the investigator and specifically designated other persons can have access.

6.3 *Treatment labelling*

According to article 5 of the German GCP-V the labelling of investigational drugs contains at minimum the following information

Outer packaging

- Name and address of the Sponsor and the CRO
- Name and dosage of the test compound
- Lot-No.
- Formulation (e.g. tablet or capsule etc.)
- Content by weight, volume or number of units
- Administration route
- Expiry date
- Only for use in clinical trial
- Storage condition
- Keep away from children
- Contains lactose
- Patient ID
- Protocol ID, EudraCT No.
- Stratum (bulbar onset or spinal onset)

Blister

- Name and address of the Sponsor and the CRO
- Lot-No.
- Protocol ID
- Patient ID
- Administration route

6.4 Storage of drug supplies

The study drug will be directly sent to the study site. The investigator will confirm receipt of the first and all subsequent batches of study drugs in writing to Haupt Pharma Brackenheim GmbH. All drug supplies must be stored in accordance with the manufacturer's instructions, and separately from normal clinic (study centre) stocks. At the study site, the investigator is responsible for assuring that the study medication is stored properly, protected from exposure to any environmental changes and is locked, so that only the investigator and specifically designated other persons can have access.

6.5 *Treatment assignment*

The investigator must only give the study treatments to the patients included in the trial according to the procedures stipulated by the protocol. It is strictly prohibited to use these treatments for any other reason than that described in the protocol. The medication will be given to the patient directly by the investigator. If the patient misses a visit, the medication can be given to a relative or can be sent.

6.6 *Treatment administration*

Pioglitazone (oral 15 to 45 mg /day; uptake: 1-0-0, before breakfast; tablets include 15, 30 or 45 mg pioglitazone)

V1 (Baseline):

A) 1 blister with 14 tablets pioglitazone 15 mg,
1 blister with 14 tablets pioglitazone 30 mg,
2 blister with 14 tablets pioglitazone 45 mg

B) 1 blister with 14 tablets placebo matching pioglitazone 15 mg,
1 blister with 14 tablets placebo matching pioglitazone 30 mg,
2 blister with 14 tablets placebo matching pioglitazone 45 mg

V2 (Month 1):

No study drug delivery, medication included in V1 package

V3 (Month 2):

A) 8 blister with 14 tablets pioglitazone 45 mg

B) 8 blister with 14 tablets placebo matching pioglitazone 45 mg

V4.1 (Month 6), V4.2 (Month 9), V5.1 (Month 12), V5.2 (Month 15):

A) 6 blister with 14 tablets pioglitazone 45 mg

B) 6 blister with 14 tablets placebo matching pioglitazone 45 mg

Medication for reserve (two packages: one for V1, one for V4.2)

A) 2 blister with 14 tablets pioglitazone 45 mg

B) 2 blister with 14 tablets placebo matching pioglitazone 45 mg

6.7 *Overdose*

There is no specific advice in case of an overdose. General measures should be taken.

6.8 Compliance

The investigator will select patients who have the ability to understand and comply with instructions for trial participation. Non-compliant participants will be dropped from further participation in the trial. Compliance of the patient will be judged by the investigator. Additionally, the study drugs will be dispensed exclusively by the investigator or a designate. All unused study medication will be returned to the investigator. Drug accountability including tablet counts will be performed by the investigator.

Patients will be asked to return their unused study medication including the empty blisters and boxes at each visit. To assess compliance, the remaining tablets will be counted out of the patient’s presence. At visit V1, V3, V4, V5, and V6 pioglitazone levels will be measured in the serum of each patient to control compliance. Patients, who have taken more than 120% or less than 80 % of the medication during the treatment period, may continue the trial but will not be included in the per protocol analysis.

6.9 Concomitant treatment

Patients will be allowed to continue to use any concomitant medications that they require except medication indexed in the exclusion criteria (as per Summary of product characteristics). If medically acceptable all concomitant medications should be kept constant during the investigation. If a change of concomitant medication is necessary, the investigator has to be informed.

Change of concomitant medication will be documented in the case report form.

6.10 Randomisation and unblinding procedures

6.10.1 Randomisation

At the screening visit (V0), each patient will receive the next consecutive screening number from a block of screening numbers according to their stratum (bulbar onset vs. spinal onset). Patients will be assigned to the bulbar or spinal stratum according to the location of the earliest experienced ALS symptom (defined by the first muscle weakness, or in the case of bulbar onset, by the presence of dysarthria and/or dysphagia). In the case of a patient with simultaneous onset of spinal and bulbar symptoms, onset will be defined as bulbar. Cervical and respiratory onsets are stratified to the spinal-onset stratum. This stratum assignment must be consistent with the diagnosis and clinical assessment (i.e., maximum score on bulbar scale and low score on manual muscle testing would contradict the assignment to bulbar stratum).

At the randomization visit (V1), each patient eligible for study participation will receive the next consecutive randomisation/patient number according to his stratum from a block of randomisation numbers per site. The randomisation list will be generated by Haupt Pharma Brackenheim, Germany, using a validated system, which involves a pseudo-random number generator to ensure that the resulting treatment sequence will be both reproducible and non-predictable. Study medication will be packed and blinded by Haupt Pharma Brackenheim, Germany, according to the randomisation list. Each patient medication box will be sent together with the sealed unblinding codes to the sites. The

investigator at the site takes care that each patient will be provided with the study medication box of the correct randomisation number.

6.10.2 Blinding

Blinding of investigator and patient is achieved by providing pioglitazone and matching placebo tablets.

The randomisation list is kept confidential by Haupt Pharma Brackenheim, Germany, and is only accessible to authorised personnel until the code is broken. At the end of the trial, any emergency opening of the envelopes will be controlled after collecting the explanations for unblinding and checking the unused treatment units. The code will be broken only at the end of the study after freezing the statistics database (by checking the data, recording any protocol violations etc.) allowing the collected data to be analysed.

6.10.3 Emergency procedure for unblinding

Each study site will receive sealed unblinding envelopes for emergency cases. Haupt Pharma Brackenheim will send the envelopes to the sites together with the matching medication.

In case of an emergency situation the investigator has the right to open the unblinding envelope, but every effort should be taken to avoid unblinding of patients except the information will be needed for the emergency treatment. Opened envelopes have to be signed, dated and the reason for unblinding has to be documented. The envelope has then to be filed in the patient file and the sponsor or the responsible monitor has to be informed. Unblinded patients have to be withdrawn from the study.

Additionally the sponsors' safety contact will receive a full set of sealed unblinding envelopes for emergency cases. As well every effort should be taken to avoid unblinding of patients except the information will be needed for the emergency treatment. Opened envelopes have to be signed, dated and the reason for unblinding has to be documented.

No other reason than an emergency may justify unblinding. After unblinding the investigator must note the date, time and reason on the case report form. The investigator must also immediately inform the trial monitor of the breaking of the blind.

6.11 Drug accountability

The investigator must keep a precise record of the dispatch and dispensing of trial medication in a drug accountability record. At all times during the study it must be possible to control that the date of dispensing and the quantity of trial medication given to each subject has been noted.

The investigator must not destroy any label or any box of medication even if only partly used. At the end of the study the investigator must group together all the boxes of used or unused treatments for recovery by the study monitor.

7 ADVERSE EVENTS

7.1 Definitions

7.1.1 Adverse event (AE)

The term adverse event describes any untoward medical occurrence in a patient or a clinical investigation subject, which does not necessarily have a causal relationship with the study treatment.

This definition includes:

- Any sign or symptom occurring during the study
- Any event or disease present on the day of inclusion in the trial with symptoms that worsen during the study
- Any accident
- Any significant change in laboratory parameters
- Any reaction to drug withdrawal
- Any drug interaction
- Any effect related to overdose, abuse or dependence

This definition also applies to events occurring in any comparative group (active treatment or placebo) or during the placebo-treatment phase or during periods when the patient is not receiving any study medication.

Note: the term study treatment refers to all the drugs studied including the reference drugs and any other treatment stipulated in the protocol.

7.1.2 Serious adverse event (SAE)

A serious adverse event is any untoward medical occurrence that at any dose:

- Results in death,
- is life-threatening,
- requires in-patient hospitalisation or prolongation of existing hospitalisation,
- results in persistent or significant disability/incapacity,
- is a congenital anomaly, birth defect, or
- may require intervention, as a medically important event, to prevent one of the above criteria or may expose the patient to danger, even though the event is not immediately life threatening or fatal or does not result in hospitalisation (examples: allergic bronchospasm treated on an outpatient basis, convulsions not requiring hospitalisation or abnormal laboratory parameters compromising patient safety).

The following points should be taken into account when evaluating the seriousness of an adverse event:

- The life-threatening criterion indicates that there was an immediate risk of death at the time of its occurrence. This does not mean that if the adverse event had occurred in a more severe form it would have lead to death.

The following are not considered to be serious adverse events:

- An event causing a brief visit to a hospital consultation, an open-door or day hospital,
- outpatient treatment in the emergency department although the reason for which this treatment was instituted may be serious, or
- admission to hospital (more than one night in a hospital bed) including surgical operations planned before or during the study if the condition was present before the study and provided that it does not worsen during the study.

The following are considered to be serious adverse events even if they do not comply with the definition:

- Adverse events caused by misuse or overdose,
- Any pregnancy discovered during the study.

Note: a state of pregnancy is an exclusion criterion. Hence contraceptive measures are necessary in women with childbearing potential throughout the study. However if pregnancy is discovered during the study the trial treatment must immediately be discontinued and the pregnancy followed to term.

N.B. a non-serious adverse event designates any other event not corresponding to the above-defined criteria.

7.1.3 Non Serious Adverse Events

Non serious adverse events mean adverse event, which are not categorised as serious adverse event.

7.1.4 Severity (Intensity) and outcome

The severity of an adverse event represents the intensity of the event as reported by the patient or assessed by the investigator. It does not reflect the clinical seriousness of the event (for example severe nausea, minor stroke). The intensity is evaluated independently from the relation to the study treatment.

- Mild: the adverse event is transient and easily tolerated by the patient.
- Moderate: the adverse event causes the patient discomfort and interference with the patient's usual activities.
- Severe: the adverse event causes considerable interference with the patient's usual activities

The outcome of each AE must be assessed according to the following classification:

- completely recovered :	The patient has fully recovered with no observable residual effects
- not yet completely recovered :	Improvement in the patient’s condition has occurred, but the patient still has some residual effects
- deterioration :	The patient’s overall condition has worsened
- permanent damage :	The AE has resulted in a permanent impairment
- death :	The patient died due to the AE
- ongoing :	The AE has not resolved
- unknown :	The outcome of the AE is not known because the patient did not return for follow-up (lost to follow-up)

7.2 Monitoring and recording of adverse events

At each visit after V0 the investigator must question the patient about the occurrence of any adverse event and note any abnormal laboratory findings. A neutral question such as “since the last visit have you felt any untoward or unusual symptoms other than those related to your illness?” elicits any adverse events experienced by the patient.

Whatever the relation to the study treatment is, any adverse event such as those described in section 7.1 spontaneously reported by the patient or observed by the investigator must be reported on the appropriate page of the case report form. For each adverse event, the following information should be documented:

- Description of the event (diagnosis and symptoms in verbatim terms)
- Outcome
- Seriousness
- Duration (start date and stop date)
- Severity (mild, moderate or severe); see section 7.1.4
- Frequency (permanent, intermittend)
- Causal relationship with the study treatment according to the scale shown in section 7.3
- Corrective actions including measures taken for the study treatment

The study number, patient number and investigator’s signature must also be noted.

Any clinically significant event including any abnormal laboratory result or vital sign must be followed-up until it has completely disappeared or the investigator has assessed its sequelae, even after the end of the study.

The results of any complementary tests performed (laboratory tests, ECG, X-rays etc.) must be dated and appended to the CRF. The investigator must evaluate each adverse event and give his/her opinion about the causal relationship with the drug(s) used.

A period of 14 days for follow up of AEs after the patient has routinely or prematurely terminated the study seems sufficient.

7.3 Assessment of adverse events

7.3.1 Evaluation criteria

The relation between the adverse event and the study medications must be evaluated according to the data collected. Causality assessment will be made by the investigator for both non serious and serious AEs using the 4-category system defined below.

(1) DEFINITE

An adverse event that follows a reasonable temporal sequence from the administration of the drug (including the course after the withdrawal of the drug) and that satisfies any of the following:

- Reappearance of similar reaction upon re-administration (re-challenge)
- Positive results in drug sensitivity tests (skin test…)
- Toxic levels of the drug revealed by measurement of drug concentrations in blood or other body fluids.

(2) PROBABLE

An adverse event that follows a reasonable temporal sequence from the administration of the drug (including the course after the withdrawal of the drug) and for which factors other than the drug, such as underlying diseases, complications, concomitant drugs and concurrent treatments can reasonably be excluded.

(3) POSSIBLE

An adverse event, that follows a reasonable temporal sequence from the administration of the drug (including the course after the withdrawal of the drug) and for which possible involvement of the drug can be argued (*), although factors other than the drug, such as underlying diseases, complications, concomitant drugs and concurrent treatments may also be responsible.

> (*) For example, there have been similar reports in the past including reports on its analogues or the occurrence of the event could be predicted from the pharmacological actions / chemical structure of the drug.

(4) UNLIKELY / NOT RELATED

An adverse event that does not follow a reasonable temporal sequence from the administration of the drug or that can be reasonably explained by other factors such as underlying disease, complications, concomitant drugs and concurrent treatments.

Note: if any essential information for the evaluation including temporal sequence of the adverse event from the administration of the drug (including the course after withdrawal of the drug), underlying diseases, complications, concomitant drugs and concurrent treatments is not available, the case can be cited as "lack of data" at the initial report. However, the adverse event shall be categorised into any of (1)-(4) as soon as possible.

7.3.2 Points to consider

The following points must also be taken into account during causality assessment of adverse events:

- Timing of the event between administration of the drug and the onset of the adverse event
- Drug levels and evidence, if any, of overdose
- A known or expected response pattern to the suspect drug. This included:
    - Previous experience with the drug and whether the adverse event is known to have occurred with the drug
    - Physiological effect of the drug
    - Is the adverse event known as an adverse event related to other drugs, which belongs to the same or to similar class?
    - Can the event be explained by the pharmacological action or with regard to the findings in animal?
    - Has the patient a specific genetic predisposition (e.g. slow acetylizer)?
- Dechallenge, i.e. when the drug was decreased or stopped, what happened to the adverse event? This includes was the event cured by giving an antidote?
- Rechallenge i.e. when the drug was restarted after the adverse event had disappeared, what happened?
- Alternative explanations for the adverse event, such as the use of other drugs, illness, non-drug therapies, diagnostic tests, procedures or other confounding factors. Did the adverse event also happen with placebo?

7.4 Documentation and reporting of serious adverse events

7.4.1 Reporting responsibility

After signature of the consent form, any serious adverse event occurring, while the patient is in the study or during the 2 weeks after the end of the study, must be reported by the investigator.

7.4.2 Reporting procedures

1- SERIOUS ADVERSE EVENT INITIAL REPORT

As soon as the investigator has been informed about a serious adverse event, he or she should fill in as precisely as possible the serious adverse event report form and assess the responsibility of the study treatment.

The investigator must fax the form **within one working day** of knowledge of the event to the Safety Contact. This form and the fax machine receipt must be kept in the case report form.

If the event is unexpected and fatal/life-threatening and is considered by the investigator related to the study treatment, the Safety Contact should be informed **immediately** by phone. If necessary, the other investigators participating in the trial will be informed about the occurrence of such a serious adverse event.

The investigator will report any **serious adverse event** to the sponsor of the study (safety contact):

Prof. Jan Kassubek, MD, Department of Neurology, University of Ulm/Germany
Phone: +49 (0)731 177-1206 Fax: +49 (0)731 177-1202

Both the investigator and the sponsors safety contact will adhere to the GCP-requirements (§§ 12, 13) on documentation and notification.

Suspected unexpected serious adverse reactions (SUSARs) have to be reported to the responsible Ethics Committee at the University of Ulm by the sponsors' safety contact within the defined notification periods according to GCP-V §13 Abs. 2 and 3.

In addition, the sponsors' safety contact will inform the Competent Authorities within the defined notification periods. The other investigators participating in the trial will be informed about the occurrence of such a SUSAR.

In this trial on the fatal disease ALS, the mortality is the efficacy endpoint and the integrity of the trial may be compromised when the blind is systematically broken for reporting of mortality related SUSARs. Therefore each mortality case will be treated as disease-related and will not be subject to systematic unblinding and expedited reporting. The reporting of these events will be completed within one month after last patient's last visit. The independent Data Safety and Monitoring Board (DSMB) will review the safety data every three month in the ongoing trial with recommendation to the sponsor whether to continue, modify or terminate the trial. All cases of SUSARS that are not efficacy endpoints will be reported as usual.

2- SERIOUS ADVERSE EVENT FOLLOW-UP REPORT

If new information becomes available after transmission of the initial report, a follow-up report should be completed by using the same form. Follow-up information shall be handled in the same way as the initial information (i.e. within one working day of knowledge). A copy of any hospital reports and results of any lab tests must also be attached.

7.4.3 Responsible Nominated Safety Contact

The fax and telephone numbers of the Responsible Nominated Safety Contact are given in the list provided in the present protocol. Additionally the independent Data Monitoring and Safety Committee (DMSC) will have to be informed if an SAE occurs.

7.5 Unblinding of treatment / Emergency identification

In the case of a medical emergency requiring identification of the treatment taken by the patient, the code may be unblinded. For more information see section 6.10.3 "emergency unblinding procedure".

7.6 Premature discontinuation / Patient replacement

Each patient can at any time withdraw from the study without personal disadvantages and without stating reasons.

Whenever possible, all patients prematurely discontinuing study medication will continue to be followed and complete all scheduled evaluations for the duration of the study. The reason(s) for premature cessation of a patient's participation during the trial, other than death, should be specified on the case report form. Whenever possible the evaluations scheduled for the end of the treatment period should be performed.

Study treatment may be stopped prematurely if any of the following events occur, according to the judgement of the investigator:

- Significant intolerance of the study medication, or significant clinical or laboratory findings, e.g. increase in ASAT or ALAT >2.5 upper limit of normal
- Significant intercurrent illness or emergency situation requiring cessation of the study
- The patient wishes to terminate his/her participation in the study
- Investigator judgement that it is in the best interest of the patient
- Failure to comply with the investigational procedures
- Pregnancy
- Technical reasons (change of physician, change of address of volunteer/patient)

Patients who have started double-blind treatment will not be replaced.

7.6.1 Investigator's responsibility following premature discontinuation of patients

For any patient prematurely discontinuing the study, the investigator must:

- Recover all the treatment units given to the patients
- In as far as possible, carry out all the examinations and tests planned for the final visit after withdrawal of the subject from the study
- Fill in the corresponding pages of the case report form, specifying the date and reason for premature discontinuation
- When applicable, ask the patient the reason of his/her Informed Consent withdrawal while fully respecting the subject's rights
- Where possible prescribe a visit 2 weeks after withdrawal in order to control and document any change in the patient’s clinical status. If a serious adverse event occurs, the Responsible Nominated Safety Contact must be informed according to the procedures described in section 7.4 “Documentation and reporting of serious adverse events”.

7.6.2 Study Termination

Certain circumstances may lead to early termination of an entire study, in particular for ethical or safety reasons such as:

- The high frequency and/or unexpected severity of adverse events,
- Unsatisfactory recruitment of patients in as far as their quantity or quality is concerned or recurrent incomplete/inappropriate collection of data.

The study may be also stopped prematurely if the interim analysis shows pronounced superiority of the intervention group compared to the placebo group (s. section 8.2.5). The decision to prematurely terminate the study will be taken by the sponsor and the investigator.

8 STATISTICS

8.1 Sample size and power considerations

In four previous ALS trials in which the principal investigator was involved, the rate of loss to follow up was less than 1 % (Exonhit/Pentoxifyllin, ONO2506, 5000 mg Vitamin E trial, TCH346-Novartis-Trial). We expect to repeat this low rate, since ALS is a life threatening disease and the centres involved are experienced in the clinical care for the patients.

The study will be conducted using a two-stage group sequential test design within the Δ-class of critical values according to Wang and Tsiatis.[27] The planned information rates are 0.5 and 1. These information rates fix the weights for combining the p-values using the weighted inverse normal method.[28] The critical values of the group sequential test design were calculated for the standardised (cumulative) test statistic for the design with boundary shape parameter $\Delta = 0.0$, i.e. O’Brien/Fleming type design. For one-sided

$\alpha = 0.025$ and the information rates given above the resulting boundary p-values are given by p1 = 0.00258 and p2 = 0.02400 with corresponding critical values 2.797 and 1.977, respectively.

For one-sided $\alpha = 0.025$ and 18-month survival rates of 60% in the standard group and 78% in the test group (hazard ratio = 0.486)[29], the statistical power is 80% if the log-rank test is performed after observation of a total of 31 deaths at the first stage and after 61 deaths (accumulated number) at the second (final) stage (formula of Schoenfeld, ADDPLAN® version 3.1.2, ADDPLAN GmbH, Germany).

Assuming an accrual time of 6 months and a follow-up time of 15 months (21 months observation time) a total of 198 subjects (99 subjects per treatment group, without drop-outs) is expected to yield the necessary number of deaths if the accrual rate is constant (conservative estimation). Under these assumptions, the time points of analyses are expected to be 11.1 months observation time (interim analysis) and 21 months observation time (final analysis).

Thus, considering a drop-out rate of 10%, it is planned to enrol a total of 220 patients.

8.2 Statistical methods

A Statistical Analysis Plan describing all details of the analyses to be performed will be prepared by the study statistician and approved by the sponsor prior to the start of statistical analysis. The recommendations of the ICH E9 Note for Guidance on Statistical Principles for Clinical Trials (CPMP/ICH/363/96, 1998) will be taken into account.

The primary endpoint will be subjected to a confirmatory analysis. All secondary and safety parameters will be evaluated and reported descriptively.

All individual data as well as results of statistical analyses – whether explicitly discussed in the following sections or not – will be presented in individual subject data listings and statistical summary tables.

Continuous variables will be summarised using the following standard descriptive summary statistics: number of observations, arithmetic mean, standard deviation, minimum, lower quartile, median, upper quartile, maximum. In addition, geometric mean and coefficient of variation derived from log-normally distributed data will be calculated for serum concentrations. Categorical data will be described using absolute and relative frequencies.

A more detailed elaboration of the principal statistical features stated in this protocol will be provided in the statistical analysis plan, which will be finalized prior to unblinding of the database. The statistical analysis plan will take into account any amendment to the study protocol.

8.2.1 Populations

The following data sets will be analysed:

- Full-analysis set:

  All randomised patients who received at least one dose of study medication will be included in the all patients treated set.

- Per-protocol analysis set:

  The per protocol analysis set will consist of all patients in the full analysis set without any major protocol violations. Patients with major protocol violations will be excluded from the per-protocol analysis. The classification of major and minor protocol deviations and the resulting definition of analysis sets will be performed prior to the final analysis and will be approved by the sponsor.

All analyses with respect to safety and tolerance will be performed for the full-analysis set. All efficacy analyses will be conducted for the full-analysis set and for the per-protocol analysis set. The primary data set for the efficacy analysis will be the full-analysis set.

A more detailed description of the criteria used for data sort out will be included in the statistical analysis plan before start of the evaluations prior to unblinding the treatment allocation.

8.2.2 Background and demographic characteristics

Assessments made at the screening (V0) and randomization visit V1 will be summarised by treatment group and overall. These assessments will include demographic characteristics and other relevant parameters (such as medical history and physical examination). By-treatment summaries will serve to identify any imbalances between the treatment groups before start of active treatment.

Previous / concurrent diseases will be coded and presented according to the MedDRA terminology.

Summary tables will be provided for the the full-analysis set and for the per-protocol analysis set by means of descriptive statistics and frequency tables where appropriate.

8.2.3 Study medication

Compliance with respect to study medication will be computed based on the available data of the number of dispensed and returned study medication and the number of days the patient should have taken medication (dispensing intervals).

8.2.4 Concomitant therapy

Concomitant medications will be tabulated by generic drug name and Anatomical Therapeutic Chemical (ATC) code for each treatment group.

8.2.5 Efficacy evaluation

The primary objective of the study is to demonstrate the superiority of the test treatment compared to the standard treatment in terms of survival.

The study will be conducted according to a two-stage group sequential test design with possible sample size adaptation after the planned interim analysis. The overall type-I-error rate is set at $\alpha = 0.025$ (one-sided).

The following null hypothesis H_0 will be tested against the alternative hypothesis H_A ($\alpha = 0.025$, one-sided):

H_0: $\lambda 2 / \lambda 1 \geq 1$

H_A: $\lambda 2 / \lambda 1 < 1$

where $\lambda 2 / \lambda 1$ is the hazard ratio, $\lambda 1$ denotes the hazard in the standard group and $\lambda 2$ in the test group. The hazard ratio is assumed to be constant across time.

The null hypothesis will be tested using the log-rank test.

The first interim analysis will be conducted after observation of a total of 31 deaths. The null hypothesis will be rejected at the first interim analysis if the calculated test statistic exceeds the critical value 2.797. In this case, the study may be stopped. If the null hypothesis will not be rejected, the study may be continued until the pre-specified number of 61 deaths (accumulated number of deaths) or a recalculated number of deaths based on the effect size estimation of the first interim analysis were observed.

Data from all subjects will be considered when calculating the second stage log-rank test statistic. At the final (second) analysis, the null hypothesis will be rejected if the calculated inverse normal test statistic exceeds the critical value 1.977.

This procedure preserves the overall (experiment-wise) type I error rate of $\alpha = 0.025$ in a strong sense.

For estimating the treatment effect, the hazard ratio and the corresponding two-sided 95% repeated confidence interval will be provided.

All other group comparisons are hypothesis generating in nature, i.e., p-values resulting from these statistical tests will be interpreted in the exploratory sense.

8.2.6 Safety evaluation

The full-analysis set will be used to address issues of safety. Adverse events data will be processed in the statistical analysis after coding according to the MedDRA dictionary.

Adverse events (AEs) will be classified as pre-treatment AEs or treatment-emergent AEs. Analyses will be performed for these classes. Pre-treatment AEs are defined to be AEs, which obviously occurred prior to the first administration of study medication. Treatment-emergent AEs are defined to be AEs, which occurred at or after the first administration of study medication. In case of doubt regarding the timing of AE onset the worst-case scenario will be applied, i.e. the AE will be considered as treatment-emergent.

AEs will be analysed regarding their seriousness, severity, outcome and causality assessment. SAEs will be analysed regarding their severity, outcome and causality assessment. Furthermore, all AEs with possible, probable or definite causal relationship to study medication (causality assessment) will be summarised as ‘drug-related’ AEs. They will be analysed regarding severity and outcome. All analyses will report frequency counts of subjects by MedDRA SOC and preferred term. Furthermore, treatment-emergent AEs will be presented by preferred term and sorted by frequency.

Listings by subject will be provided for SAEs and for AEs.

8.2.7 Interim analysis

The study will be conducted according to a two-stage group sequential test design with possible sample size adaptation after the interim analysis. The interim analysis is planned after observation of 31 deaths (expected observation time of 11.1 months). No per-protocol analysis will be performed for the interim evaluation of efficacy and safety.

At the planned interim analysis, the random code of the subjects to be included in the interim analysis will be unblinded by a member of the DMSC. Before unblinding a Statistical Analysis Plan will be prepared for the interim analysis by the unblinded statistician and agreed on between the sponsor and the unblinded statistician. The unblinded statistician will electronically match the random code with the study data and will send confidentially the results and a brief statistical report summarising the results of the interim analysis to the DMSC.

8.2.8 Other evaluations

Subgroup Analyses

Subgroup analyses will be performed for the bulbar and spinal stratum.

Multicentre Data

Exploration of possible heterogeneity of treatment effects across centres will be performed in a descriptive manner if appropriate, e.g. by graphical display of the results of the individual centres.

To assess the homogeneity of treatment differences across centres for the primary efficacy variable, treatment-by-centre interaction will be evaluated.

8.3 Data handling and record keeping

The investigator must maintain all study records, patient files and other source data for the maximum period of time permitted by the hospital or institution. The data from the individual centre will be transferred to the coordinating centre. They will be monitored at the site and with the help of source documents.

Data processing will be conducted on the basis of the Data Management Plan approved by the sponsor prior to any data management activity. The data cleaning process will be performed according to the Data Validation Plan. The database will be designed by acromion GmbH using the acromion DMS based on Sybase.

Data will be entered into the database using the technique of independent double data entry with a third person-compare where two people enter the same data independently and a third person resolves any discrepancies. Changes made to the data will be documented in an audit trail.

The following international dictionaries will be used for medical coding in their current installed version at the beginning of coding activities, ensuring that there is only one version used during the study:

- Diseases: Medical Dictionary for Regulatory Activities (MedDRA)
- Adverse events: Medical Dictionary for Regulatory Activities (MedDRA)
- Drugs: WHO-DRL dictionary

The statistical analysis will be performed using the software package SAS version 9.1 (SAS Institute Inc., Cary, NC 27513, USA).

All available data will be included in the analyses and will be summarized as far as possible. Unless otherwise specified there will be no substitution of missing data, i.e. missing data will not be replaced, missing data will be handled as 'missing' in the statistical evaluation.

In the case of a premature termination, the last available observation will be used for analysis (LOCF approach), if applicable and considered reasonable. Missing baseline data will not be replaced. A more detailed description of the handling of missing data will be provided in the statistical analysis plan after review of the data for the interim and the final analysis.

9 ETHICAL ASPECTS

This protocol is in accordance with the principles laid down by the 18th World Medical Assembly (Helsinki, 1964) and its amendments laid down by World Medical Assemblies. This protocol is also in accordance with laws and regulations of the country in which the trial is performed, as well as any applicable guidelines. It is the responsibility of the investigator to obtain informed consent in compliance from each patient prior to entering the trial or, where relevant, prior to evaluating the patient's suitability for the study.

9.1 Independent Ethics Committee Approval

Before implementing this study, the protocol, the proposed informed consent form and other information to subjects, must be reviewed by a properly constituted Independent Ethics Committee / Institutional Review Board (IEC/IRB). A signed and dated statement that the protocol and informed consent have been approved by the IEC/IRB must be given to the sponsor before study initiation. The name and occupation of the chairman and the members of the IEC/IRB must be supplied to the sponsor.

9.2 Informed Consent

The investigator must ensure that before enrolment in the study, all eligible patients are given detailed information both orally and in writing, about the following points in particular:

- Aims, duration and methods of the study
- Risks, anticipated benefits and potential hazards according to our current knowledge

- That he or she is free to withdraw his or her consent at any time without affecting subsequent treatment or his/her relation with the doctor
- That the clinical research monitors and auditors appointed by the sponsor, Health Authority Inspectors and IEC/IRB members have access to the clinical source data about them.

Informed consent must be given by a standard written declaration drafted in simple non-technical language. The patient must read and examine the contents of this form before signing and dating it and he or she must be given a signed copy. No patient may be enrolled in a study before giving his or her informed consent.

The investigator may withdraw the trial drug at any time if he considers that this is in the patient's interest. The patient also has the right to withdraw his or her consent at any time (for the procedure to be followed in the case of premature discontinuation, see section 7.6 "Premature discontinuation").

The investigator must accept to inform all the patients included in the study about any new information on the trial drug discovered during the study in accordance with the recommendations of the sponsor and the IEC/IRB.

9.3 Data confidentiality

All the data collected will be processed in a manner guaranteeing the strictest confidentiality. Patients will be informed that the data collected during the study will be archived and processed in accordance with local individual data protection law.

Patients will also be informed that clinical research monitors appointed by the sponsor, Health Authority Inspectors and IEC/IRB members may only obtain access to the source clinical data to check the data in the case report form. This point must be explicitly stated on the consent form.

The investigator must hold an updated list of all patients selected/enrolled in the study by noting the patient's initials in the same way as in the case report form together with the treatment and randomisation number where applicable.

9.4 Insurance

A specific insurance was contracted to cover the civil liability of the investigators and sponsors working in therapeutic biomedical research.

10 ADMINISTRATIVE PROCEDURES

By signing the study protocol, the investigator accepts to comply with the following points:

10.1 Regulatory aspects

The study will be conducted in compliance with the current provisions of the German Drug Law and GCP Regulation (GCP-V), the respective decrees and the European Clinical Trial Directive. Regulatory reporting requirements will be agreed on by the parties in the investigator contract.

10.2 Quality Assurance

The sponsor has to implement Quality Control and Quality Assurance systems to ensure that the study will be conducted according to Good Clinical Practices.

10.2.1 Monitoring

Organization and monitoring of the study will be performed by acromion GmbH, Frechen, Germany, under the responsibility of the study sponsor. The study sites have to be monitored regularly. A monitoring guideline will be established by acromion before the initiation visit. The monitoring visits will take place in regular intervals to ensure that the study will be conducted in compliance with the GCP guidelines.

The study monitor is responsible for setting up the study and follow-up (visits, telephone calls, letters, and fax).

The study monitor will visit study centres in order to check that the case report forms are filled in without omission. The monitor will also control the progress of patient inclusion and make sure that the study treatments are stored, dispensed and accounted for according to the specifications given. The investigator must be available to help the clinical monitor during visits.

The monitor is responsible for reviewing it and clarifying and resolving any data queries during his/her next visit on site. The completed and corrected case report forms for completed visits will be collected by the monitor. A copy of the case report form must be kept by the investigator, who will ensure that it is kept safely with the other study documents such as the protocol, investigator's brochure and any protocol amendments.

Any suspicion of fraud has to be documented and the monitor has to inform the coordinating investigator and / or the sponsor. An audit of the centre will be performed as soon as possible to support or invalidate the suspicion. Based on the audit findings, the coordinating investigator will take appropriate measures.

10.2.2 Data Monitoring and Safety Committee (DMSC)

An independent Data Monitoring and Safety Committee (DMSC) consisting of clinical and statistical experts will be established by the sponsor to review the interim efficacy and safety results. The DMSC meets regularly at three months intervals and if necessary. The DMSC will have access to unblinded data. Based on the results of the interim analysis the DMSC will give written recommendations to the sponsor to continue, to modify, to put on hold or to stop the trial.

The DMSC will act according to its own SOP and will prepare written minutes of its meetings. In order not to disseminate unblinded data and to ensure that all staff involved in the conduct of the study remains blind to the results of the interim analysis, only the members of the DMSC and the unblinded statistician will have access to these data.

10.2.3 Source Data Definition and Verification (SDV)

Source data are the original records of all variables collected for the trial. They include, but are not limited to:

- Signed informed consent
- Laboratory reports
- Individual patient clinical notes
- Hospital charts or pharmacy records and any other similar reports and records of any procedure performed in accordance with the protocol
- Details concerning inclusion and exclusion criteria
- Appropriate sections of the CRFs, where data is recorded directly onto specific forms

The CRF is the source document for all data where no other source exists such as body weight, blood pressure and physical examination.

The investigator must allow access to the important documents in the medical file to confirm their consistency with the case report form entries. No information on patient identity is allowed in these documents. The following must be checked: existence of informed consent, adherence to in-/exclusion criteria, tabulation of adverse events and completion of the primary safety and efficacy variables. Other checks that the data given in the case report forms complies with the source document will be carried out in accordance with the study specific monitoring plan.

10.2.4 Data recording

All data reported in the case report forms must be clearly noted with a black ballpoint pen so that carbon copies or photocopies are legible. Corrections must be made by crossing out the incorrect term with a single horizontal line so that it remains legible with the correct term next to it. This correct term must be dated and accompanied by the investigator’s initials. The use of correction fluid is prohibited

All the information stipulated in the protocol must be provided and any omission must be explained.

10.2.5 Retention of documents

The investigator must draft and keep complete and precise documents on the study in compliance with the German Drug Law and Good Clinical Practices. The study documents comprise the following:

- Case report forms
- Data correction forms
- Source documents (e.g. laboratory sheets)
- Monitoring cards
- Appointment agendas

- Correspondence between sponsor and investigator
- Regulatory documents such as:
  - Signed protocol and any amendments.
  - IEC/IRB approval and other letters from this committee
  - Curriculum Vitae of medical personnel (investigators, co-investigators, nurses etc.)
  - Patient Signed Consent forms
  - Investigator's convention and agreement
  - Study treatment accounting forms

The investigator is responsible for keeping the source documents for each patient participating in the trial with at least the study number marked on them.

The protocol, case report forms, correspondence, original informed consent form, patient identification log (name, first name, file number…) and other documents on the conduct of the study must be kept by the investigator according to the local regulations. No trial document may be destroyed without written agreement of the sponsor and/or project leader. The retention period for all documents at all trial sites is 15 years including the trial master file at the University of Ulm.

10.2.6 Auditing procedures

Rules of Good Clinical Practice have to be considered in their totality and national or foreign inspectors will be allowed access to original documents including patient files at all times during and after the end of the study. The investigator will accept these requirements by participating in the trial. If patient files are stored on a computer system, the investigator has to ensure that the data will be saved and printed on signed and dated paper. The project leader, clinical research assistants, quality assurance personnel, or health authorities (CA, ICC/IRB) have the right of access to the source documents and the right to check that Good Clinical Practices are respected throughout the study conduct. Health authorities may also request the right to carry out an inspection during the study or after it has ended.

10.3 Disclosure and Confidentiality

10.3.1 Confidentiality of data

The investigator must ensure to keep the entire study information and documents strictly confidential and to demand the same discretion from the complete study team.

Patient data noted in the case report forms during the study must be kept pseudonymously, i.e. the patient will only be identifiable by random/patient number, and date of birth. If it becomes necessary under exceptional circumstances for administrative or safety reasons to disclose the patient's identity, this information must also be kept strictly confidential.

10.4 Protocol amendments

It is specified that the appendices attached to this protocol and referred to in the main text of this protocol, form an integral part of the protocol. No changes or amendments to this protocol may be made by the Investigator or the Steering Committee after the protocol has been agreed to and signed by both parties unless such change(s) or amendment(s) have been fully discussed and agreed upon by the Investigator and the SC. Any change agreed upon will be recorded in writing; the written amendment will be appended to this protocol. Approval/advice of amendments by the Ethics Committees is required prior to their implementation, unless there are overriding safety reasons. Prior to initiating the changes, protocol amendments must be submitted to competent authority (BfArM), where applicable, except under emergency conditions.

A copy of the written approvals will be given to the investigators.

Amendments involving only administrative items will be sent to the IEC/IRB and the competent authority for information only.

10.5 Clinical trial report and publication

The results of the study will be reported in a Clinical Study Report generated by the coordinating investigator according to Good Clinical Practices and ICH recommendations.

The Steering committee will be responsible for timely publication of the results. It is the goal of the coordinating centre to make sure that either positive or negative results will be distributed internationally. The results of the trial, may they be negative or positive will be presented on internationally meetings; a paper is to be published on the international level. The funding agencies will be informed in the study report.

11 REFERENCES

1. Talbot,K. Motor neurone disease. *Postgrad. Med. J.* **78**, 513-519 (2002).
2. Almer,G. *et al.* Increased expression of the pro-inflammatory enzyme cyclooxygenase-2 in amyotrophic lateral sclerosis. *Ann. Neurol.* **49**, 176-185 (2001).
3. Yasojima,K., Tourtellotte,W.W., McGeer,E.G. & McGeer,P.L. Marked increase in cyclooxygenase-2 in ALS spinal cord: implications for therapy. *Neurology* **57**, 952-956 (2001).
4. Phul,R.K., Shaw,P.J., Ince,P.G. & Smith,M.E. Expression of nitric oxide synthase isoforms in spinal cord in amyotrophic lateral sclerosis. *Amyotroph. Lateral. Scler. Other Motor Neuron Disord.* **1**, 259-267 (2000).
5. Barbeito,L.H. *et al.* A role for astrocytes in motor neuron loss in amyotrophic lateral sclerosis. *Brain Res. Brain Res. Rev.* **47**, 263-274 (2004).
6. Kunst,C.B. Complex genetics of amyotrophic lateral sclerosis. *Am. J. Hum. Genet.* **75**, 933-947 (2004).

7. Gurney,M.E. *et al.* Motor neuron degeneration in mice that express a human Cu,Zn superoxide dismutase mutation. *Science* **264**, 1772-1775 (1994).

8. Hensley,K. *et al.* Temporal patterns of cytokine and apoptosis-related gene expression in spinal cords of the G93A-SOD1 mouse model of amyotrophic lateral sclerosis. *J. Neurochem.* **82**, 365-374 (2002).

9. Yoshihara,T. *et al.* Differential expression of inflammation- and apoptosis-related genes in spinal cords of a mutant SOD1 transgenic mouse model of familial amyotrophic lateral sclerosis. *J. Neurochem.* **80**, 158-167 (2002).

10. 1Landreth,G.E. & Heneka,M.T. Anti-inflammatory actions of peroxisome proliferator-activated receptor gamma agonists in Alzheimer's disease. *Neurobiol. Aging* **22**, 937-944 (2001).

11. Daynes,R.A. & Jones,D.C. Emerging roles of PPARs in inflammation and immunity. *Nat. Rev. Immunol.* **2**, 748-759 (2002).

12. Blanquart,C., Barbier,O., Fruchart,J.C., Staels,B. & Glineur,C. Peroxisome proliferator-activated receptors: regulation of transcriptional activities and roles in inflammation. *J. Steroid Biochem. Mol. Biol.* **85**, 267-273 (2003).

13. Heneka,M.T. *et al.* Peroxisome proliferator-activated receptor gamma agonists protect cerebellar granule cells from cytokine-induced apoptotic cell death by inhibition of inducible nitric oxide synthase. *J Neuroimmunol.* **100**, 156-168 (1999).

14. Combs,C.K., Johnson,D.E., Karlo,J.C., Cannady,S.B. & Landreth,G.E. Inflammatory mechanisms in Alzheimer's disease: inhibition of beta-amyloid-stimulated proinflammatory responses and neurotoxicity by PPARgamma agonists. *J Neurosci.* **20**, 558-567 (2000).

15. Heneka,M.T., Klockgether,T. & Feinstein,D.L. Peroxisome proliferator-activated receptor-gamma ligands reduce neuronal inducible nitric oxide synthase expression and cell death in vivo. *J Neurosci.* **20**, 6862-6867 (2000).

16. Feinstein,D.L. Therapeutic potential of peroxisome proliferator-activated receptor agonists for neurological disease. *Diabetes Technol. Ther.* **5**, 67-73 (2003).

17. Schutz,B. *et al.* The oral antidiabetic pioglitazone protects from neurodegeneration and amyotrophic lateral sclerosis-like symptoms in superoxide dismutase-G93A transgenic mice. *Journal of Neuroscience* **25**, 7805-7812 (2005).

18. Kiaei,M., Kipiani,K., Chen,J.Y., Calingasan,N.Y. & Beal,M.F. Peroxisome proliferator-activated receptor-gamma agonist extends survival in transgenic mouse model of amyotrophic lateral sclerosis. *Experimental Neurology* **191**, 331-336 (2005).

19. Maeshiba,Y. *et al.* Disposition of the new antidiabetic agent pioglitazone in rats, dogs, and monkeys. *Arzneimittelforschung.* **47**, 29-35 (1997).

20. Feinstein,D.L. *et al.* Peroxisome proliferator-activated receptor-gamma agonists prevent experimental autoimmune encephalomyelitis. *Ann. Neurol.* **51**, 694-702 (2002).

21. Niino,M. *et al.* Amelioration of experimental autoimmune encephalomyelitis in C57BL/6 mice by an agonist of peroxisome proliferator-activated receptor-gamma. *J. Neuroimmunol.* **116**, 40-48 (2001).

22. Natarajan,C., Muthian,G., Barak,Y., Evans,R.M. & Bright,J.J. Peroxisome proliferator-activated receptor-gamma-deficient heterozygous mice develop an exacerbated neural antigen-induced Th1 response and experimental allergic encephalomyelitis. *J. Immunol.* **171**, 5743-5750 (2003).

23. Breidert,T. *et al.* Protective action of the peroxisome proliferator-activated receptor-gamma agonist pioglitazone in a mouse model of Parkinson's disease. *J. Neurochem.* **82**, 615-624 (2002).

24. Sundararajan,S. *et al.* Peroxisome proliferator-activated receptor-gamma ligands reduce inflammation and infarction size in transient focal ischemia. *Neuroscience* **130**, 685-696 (2005).

25. Heneka,M.T. *et al.* Acute treatment with the PPAR gamma agonist pioglitazone and ibuprofen reduces glial inflammation and A beta 1-42 levels in APPV717I transgenic mice. *Brain* **128**, 1442-1453 (2005).

26. Sastre,M. *et al.* Nonsteroidal anti-inflammatory drugs repress beta-secretase gene promoter activity by the activation of PPAR gamma. *Proceedings of the National Academy of Sciences of the United States of America* **103**, 443-448 (2006).

27. Wang,S.K. & Tsiatis,A.A. Approximately optional one-parameter boundaries for group sequential trials. *Biometrics* **4**, 193-199 (1987).

28. Lehmacher,W. & Wassmer,G. Adaptive sample size calculations in group sequential trials. *Biometrics* **5**, 1286-1290 (1999).

29. Meininger,V., Asselain,B., Guillet,P., Leigh,P.N., Ludolph,A., Lacomblez,L. & Robberecht.W. Pentoxifylline European Group. Pentoxifylline in ALS: a double-blind, randomized, multicenter, placebo-controlled trial. *Neurology* **66** (1), 88-92 (2006)

12 APPENDICES

Appendix I	Declaration of Investigators
Appendix II	Designated Study Sites and Recruitment Plan
Appendix III	Steering Committee
Appendix IV	Data Safety and Monitoring Board (DSMB)
Appendix V	Sample of SAE Report Form
Appendix VI	Declaration of Helsinki
Appendix VII	Extract from the German Drug Law (AMG §§ 40 – 42)
Appendix VIII	German GCP-Regulation (GCP-Verordnung)
Appendix IX	Study Participant Card

APPENDIX I Declaration of Investigators

DECLARATION OF INVESTIGATORS

I have read this trial protocol and agree that it contains all the information required to conduct the trial. I agree to conduct the trial as set out in this protocol. I will not enrol the first patient in the trial until I have received approval from the appropriate ethics committee where applicable, and until all legal requirements in my country have been fulfilled. I agree to obtain, in the manner described in the trial protocol written informed consent to participate from all patients enrolled in this study and to keep the consent forms for 15 years. I will provide my curriculum vitae before the trial starts.

Place, date	Signature Principal Investigator
Name (printed)	Address (stamp / printed)

APPENDIX II Designated Study Sites and Recruitment Plan

Designated Study Sites and Recruitment Plan

The following centres agreed to participate in the trial and to recruit 10 to 50 patients. All sites have been selected for their successful recruitment of patients in ALS studies in the past ten years. Furthermore a study group was established with the objective to conduct trials in ALS patients using neuroprotective agents

Site 01
Ulm
N = 50

Prof. A. C. Ludolph, MD
Department of Neurology
University of Ulm
Oberer Eselsberg 45
89081 Ulm
Phone: +49 731 177 1200
Fax: +49 731 177 1206
Email: albert.ludolph@rku.de

Site 02
Berlin
N = 10

Prof. T. Meyer, MD
Department of Neurology
Humboldt University
Augustenburger Platz 1
13353 Berlin
Phone: +49 30 450 560 032
Fax +49 30 450 560 938
Email: thomas.meyer@charite.de

Site 03
Bochum
N = 10

Torsten Grehl, MD
Facharzt für Neurologie
Neurologische Universitätsklinik Bergmannsheil
Bürkle-de-la-Camp-Platz 1
44789 Bochum
Phone: +49 234 302 6812
Fax: +49 234 302 6888
Email: Torsten.Grehl@rub.de

Site 04
Bonn
N = 10

Prof. Michael Heneka, MD
Klinik und Poliklinik für Neurologie
Universitätsklinikum Bonn
Sigmund-Freud-Str. 25
53105 Bonn
Phone: +49 228 287 15750
Fax: +49 228 287 15024
Email: Michael.Heneka@ukb.uni-bonn.de

Site 05
Dresden
N = 20

Prof. Alexander Storch, MD
Technische Universität Dresden
Department of Neurology
Fetscherstr. 74, Haus 25
01307 Dresden
Phone: +49 351 458 3908
Fax: +49 351 458 6387
Email: Alexander.Storch@neuro.med.tu-dresden.de

Site 06
Erlangen
N = 10

Prof. D. Heuss, MD
Department of Neurology
University of Erlangen
Schwabachanlage 6
91054 Erlangen
Phone: +49 9131 85 36939
Fax +49 9131 85 34844
Email: dieter.heuss@neuro.med.uni-erlangen.de

Site 07
Göttingen
N = 26

PD Jochen Weishaupt, MD
Abteilung für Neurologie
Universitätsmedizin Göttingen
Robert-Koch-Str. 40
37099 Göttingen
Phone: +49 551 39 14343
Fax +49 551 39 12932
Email: jochen.weishaupt@medizin.uni-goettingen.de

Site 08
Halle / Saale
N = 12

Prof. Stephan Zierz, MD
Department of Neurology
University of Halle Wittenberg
Ernst-Grube-Str. 40
06097 Halle / Saale
Phone: +49 345 557 2858
Fax +49 345 557 2860
Email: Stephan.zierz@medizin.uni-halle.de

Site 09
Hannover
N = 36

Prof. Reinhard Dengler, MD
Director of the Department of Neurology
Medical School Hannover (MHH)
Carl Neuberg Str. 1
30625 Hannover
Phone: +49 511 532 23 91
Fax: +49 511 532 3115
Email: Dengler.Reinhard@mh-hannover.de

Site 10
München
N = 30

Prof. Gian Domenico Borasio, MD
ALS-Ambulanz - Neurologische Klinik und
Interdisziplinäres Zentrum für Palliativmedizin
Klinikum der Universität München-Großhadern
Marchioninistraße 15
81366 München
Phone: +49 89 7095 4931
Fax: +49 89 7095 7929
Email: Borasio@med.uni-muenchen.de

Site 11
Münster
N = 15

Prof. Peter Young, MD
Department of Neurology
University of Münster
Albert-Schweitzer-Str. 33
48129 Münster
Phone: +49 251 83 48331
Fax: +49 251 83 45059
Email: Young@uni-muenster.de

Site 12
Regensburg
N = 10

Prof. Dr. Ulrich Bogdahn, MD
Klinik und Poliklinik für Neurologie
der Universität Regensburg
Im Bezirksklinikum
Universitätsstraße 84
93053 Regensburg
Phone: +49 941 94 -8418
Fax: +49 941 941-3005
Email:

Site 13
Rostock
N = 10

Prof. Reiner Benecke, MD
Klinik und Poliklinik für Neurologie
Universitätsklinikum Rostock
Gehlsheimer Str. 20
18147 Rostock
Phone: +49 381 494-4742
Fax: +49 0381 494-4792
Email:

Site 14
Wiesbaden
N = 10

Berthold Schrank, MD
Deutsche Klinik für Diagnostik
Fachbereich Neurologie / Klin. Neurophysiologie
Aukammallee 33
65191 Wiesbaden
Phone: +49 611 577-431
Fax: +49 611 577-311
Email: mailto:schrank.neuro@dkd-wiesbaden.de

**Site 15**	Marcus Beck, MD
Würzburg	Department of Neurology
N = 15	University of Würzburg
	Josef-Schneider-Str. 11
	91054 Würzburg
	Phone: +49 931 201 23751
	Fax: +49 931 201 23697
	Email: m.beck@klinik.uni-wuerzburg.de

**Site 16**	Julian Grosskreutz, MD
Jena	Friedrich-Schiller-Universität<br>Universitätsklinikum<br>Klinik Berger-Klinik für Neurologie
N = 10	Erlanger Allee 101
	07747 Jena
	Phone: +49 3641 932-3453
	Fax: +49 3641 932 -3422
	Email: julian.grosskreutz@med.uni-jena.de

APPENDIX III Steering Committee

Steering Committee

The members of the Steering Committee are:

Prof. Reinhard Dengler, MD
Director of the Department of Neurology
Medical School Hannover (MHH)
Carl-Neuberg-Strasse 1
30625 Hannover
Phone: +49 511 – 532 23 – 91

Prof. M. T. Heneka, MD
Department of Neurology
University of Münster
Albert-Schweitzer-Str. 33
48129 Münster
Phone: +49 251 – 834 80 – 16

Prof. A. C. Ludolph, MD
Department of Neurology
University of Ulm
Oberer Eselsberg 45
89081 Ulm
Phone: +49 731 – 177 -1200

Prof. T. Meyer, MD
Department of Neurology
Humboldt University
Augustenburger Platz 1
13353 Berlin
Phone: +49 30 – 450 560 – 032

Prof. Stephan Zierz, MD
Department of Neurology
University of Halle Wittenberg
Ernst-Grube-Str. 40
06097 Halle / Saale
Phone: +49 345 - 557 2858

APPENDIX IV Data Safety and Monitoring Board (DSMB)

Data Safety and Monitoring Board (DSMB)

The designated members of the DSMB are:

Prof. Jan Kassubek, MD

Department of Neurology

University of Ulm

Oberer Eselsberg 45

89081 Ulm

Phone: +49 731 – 177 -1206

Fax: +49 731 – 177 -1202

Prof. Michael Schwarz, MD

Department of Neurology

Klinikum Dortmund gGmbH

Beurhausstr. 40

44137 Dortmund

Phone: +49 231 – 953 21810

Fax: +49 231 – 953 21039

Prof. Wilhelm Gaus, PhD

Im Riedle 12

89134 Blaustein

Phone: +49 7304 – 41194

Fax: +49 7304 – 928491

APPENDIX V Sample of SAE Report Form

Addressee for reporting<br>**PD Jan Kassubek, MD**<br>**Dept. of Neurology, University of Ulm**<br>**Oberer Eselsberg 45, D-89081 Ul**<br>**Tel.: +49 731-177 1206 FAX: +49 731-177 1202**	Trial No.<br>**GERP-ALS**	Screening No.:	Randomisation No.:
	Country: **GERMANY**		

Sex	Date of Birth	Height	Weight	Investigator, Location
☐ Male ☐ Female		kg	kg	

CROSS THE BOX(ES) APPROPRIATE TO SAE

☐ Death ☐ Involved or prolonged patient hospitalisation ☐ Congenital anomaly

☐ Life-threatening ☐ Involved persistence of significant disability or incapacity ☐ Required intervention to prevent permanent damage/impairment

NATURE OF SAE

Diagnosis(es) (if no diagnosis is available, give main symptoms)

	**Intensity**	**Date of Onset of symptoms**<br>Day Month Year	**Continuing**	**Date of Outcome**<br>Day Month Year	**Relationship to study drug**
**1.**					
**2.**					
**3.**					
**4.**					
	1 = mild<br>2 = moderate<br>3 = severe		1 = No<br>2 = Yes		1 = Definite<br>2 = Probable<br>3 = Possible<br>4 = Not related

If hospitalised: Date of admission (Day Month Year) Date of Discharge (Day Month Year)

FURTHER DETAILS OF THE SAE(S) AND COMMENTS:
Include details of signs/symptoms, tests and investigations, treatment given and event progression.
Send additional documentation if available e.g. lab reports, post mortem etc.

HISTORY RELEVANT TO THE SAE: medical, social, family and drug history etc.

TIMING OF THE SAE: (When did the SAE occur?)

☐ Before treatment with study medication ☐ During treatment ☐ During follow-up (after stopping study medication)

Trial No. GERP ALS	Screening No.	Randomisation No.		Investigator:

MEDICATION: DRUGS BEING TAKEN AT TIME OF THE SAE

Study drug name	Indication	Dosage	From (Day Month Year)	To (Day Month Year)	Ongoing
______ If treatment is blinded, please use the term "blind"	______	______	|_|_|_|_|_|_|	|_|_|_|_|_|_|	☐
______ If treatment is blinded, please use the term "blind"	______	______	|_|_|_|_|_|_|	|_|_|_|_|_|_|	☐
______ If treatment is blinded, please use the term "blind"	______	______	|_|_|_|_|_|_|	|_|_|_|_|_|_|	☐

OTHER DRUGS: give generic name only – please do *not* include drugs used to treat the events

Generic drug name	Indication	Dosage	From: Day Month Year	To: Day Month Year	Ongoing
1.______	______	______			☐
2.______	______	______			☐
3.______	______	______			☐
4.______	______	______			☐
5.______	______	______			☐
6.______	______	______			☐

STUDY MEDICATION – ACTION TAKEN:

	No	Yes	N/A
Study medication continued unchanged?	☐	☐	☐
Study medication discontinued?	☐	☐	☐
Did the SAE abate after dose reduction/ interruption/ discontinuation?	☐	☐	☐
Did the SAE re-appear after reintroduction?	☐	☐	☐
Did the SAE re-appear after the dosage was increased again?	☐	☐	☐

	No	Yes	N/A
Study medication dose reduced? From: Day Month Year To: Day Month Year	☐	☐	☐
Study medication interrupted? From: Day Month Year To: Day Month Year	☐	☐	☐

TREATMENT OTHER THAN CHANGES TO STUDY MEDICATION

Was treatment given for the SAE(s)? ☐ **No** ☐ **Yes** If yes – please specify drugs(s), dosages and date(s):

______

______

Please specify below any special measures, other than medication (e.g. hospitalisation, surgery, ECG monitoring etc.) taken for the SAE:

______

______

Trial No. GERP ALS	Screening No.	Randomisation No.		Investigator:

OVERALL OUTCOME (TO DATE):

- ☐ Recovered → Date: |_|_|_|_|_|_|
- ☐ Recovered with persistent damage → Date: |_|_|_|_|_|_| Damage: ____________
- ☐ Not yet recovered ____________
- ☐ Not known
- ☐ Patient died → Date: |_|_|_|_|_|_|

Date Signature of person completing the form

Date Signature of Trial investigator

APPENDIX VI Declaration of Helsinki

Initiated: 1964 17.C
Original: English

WORLD MEDICAL ASSOCIATION DECLARATION OF HELSINKI
Ethical Principles
for
Medical Research Involving Human Subjects

Adopted by the 18th WMA General Assembly
Helsinki, Finland, June 1964
and amended by the
29th WMA General Assembly, Tokyo, Japan, October 1975
35th WMA General Assembly, Venice, Italy, October 1983
41st WMA General Assembly, Hong Kong, September 1989
48th WMA General Assembly, Somerset West, Republic of South Africa, October 1996
and the
52nd WMA General Assembly, Edinburgh, Scotland, October 2000
Note of Clarification on Paragraph 29 added by the WMA General Assembly, Washington 2002

A. INTRODUCTION

1. The World Medical Association has developed the Declaration of Helsinki as a statement of ethical principles to provide guidance to physicians and other participants in medical research involving human subjects**.** Medical research involving human subjects includes research on identifiable human material or identifiable data.
2. It is the duty of the physician to promote and safeguard the health of the people. The physician's knowledge and conscience are dedicated to the fulfillment of this duty.
3. The Declaration of Geneva of the World Medical Association binds the physician with the words, "The health of my patient will be my first consideration," and the International Code of Medical Ethics declares that, "A physician shall act only in thepatient's interest when providing medical care which might have the effect of weakening the physical and mental condition of the patient."
4. Medical progress is based on research which ultimately must rest in part on experimentation involving human subjects.
5. In medical research on human subjects, considerations related to the well-being of the human subject should take precedence over the interests of science and society.
6. The primary purpose of medical research involving human subjects is to improve prophylactic, diagnostic and therapeutic procedures and the understanding of the aetiology and pathogenesis of disease. Even the best proven prophylactic, diagnostic, and therapeutic methods must continuously be challenged through research for their effectiveness, efficiency, accessibility and quality.
7. In current medical practice and in medical research, most prophylactic, diagnostic and therapeutic procedures involve risks and burdens.
8. Medical research is subject to ethical standards that promote respect for all human beings and protect their health and rights. Some research populations are vulnerable and need special protection. The particular needs of the economically and medically disadvantaged must be recognized. Special attention is also required for those who cannot give or refuse consent for themselves, for those who may be subject to giving consent under duress, for those who will not benefit personally from the research and for those for whom the research is combined with care.
9. Research Investigators should be aware of the ethical, legal and regulatory requirements for research on human subjects in their own countries as well as applicable international requirements. No national ethical, legal or regulatory requirement should be allowed to reduce or eliminate any of the protections for human subjects set forth in this Declaration.

17.C
The Declaration of Helsinki (Document 17.C) is an official policy document of the World Medical Association, the global representative body for physicians. It was first adopted in 1964 (Helsinki, Finland) and revised in 1975 (Tokyo, Japan), 1983 (Venice, Italy), 1989 (Hong Kong), 1996 (Somerset-West, South Africa) and 2000 (Edinburgh, Scotland). Note of clarification on Paragraph 29 added by the WMA General Assembly, Washington 2002.
2

B. BASIC PRINCIPLES FOR ALL MEDICAL RESEARCH

10. It is the duty of the physician in medical research to protect the life, health, privacy, and dignity of the human subject.
11. Medical research involving human subjects must conform to generally accepted scientific principles, be based on a thorough knowledge of the scientific literature, other relevant sources of information, and on adequate laboratory and, where appropriate, animal experimentation.
12. Appropriate caution must be exercised in the conduct of research which may affect the environment, and the welfare of animals used for research must be respected.
13. The design and performance of each experimental procedure involving human subjects should be clearly formulated in an experimental protocol. This protocol should be submitted for consideration, comment, guidance, and where appropriate, approval to a specially appointed ethical review committee, which must be independent of the investigator, the sponsor or any other kind of undue influence. This independent committee should be in conformity with the laws and regulations of the country in which the research experiment is performed. The committee has the right to monitor ongoing trials. The researcher has the obligation to provide monitoring information to the committee, especially any serious adverse events. The researcher should also submit to the committee, for review, information regarding funding, sponsors, institutional affiliations, other potential conflicts of interest and incentives for subjects.
14. The research protocol should always contain a statement of the ethical considerations involved and should indicate that there is compliance with the principles enunciated in this Declaration.
15. Medical research involving human subjects should be conducted only by scientifically qualified persons and under the supervision of a clinically competent medical person. The responsibility for the human subject must always rest with a medically qualified person and never rest on the subject of the research, even though the subject has given consent.
16. Every medical research project involving human subjects should be preceded by careful assessment of predictable risks and burdens in comparison with foreseeable benefits to the subject or to others. This does not preclude the participation of healthy volunteers in medical research. The design of all studies should be publicly available.
17. Physicians should abstain from engaging in research projects involving human subjects unless they are confident that the risks involved have been adequately assessed and can be satisfactorily managed. Physicians should cease any investigation if the risks are found to outweigh the potential benefits or if there is conclusive proof of positive and beneficial results.
18. Medical research involving human subjects should only be conducted if the importance of the objective outweighs the inherent risks and burdens to the subject. This is especially important when the human subjects are healthy volunteers.
19. Medical research is only justified if there is a reasonable likelihood that the populations in which the research is carried out stand to benefit from the results of the research.
20. The subjects must be volunteers and informed participants in the research project.

17.C
The Declaration of Helsinki (Document 17.C) is an official policy document of the World Medical Association, the global representative body for physicians. It was first adopted in 1964 (Helsinki, Finland) and revised in 1975 (Tokyo, Japan), 1983 (Venice, Italy), 1989 (Hong Kong), 1996 (Somerset-West, South Africa) and 2000 (Edinburgh, Scotland). Note of clarification on Paragraph 29 added by the WMA General Assembly, Washington 2002.
3

21. The right of research subjects to safeguard their integrity must always be respected. Every precaution should be taken to respect the privacy of the subject, the confidentiality of the patient's information and to minimize the impact of the study on the subject's physical and mental integrity and on the personality of the subject.
22. In any research on human beings, each potential subject must be adequately informed of the aims, methods, sources of funding, any possible conflicts of interest, institutional affiliations of the researcher, the anticipated benefits and potential risks of the study and the discomfort it may entail. The subject should be informed of the right to abstain from participation in the study or to withdraw consent to participate at any time without reprisal. After ensuring that the subject has understood the information, the physician should then obtain the subject's freely-given informed consent, preferably in writing. If the consent cannot be obtained in writing, the non-written consent must be formally documented and witnessed.
23. When obtaining informed consent for the research project the physician should be particularly cautious if the subject is in a dependent relationship with the physician or may consent under duress. In that case the informed consent should be obtained by a well-informed physician who is not engaged in the investigation and who is completely independent of this relationship.
24. For a research subject who is legally incompetent, physically or mentally incapable of giving consent or is a legally incompetent minor, the investigator must obtain informed consent from the legally authorized representative in accordance with applicable law. These groups should not be included in research unless the research is necessary to promote the health of the population represented and this research cannot instead be performed on legally competent persons.
25. When a subject deemed legally incompetent, such as a minor child, is able to give assent to decisions about participation in research, the investigator must obtain that assent in addition to the consent of the legally authorized representative.
26. Research on individuals from whom it is not possible to obtain consent, including proxy or advance consent, should be done only if the physical/mental condition that prevents obtaining informed consent is a necessary characteristic of the research population. The specific reasons for involving research subjects with a condition that renders them unable to give informed consent should be stated in the experimental protocol for consideration and approval of the review committee. The protocol should state that consent to remain in the research should be obtained as soon as possible from the individual or a legally authorized surrogate.
27. Both authors and publishers have ethical obligations. In publication of the results of research, the investigators are obliged to preserve the accuracy of the results. Negative as well as positive results should be published or otherwise publicly available. Sources of funding, institutional affiliations and any possible conflicts of interest should be declared in the publication. Reports of experimentation not in accordance with the principles laid down in this Declaration should not be accepted for publication.

17.C
The Declaration of Helsinki (Document 17.C) is an official policy document of the World Medical Association, the global representative body for physicians. It was first adopted in 1964 (Helsinki, Finland) and revised in 1975 (Tokyo, Japan), 1983 (Venice, Italy), 1989 (Hong Kong), 1996 (Somerset-West, South Africa) and 2000 (Edinburgh, Scotland). Note of clarification on Paragraph 29 added by the WMA General Assembly, Washington 2002.
4

C. ADDITIONAL PRINCIPLES FOR MEDICAL RESEARCH COMBINED WITH MEDICAL CARE

28. The physician may combine medical research with medical care, only to the extent that the research is justified by its potential prophylactic, diagnostic or therapeutic value. When medical research is combined with medical care, additional standards apply to protect the patients who are research subject.
29. The benefits, risks, burdens and effectiveness of a new method should be tested against those of the best current prophylactic, diagnostic, and therapeutic methods. This does not exclude the use of placebo, or no treatment, in studies where no proven prophylactic, diagnostic or therapeutic method exists. *(See footnote*)*
30. At the conclusion of the study, every patient entered into the study should be assured of access to the best proven prophylactic, diagnostic and therapeutic methods identified by the study.
31. The physician should fully inform the patient which aspects of the care are related to the research. The refusal of a patient to participate in a study must never interfere with the patient-physician relationship.
32. In the treatment of a patient, where proven prophylactic, diagnostic and therapeutic methods do not exist or have been ineffective, the physician, with informed consent from the patient, must be free to use unproven or new prophylactic, diagnostic and therapeutic measures, if in the physician’s judgement it offers hope of saving life, re-establishing health or alleviating suffering. Where possible, these measures should be made the object of research, designed to evaluate their safety and efficacy. In all cases, new information should be recorded and, where appropriate, published. The other relevant guidelines of this Declaration should be followed.

FOOTNOTE:

Note of Clarification on Paragraph 29 of the WMA Declaration of Helsinki

The WMA hereby reaffirms its position that extreme care must be taken in making use of a placebo-controlled trial and that in general this methodology should only be used in the absence of existing proven therapy. However, a placebo-controlled trial may be ethically acceptable, even if proven therapy is available, under the following circumstances:

- Where for compelling and scientifically sound methodological reasons its use is necessary to determine the efficacy or safety of a prophylactic, diagnostic or therapeutic method; or
- Where a prophylactic, diagnostic or therapeutic method is being investigated for a minor condition and the patients who receive placebo will not be subject to any additional risk of serious or irreversible harm.

All other provisions of the Declaration of Helsinki must be adhered to, especially the need for appropriate ethical and scientific review.

♣ ♣ ♣
6.10.2002

APPENDIX VII Extract from German Drug Law (AMG §§ 40 - 42)

Auszug aus dem Arzneimittelgesetz:

GESETZ ÜBER DEN VERKEHR MIT ARZNEIMITTELN
ARZNEIMITTELGESETZ (AMG)

Stand: 12. Gesetz zur Änderung des Arzneimittelgesetzes vom 30. Juli 2004
BGBl I 2004 vom 5. August 2004, 2031-2053

§ 40
Allgemeine Voraussetzungen der klinischen Prüfung

(1) Der Sponsor, der Prüfer und alle weiteren an der klinischen Prüfung beteiligten Personen haben bei der Durchführung der klinischen Prüfung eines Arzneimittels bei Menschen die Anforderungen der guten klinischen Praxis nach Maßgabe des Artikels 1 Abs. 3 der Richtlinie 2001/20/EG des Europäischen Parlaments und des Rates vom 4. April 2001 zur Angleichung der Rechts- und Verwaltungsvorschriften der Mitgliedstaaten über die Anwendung der guten klinischen Praxis bei der Durchführung von klinischen Prüfungen mit Humanarzneimitteln (ABl. EG Nr. L 121 S. 34) einzuhalten. Die klinische Prüfung eines Arzneimittels bei Menschen darf vom Sponsor nur begonnen werden, wenn die zuständige Ethik-Kommission diese nach Maßgabe des § 42 Abs. 1 zustimmend bewertet und die zuständige Bundesoberbehörde diese nach Maßgabe des § 42 Abs. 2 genehmigt hat. Die klinische Prüfung eines Arzneimittels darf bei Menschen nur durchgeführt werden, wenn und solange

1. ein Sponsor oder ein Vertreter des Sponsors vorhanden ist, der seinen Sitz in einem Mitgliedstaat der Europäischen Union oder in einem anderen Vertragsstaat des Abkommens über den Europäischen Wirtschaftsraum hat,
2. die vorhersehbaren Risiken und Nachteile gegenüber dem Nutzen für die Person, bei der sie durchgeführt werden soll (betroffene Person), und der voraussichtlichen Bedeutung des Arzneimittels für die Heilkunde ärztlich vertretbar sind,
3. die betroffene Person
   a) volljährig und in der Lage ist, Wesen, Bedeutung und Tragweite der klinischen Prüfung zu erkennen und ihren Willen hiernach auszurichten,
   b) nach Absatz 2 Satz 1 aufgeklärt worden ist und schriftlich eingewilligt hat, soweit in Absatz 4 oder in § 41 nichts Abweichendes bestimmt ist und
   c) nach Absatz 2a Satz 1 und 2 informiert worden ist und schriftlich eingewilligt hat; die Einwilligung muss sich ausdrücklich auch auf die Erhebung und Verarbeitung von Angaben über die Gesundheit beziehen,
4. die betroffene Person nicht auf gerichtliche oder behördliche Anordnung in einer Anstalt untergebracht ist,
5. sie in einer geeigneten Einrichtung von einem angemessen qualifizierten Prüfer verantwortlich durchgeführt wird und die Leitung von einem Prüfer, Hauptprüfer oder Leiter der klinischen Prüfung wahrgenommen wird, der eine mindestens zweijährige Erfahrung in der klinischen Prüfung von Arzneimitteln nachweisen kann,
6. eine dem jeweiligen Stand der wissenschaftlichen Erkenntnisse entsprechende pharmakologisch-toxikologische Prüfung des Arzneimittels durchgeführt worden ist,
7. jeder Prüfer durch einen für die pharmakologisch-toxikologische Prüfung verantwortlichen Wissenschaftler über deren Ergebnisse und die voraussichtlich mit der klinischen Prüfung verbundenen Risiken informiert worden ist,
8. für den Fall, dass bei der Durchführung der klinischen Prüfung ein Mensch getötet oder der Körper oder die Gesundheit eines Menschen verletzt wird, eine Versicherung nach Maßgabe des Absatzes 3 besteht, die auch Leistungen gewährt, wenn kein anderer für den Schaden haftet, und
9. für die medizinische Versorgung der betroffenen Person ein Arzt oder bei zahnmedizinischer Behandlung ein Zahnarzt verantwortlich ist.

(2) Die betroffene Person ist durch einen Prüfer, der Arzt oder bei zahnmedizinischer Prüfung Zahnarzt ist, über Wesen, Bedeutung, Risiken und Tragweite der klinischen Prüfung sowie über ihr Recht aufzuklären, die Teilnahme an der klinischen Prüfung jederzeit zu beenden; ihr ist eine allgemein verständliche Aufklärungsunterlage auszuhändigen. Der betroffenen Person ist ferner Gelegenheit zu einem Beratungsgespräch mit einem Prüfer über die sonstigen Bedingungen der Durchführung der klinischen Prüfung zu geben. Eine nach Absatz 1 Satz 3 Nr. 3 Buchstabe b erklärte Einwilligung in die Teilnahme an einer klinischen Prüfung kann jederzeit gegenüber dem Prüfer schriftlich oder mündlich widerrufen werden, ohne dass der betroffenen Person dadurch Nachteile entstehen dürfen.

(2a) Die betroffene Person ist über Zweck und Umfang der Erhebung und Verwendung personenbezogener Daten, insbesondere von Gesundheitsdaten zu informieren. Sie ist insbesondere darüber zu informieren, dass

1. die erhobenen Daten soweit erforderlich
   a) zur Einsichtnahme durch die Überwachungsbehörde oder Beauftragte des Sponsors zur Überprüfung der ordnungsgemäßen Durchführung der klinischen Prüfung bereitgehalten werden,
   b) pseudonymisiert an den Sponsor oder eine von diesem beauftragte Stelle zum Zwecke der wissenschaftlichen Auswertung weitergegeben werden,
   c) im Falle eines Antrags auf Zulassung pseudonymisiert an den Antragsteller und die für die Zulassung zuständige Behörde weitergegeben werden,
   d) im Falle unerwünschter Ereignisse des zu prüfenden Arzneimittels pseudonymisiert an den Sponsor und die zuständige Bundesoberbehörde sowie von dieser an die Europäische Datenbank weitergegeben werden,
2. die Einwilligung nach Absatz 1 Satz 3 Nr. 3 Buchstabe c unwiderruflich ist,
3. im Falle eines Widerrufs der nach Absatz 1 Satz 3 Nr. 3 Buchstabe b erklärten Einwilligung die gespeicherten Daten weiterhin verwendet werden dürfen, soweit dies erforderlich ist, um
   a) Wirkungen des zu prüfenden Arzneimittels festzustellen,
   b) sicherzustellen, dass schutzwürdige Interessen der betroffenen Person nicht beeinträchtigt werden,
   c) der Pflicht zur Vorlage vollständiger Zulassungsunterlagen zu genügen,
4. die Daten bei den genannten Stellen für die auf Grund des § 42 Abs. 3 bestimmten Fristen gespeichert werden.

Im Falle eines Widerrufs der nach Absatz 1 Satz 3 Nr. 3 Buchstabe b erklärten Einwilligung haben die verantwortlichen Stellen unverzüglich zu prüfen, inwieweit die gespeicherten Daten für die in Satz 1 Nr. 3 genannten Zwecke noch erforderlich sein können. Nicht mehr benötigte Daten sind unverzüglich zu löschen. Im Übrigen sind die erhobenen personenbezogenen Daten nach Ablauf der auf Grund des § 42 Abs. 3 bestimmten Fristen zu löschen, soweit nicht gesetzliche, satzungsmäßige oder vertragliche Aufbewahrungsfristen entgegenstehen.

(3) Die Versicherung nach Absatz 1 Satz 3 Nr. 8 muss zugunsten der von der klinischen Prüfung betroffenen Personen bei einem in einem Mitgliedstaat der Europäischen Union oder einem anderen Vertragsstaat des Abkommens über den Europäischen Wirtschaftsraum zum Geschäftsbetrieb zugelassenen Versicherer genommen werden. Ihr Umfang muss in einem angemessenen Verhältnis zu den mit der klinischen Prüfung verbundenen Risiken stehen und auf der Grundlage der Risikoabschätzung so festgelegt werden, dass für jeden Fall des Todes oder der dauernden Erwerbsunfähigkeit einer von der klinischen Prüfung betroffenen Person mindestens 500.000 Euro zur Verfügung stehen. Soweit aus der Versicherung geleistet wird, erlischt ein Anspruch auf Schadensersatz.

(4) Auf eine klinische Prüfung bei Minderjährigen finden die Absätze 1 bis 3 mit folgender Maßgabe Anwendung:

1. Das Arzneimittel muss zum Erkennen oder zum Verhüten von Krankheiten bei Minderjährigen bestimmt und die Anwendung des Arzneimittels nach den Erkenntnissen der medizinischen Wissenschaft angezeigt sein, um bei dem Minderjährigen Krankheiten zu erkennen oder ihn vor Krankheiten zu schützen. Angezeigt ist das Arzneimittel, wenn seine Anwendung bei dem Minderjährigen medizinisch indiziert ist.

2. Die klinische Prüfung an Erwachsenen oder andere Forschungsmethoden dürfen nach den Erkenntnissen der medizinischen Wissenschaft keine ausreichenden Prüfergebnisse erwarten lassen.

3. Die Einwilligung wird durch den gesetzlichen Vertreter abgegeben, nachdem er entsprechend Absatz 2 aufgeklärt worden ist. Sie muss dem mutmaßlichen Willen des Minderjährigen entsprechen, soweit ein solcher feststellbar ist. Der Minderjährige ist vor Beginn der klinischen Prüfung von einem im Umgang mit Minderjährigen erfahrenen Prüfer über die Prüfung, die Risiken und den Nutzen aufzuklären, soweit dies im Hinblick auf sein Alter und seine geistige Reife möglich ist; erklärt der Minderjährige, nicht an der klinischen Prüfung teilnehmen zu wollen, oder bringt er dies in sonstiger Weise zum Ausdruck, so ist dies zu beachten. Ist der Minderjährige in der Lage, Wesen, Bedeutung und Tragweite der klinischen Prüfung zu erkennen und seinen Willen hiernach auszurichten, so ist auch seine Einwilligung erforderlich. Eine Gelegenheit zu einem Beratungsgespräch nach Absatz 2 Satz 2 ist neben dem gesetzlichen Vertreter auch dem Minderjährigen zu eröffnen.

4. Die klinische Prüfung darf nur durchgeführt werden, wenn sie für die betroffene Person mit möglichst wenig Belastungen und anderen vorhersehbaren Risiken verbunden ist; sowohl der Belastungsgrad als auch die Risikoschwelle müssen im Prüfplan eigens definiert und vom Prüfer ständig überprüft werden.

5. Vorteile mit Ausnahme einer angemessenen Entschädigung dürfen nicht gewährt werden.

(5) Der betroffenen Person, ihrem gesetzlichen Vertreter oder einem von ihr Bevollmächtigten steht eine zuständige Kontaktstelle zur Verfügung, bei der Informationen über alle Umstände, denen eine Bedeutung für die Durchführung einer klinischen Prüfung beizumessen ist, eingeholt werden können. Die Kontaktstelle ist bei der jeweils zuständigen Bundesoberbehörde einzurichten.

§ 41
Besondere Voraussetzungen

(1) Auf eine klinische Prüfung bei einer volljährigen Person, die an einer Krankheit leidet, zu deren Behandlung das zu prüfende Arzneimittel angewendet werden soll, findet § 40 Abs. 1 bis 3 mit folgender Maßgabe Anwendung:

1. Die Anwendung des zu prüfenden Arzneimittels muss nach den Erkenntnissen der medizinischen Wissenschaft angezeigt sein, um das Leben dieser Person zu retten, ihre Gesundheit wiederherzustellen oder ihr Leiden zu erleichtern, oder
2. sie muss für die Gruppe der Patienten, die an der gleichen Krankheit leiden wie diese Person, mit einem direkten Nutzen verbunden sein.

Kann die Einwilligung wegen einer Notfallsituation nicht eingeholt werden, so darf eine Behandlung, die ohne Aufschub erforderlich ist, um das Leben der betroffenen Person zu retten, ihre Gesundheit wiederherzustellen oder ihr Leiden zu erleichtern, umgehend erfolgen. Die Einwilligung zur weiteren Teilnahme ist einzuholen, sobald dies möglich und zumutbar ist.

(2) Auf eine klinische Prüfung bei einem Minderjährigen, der an einer Krankheit leidet, zu deren Behandlung das zu prüfende Arzneimittel angewendet werden soll, findet § 40 Abs. 1 bis 4 mit folgender Maßgabe Anwendung:

1. Die Anwendung des zu prüfenden Arzneimittels muss nach den Erkenntnissen der medizinischen Wissenschaft angezeigt sein, um das Leben der betroffenen Person zu retten, ihre Gesundheit wiederherzustellen oder ihr Leiden zu erleichtern, oder
2. a) die klinische Prüfung muss für die Gruppe der Patienten, die an der gleichen Krankheit leiden wie die betroffene Person, mit einem direkten Nutzen verbunden sein,

b) die Forschung muss für die Bestätigung von Daten, die bei klinischen Prüfungen an anderen Personen oder mittels anderer Forschungsmethoden gewonnen wurden, unbedingt erforderlich sein,
c) die Forschung muss sich auf einen klinischen Zustand beziehen, unter dem der betroffene Minderjährige leidet und
d) die Forschung darf für die betroffene Person nur mit einem minimalen Risiko und einer minimalen Belastung verbunden sein; die Forschung weist nur ein minimales Risiko auf, wenn nach Art und Umfang der Intervention zu erwarten ist, dass sie allenfalls zu einer sehr geringfügigen und vorübergehenden Beeinträchtigung der Gesundheit der betroffenen Person führen wird; sie weist eine minimale Belastung auf, wenn zu erwarten ist, dass die Unannehmlich keiten für die betroffene Person allenfalls vorübergehend auftreten und sehr geringfügig sein werden.

Satz 1 Nr. 2 gilt nicht für Minderjährige, für die nach Erreichen der Volljährigkeit Absatz 3 Anwendung finden würde.

(3) Auf eine klinische Prüfung bei einer volljährigen Person, die nicht in der Lage ist, Wesen, Bedeutung und Tragweite der klinischen Prüfung zu erkennen und ihren Willen hiernach auszurichten und die an einer Krankheit leidet, zu deren Behandlung das zu prüfende Arzneimittel angewendet werden soll, findet § 40 Abs. 1 bis 3 mit folgender Maßgabe Anwendung:

1. Die Anwendung des zu prüfenden Arzneimittels muss nach den Erkenntnissen der medizinischen Wissenschaft angezeigt sein, um das Leben der betroffenen Person zu retten, ihre Gesundheit wiederherzustellen oder ihr Leiden zu erleichtern; außerdem müssen sich derartige Forschungen unmittelbar auf einen lebensbedrohlichen oder sehr geschwächten klinischen Zustand beziehen, in dem sich die betroffene Person befindet, und die klinische Prüfung muss für die betroffene Person mit möglichst wenig Belastungen und anderen vorhersehbaren Risiken verbunden sein; sowohl der Belastungsgrad als auch die Risikoschwelle müssen im Prüfplan eigens definiert und vom Prüfer ständig überprüft werden. Die klinische Prüfung darf nur durchgeführt werden, wenn die begründete Erwartung besteht, dass der Nutzen der Anwendung des Prüfpräparates für die betroffene Person die Risiken überwiegt oder keine Risiken mit sich bringt.

2. Die Einwilligung wird durch den gesetzlichen Vertreter oder Bevollmächtigten abgegeben, nachdem er entsprechend § 40 Abs. 2 aufgeklärt worden ist. § 40 Abs. 4 Nr. 3 Satz 2, 3 und 5 gilt entsprechend.

3. Die Forschung muss für die Bestätigung von Daten, die bei klinischen Prüfungen an zur Einwilligung nach Aufklärung fähigen Personen oder mittels anderer Forschungsmethoden gewonnen wurden, unbedingt erforderlich sein. § 40 Abs. 4 Nr. 2 gilt entsprechend.

4. Vorteile mit Ausnahme einer angemessenen Entschädigung dürfen nicht gewährt werden.

§ 42
Verfahren bei der Ethik-Kommission, Genehmigungsverfahren bei der Bundesoberbehörde

(1) Die nach § 40 Abs. 1 Satz 2 erforderliche zustimmende Bewertung der Ethik-Kommission ist vom Sponsor bei der nach Landesrecht für den Prüfer zuständigen unabhängigen interdisziplinär besetzten Ethik-Kommission zu beantragen. Wird die klinische Prüfung von mehreren Prüfern durchgeführt, so ist der Antrag bei der für den Hauptprüfer oder Leiter der klinischen Prüfung zuständigen unabhängigen Ethik-Kommission zu stellen. Das Nähere zur Bildung, Zusammensetzung und Finanzierung der Ethik-Kommission wird durch Landesrecht bestimmt. Der Sponsor hat der Ethik-Kommission alle Angaben und Unterlagen vorzulegen, die diese zur Bewertung benötigt. Zur Bewertung der Unterlagen kann die Ethik-Kommission eigene wissenschaftliche Erkenntnisse verwerten, Sachverständige beiziehen oder Gutachten

anfordern. Sie hat Sachverständige beizuziehen oder Gutachten anzufordern, wenn es sich um eine klinische Prüfung bei Minderjährigen handelt und sie nicht über eigene Fachkenntnisse auf dem Gebiet der Kinderheilkunde, einschließlich ethischer und psychosozialer Fragen der Kinderheilkunde, verfügt oder wenn es sich um eine klinische Prüfung von xenogenen Zelltherapeutika oder Gentransfer-Arzneimitteln handelt. Die zustimmende Bewertung darf nur versagt werden, wenn

1. die vorgelegten Unterlagen auch nach Ablauf einer dem Sponsor gesetzten angemessenen Frist zur Ergänzung unvollständig sind,
2. die vorgelegten Unterlagen einschließlich des Prüfplans, der Prüferinformation und der Modalitäten für die Auswahl der Prüfungsteilnehmer nicht dem Stand der wissenschaftlichen Erkenntnisse entsprechen, insbesondere die klinische Prüfung ungeeignet ist, den Nachweis der Unbedenklichkeit oder Wirksamkeit eines Arzneimittels einschließlich einer unterschiedlichen Wirkungsweise bei Frauen und Männern zu erbringen, oder
3. die in § 40 Abs. 1 Satz 3 Nr. 2 bis 9, Abs. 4 und § 41 geregelten Anforderungen nicht erfüllt sind.

Das Nähere wird in der Rechtsverordnung nach Absatz 3 bestimmt. Die Ethik-Kommission hat eine Entscheidung über den Antrag nach Satz 1 innerhalb einer Frist von höchstens 60 Tagen nach Eingang der erforderlichen Unterlagen zu übermitteln, die nach Maßgabe der Rechtsverordnung nach Absatz 3 verlängert oder verkürzt werden kann; für die Prüfung xenogener Zelltherapeutika gibt es keine zeitliche Begrenzung für den Genehmigungszeitraum.

(2) Die nach § 40 Abs. 1 Satz 2 erforderliche Genehmigung der zuständigen Bundesoberbehörde ist vom Sponsor bei der zuständigen Bundesoberbehörde zu beantragen. Der Sponsor hat dabei alle Angaben und Unterlagen vorzulegen, die diese zur Bewertung benötigt, insbesondere die Ergebnisse der analytischen und der pharmakologisch-toxikologischen Prüfung sowie den Prüfplan und die klinischen Angaben zum Arzneimittel einschließlich der Prüferinformation. Die Genehmigung darf nur versagt werden, wenn

1. die vorgelegten Unterlagen auch nach Ablauf einer dem Sponsor gesetzten angemessenen Frist zur Ergänzung unvollständig sind,
2. die vorgelegten Unterlagen, insbesondere die Angaben zum Arzneimittel und der Prüfplan einschließlich der Prüferinformation nicht dem Stand der wissenschaftlichen Erkenntnisse entsprechen, insbesondere die klinische Prüfung ungeeignet ist, den Nachweis der Unbedenklichkeit oder Wirksamkeit eines Arzneimittels einschließlich einer unterschiedlichen Wirkungsweise bei Frauen und Männern zu erbringen, oder
3. die in § 40 Abs. 1 Satz 3 Nr. 1, 2 und 6, bei xenogenen Zelltherapeutika auch die in Nummer 8 geregelten Anforderungen insbesondere im Hinblick auf eine Versicherung von Drittrisiken nicht erfüllt sind.

Die Genehmigung gilt als erteilt, wenn die zuständige Bundesoberbehörde dem Sponsor innerhalb von höchstens 30 Tagen nach Eingang der Antragsunterlagen keine mit Gründen versehenen Einwände übermittelt. Wenn der Sponsor auf mit Gründen versehene Einwände den Antrag nicht innerhalb einer Frist von höchstens 90 Tagen entsprechend abgeändert hat, gilt der Antrag als abgelehnt. Das Nähere wird in der Rechtsverordnung nach Absatz 3 bestimmt. Abweichend von Satz 4 darf die klinische Prüfung von Arzneimitteln,

1. die unter Teil A des Anhangs der Verordnung (EWG) Nr. 2309/93 fallen,
2. die somatische Zelltherapeutika, xenogene Zelltherapeutika, Gentransfer-Arzneimittel sind,
3. die genetisch veränderte Organismen enthalten oder
4. deren Wirkstoff ein biologisches Produkt menschlichen oder tierischen Ursprungs ist oder biologische Bestandteile menschlichen oder tierischen Ursprungs enthält oder zu seiner Herstellung derartige Bestandteile erfordert,

nur begonnen werden, wenn die zuständige Bundesoberbehörde dem Sponsor eine schriftliche Genehmigung erteilt hat. Die zuständige Bundesoberbehörde hat eine Entscheidung über den Antrag auf Genehmigung von Arzneimitteln nach Satz 7 Nr. 2 bis 4 innerhalb einer Frist von höchstens 60 Tagen nach Eingang der in Satz 2 genannten erforderlichen Unterlagen zu treffen, die nach Maßgabe einer Rechtsverordnung nach Absatz 3 verlängert oder verkürzt werden kann; für die Prüfung xenogener Zelltherapeutika gibt es keine zeitliche Begrenzung für den Genehmigungszeitraum.

(2a) Die für die Genehmigung einer klinischen Prüfung nach Absatz 2 zuständige Bundesoberbehörde unterrichtet die nach Absatz 1 zuständige Ethik-Kommission, sofern ihr Informationen zu anderen klinischen Prüfungen vorliegen, die für die Bewertung der von der Ethik-Kommission begutachteten Prüfung von Bedeutung sind; dies gilt insbesondere für Informationen über abgebrochene oder sonst vorzeitig beendete Prüfungen. Dabei unterbleibt die Übermittlung personenbezogener Daten, ferner sind Betriebs- und Geschäftsgeheimnisse dabei zu wahren.

(3) Das Bundesministerium wird ermächtigt, durch Rechtsverordnung mit Zustimmung des Bundesrates Regelungen zur Gewährleistung der ordnungsgemäßen Durchführung der klinischen Prüfung und der Erzielung dem wissenschaftlichen Erkenntnisstand entsprechender Unterlagen zu treffen. In der Rechtsverordnung können insbesondere Regelungen getroffen werden über:

1. ferner kann die Weiterleitung von Unterlagen und Ausfertigungen der Entscheidungen an die Aufgaben und Verantwortungsbereiche des Sponsors, der Prüfer oder anderer Personen, die die klinische Prüfung durchführen oder kontrollieren einschließlich von Anzeige-, Dokumentations- und Berichtspflichten insbesondere über Nebenwirkungen und sonstige unerwünschte Ereignisse, die während der Studie auftreten und die Sicherheit der Studienteilnehmer oder die Durchführung der Studie beeinträchtigen könnten,
2. die Aufgaben der und das Verfahren bei Ethik-Kommissionen einschließlich der einzureichenden Unterlagen, auch mit Angaben zur angemessenen Beteiligung von Frauen und Männern als Prüfungsteilnehmerinnen und Prüfungsteilnehmer, der Unterbrechung oder Verlängerung oder Verkürzung der Bearbeitungsfrist und der besonderen Anforderungen an die Ethik-Kommissionen bei klinischen Prüfungen nach § 40 Abs. 4 und § 41 Abs. 2 und 3,
3. die Aufgaben der zuständigen Behörden und das behördliche Genehmigungsverfahren einschließlich der einzureichenden Unterlagen, auch mit Angaben zur angemessenen Beteiligung von Frauen und Männern als Prüfungsteilnehmerinnen und Prüfungsteilnehmer, und der Unterbrechung oder Verlängerung oder Verkürzung der Bearbeitungsfrist, das Verfahren zur Überprüfung von Unterlagen in Betrieben und Einrichtungen sowie die Voraussetzungen und das Verfahren für Rücknahme, Widerruf und Ruhen der Genehmigung oder Untersagung einer klinischen Prüfung,
4. die Anforderungen an das Führen und Aufbewahren von Nachweisen,
5. die Übermittlung von Namen und Sitz des Sponsors und des verantwortlichen Prüfers und nicht personenbezogener Angaben zur klinischen Prüfung von der zuständigen Behörde an eine europäische Datenbank und
6. die Befugnisse zur Erhebung und Verwendung personenbezogener Daten, soweit diese für die Durchführung und Überwachung der klinischen Prüfung erforderlich sind; dies gilt auch für die Verarbeitung von Daten, die nicht in Dateien verarbeitet oder genutzt werden;

die zuständigen Behörden und die für die Prüfer zuständigen Ethik-Kommissionen bestimmt sowie vorgeschrieben werden, dass Unterlagen in mehrfacher Ausfertigung sowie auf elektronischen oder optischen Speichermedien eingereicht werden. In der Rechtsverordnung sind für zugelassene Arzneimittel Ausnahmen entsprechend der Richtlinie 2001/20/EG vorzusehen.

§ 42a
Rücknahme, Widerruf und Ruhen der Genehmigung

(1) Die Genehmigung ist zurückzunehmen, wenn bekannt wird, dass ein Versagungsgrund nach § 42 Abs. 2 Satz 3 Nr. 1, Nr. 2 oder Nr. 3 bei der Erteilung vorgelegen hat; sie ist zu widerrufen, wenn nachträglich Tatsachen eintreten, die die Versagung nach § 42 Abs. 2 Satz 3 Nr. 2 oder Nr. 3 rechtfertigen würden. In den Fällen des Satzes 1 kann auch das Ruhen der Genehmigung befristet angeordnet werden.

(2) Die zuständige Bundesoberbehörde kann die Genehmigung widerrufen, wenn die Gegebenheiten der klinischen Prüfung nicht mit den Angaben im Genehmigungsantrag übereinstimmen oder wenn Tatsachen Anlass zu Zweifeln an der Unbedenklichkeit oder der

wissenschaftlichen Grundlage der klinischen Prüfung geben. In diesem Fall kann auch das Ruhen der Genehmigung befristet angeordnet werden. Die zuständige Bundesoberbehörde unterrichtet unter Angabe der Gründe unverzüglich die anderen für die Überwachung zuständigen Behörden und Ethik-Kommissionen sowie die Kommission der Europäischen Gemeinschaften und die Europäische Agentur für die Beurteilung von Arzneimitteln.

(3) Vor einer Entscheidung nach den Absätzen 1 und 2 ist dem Sponsor Gelegenheit zur Stellungnahme innerhalb einer Frist von einer Woche zu geben. § 28 Abs. 2 Nr. 1 des Verwaltungsverfahrensgesetzes gilt entsprechend. Ordnet die zuständige Bundesoberbehörde die sofortige Unterbrechung der Prüfung an, so übermittelt sie diese Anordnung unverzüglich dem Sponsor. Widerspruch und Anfechtungsklage gegen den Widerruf, die Rücknahme oder die Anordnung des Ruhens der Genehmigung sowie gegen Anordnungen nach Absatz 5 haben keine aufschiebende Wirkung.

(4) Ist die Genehmigung einer klinischen Prüfung zurückgenommen oder widerrufen oder ruht sie, so darf die klinische Prüfung nicht fortgesetzt werden.

(5) Wenn der zuständigen Bundesoberbehörde im Rahmen ihrer Tätigkeit Tatsachen bekannt werden, die die Annahme rechtfertigen, dass der Sponsor, ein Prüfer oder ein anderer Beteiligter seine Verpflichtungen im Rahmen der ordnungsgemäßen Durchführung der klinischen Prüfung nicht mehr erfüllt, informiert die zuständige Bundesoberbehörde die betreffende Person unverzüglich und ordnet die von dieser Person durchzuführenden Abhilfemaßnahmen an; betrifft die Maßnahme nicht den Sponsor, so ist dieser von der Anordnung zu unterrichten. Maßnahmen der zuständigen Überwachungsbehörde gemäß § 69 bleiben davon unberührt.

APPENDIX VIII German GCP-Regulation (GCP-Verordnung)

Verordnung über die Anwendung der Guten Klinischen Praxis bei der Durchführung von klinischen Prüfungen mit Arzneimitteln zur Anwendung am Menschen

GCP-Verordnung

Vom 09. August 2004

geändert durch Artikel 1 der Verordnung vom 15.03.06 (BGBl. I, S. 542), zuletzt geändert durch Artikel 4 der Verordnung zur Ablösung der Betriebsverordnung für pharmazeutische Unternehmer vom 03.11.06 (BGBl. I, S. 2523)

Abschnitt 1

Allgemeine Vorschriften

§ 1

Zweck der Verordnung

(1) Zweck dieser Verordnung ist, die Einhaltung der Guten Klinischen Praxis bei der Planung, Durchführung und Dokumentation klinischer Prüfungen am Menschen und der Berichterstattung darüber sicherzustellen. Damit wird gewährleistet, dass die Rechte, die Sicherheit und das Wohlergehen der betroffenen Person geschützt werden und die Ergebnisse der klinischen Prüfung glaubwürdig sind.

(2) Bei klinischen Prüfungen mit Arzneimitteln, die aus einem gentechnisch veränderten Organismus oder einer Kombination von gentechnisch veränderten Organismen bestehen oder solche enthalten, bezweckt diese Verordnung darüber hinaus den Schutz der Gesundheit nicht betroffener Personen und der Umwelt in ihrem Wirkungsgefüge.

§ 2

Anwendungsbereich

Die Verordnung regelt die Aufgaben, Verantwortungsbereiche und Verfahren hinsichtlich der Planung, Genehmigung, Durchführung und Überwachung von klinischen Prüfungen am Menschen nach § 4 Abs. 23 des Arzneimittelgesetzes einschließlich Bioverfügbarkeits- und Bioäquivalenzstudien sowie hinsichtlich ihrer Dokumentation und der Berichterstattung über diese klinischen Prüfungen. Sie regelt außerdem den Schutz der Gesundheit nicht betroffener Personen und umweltbezogene Anforderungen bei klinischen Prüfungen mit Arzneimitteln, die aus einem gentechnisch veränderten Organismus oder einer Kombination von gentechnisch veränderten Organismen bestehen oder solche enthalten.

§ 3

Begriffsbestimmungen

(1) Multizentrische klinische Prüfung ist eine nach einem einzigen Prüfplan durchgeführte klinische Prüfung, die in mehr als einer Prüfstelle erfolgt und daher von mehr als einem Prüfer vorgenommen wird, wobei sich die weiteren Prüfstellen auch in anderen Mitgliedstaaten der Europäischen Union oder in Ländern befinden können, die nicht Mitgliedstaaten der Europäischen Union sind.

(2) Prüfplan ist die Beschreibung der Zielsetzung, Planung, Methodik, statistischen Erwägungen und Organisation einer klinischen Prüfung. Der Begriff schließt nachfolgende Fassungen und Änderungen des Prüfplans ein.

(2a) Betroffene Person ist ein Prüfungsteilnehmer oder eine Prüfungsteilnehmerin, die entweder als Empfänger des Prüfpräparates oder als Mitglied einer Kontrollgruppe an einer klinischen Prüfung teilnimmt.

(2b) Einwilligung nach Aufklärung ist die Entscheidung über die Teilnahme an einer klinischen Prüfung, die in Schriftform abgefasst, datiert und unterschrieben werden muss und nach ordnungsgemäßer Unterrichtung über Wesen, Bedeutung, Tragweite und Risiken der Prüfung und nach Erhalt einer entsprechenden

Dokumentation freiwillig von einer Person, die ihre Einwilligung geben kann oder aber, wenn die Person hierzu nicht in der Lage ist, von ihrem gesetzlichen Vertreter getroffen wird. Kann die betreffende Person nicht schreiben, so kann in Ausnahmefällen eine mündliche Einwilligung in Anwesenheit von mindestens einem Zeugen erteilt werden.

(2c) Ethik-Kommission ist ein unabhängiges Gremium aus im Gesundheitswesen und in nichtmedizinischen Bereichen tätigen Personen, dessen Aufgabe es ist, den Schutz der Rechte, die Sicherheit und das Wohlergehen von betroffenen Personen im Sinne des Absatzes 2a zu sichern und diesbezüglich Vertrauen der Öffentlichkeit zu schaffen, indem es unter anderem zu dem Prüfplan, der Eignung der Prüfer und der Angemessenheit der Einrichtungen sowie zu den Methoden, die zur Unterrichtung der betroffenen Personen und zur Erlangung ihrer Einwilligung nach Aufklärung benutzt werden und zu dem dabei verwendeten Informationsmaterial Stellung nimmt.

(3) Prüfpräparate sind Darreichungsformen von Wirkstoffen oder Placebos, die in einer klinischen Prüfung am Menschen getestet oder als Vergleichspräparate verwendet oder zum Erzeugen bestimmter Reaktionen am Menschen eingesetzt werden. Hierzu gehören Arzneimittel, die nicht zugelassen sind, und zugelassene Arzneimittel, wenn diese im Rahmen einer klinischen Prüfung am Menschen in einer anderen als der zugelassenen Darreichungsform oder für ein nicht zugelassenes Anwendungsgebiet oder zum Erhalt zusätzlicher Informationen über das zugelassene Arzneimittel eingesetzt werden.

(4) Prüferinformation ist die Zusammenstellung der für die klinische Prüfung am Menschen relevanten klinischen und nichtklinischen Daten über die in der klinischen Prüfung verwendeten Prüfpräparate.

(5) Inspektion ist die von der zuständigen Behörde oder Bundesoberbehörde durchgeführte Überprüfung von Räumlichkeiten, Ausrüstungen, Unterlagen, Aufzeichnungen, Qualitätssicherungssystemen und sonstigen nach Beurteilung der Behörde relevanten Ressourcen, die sich in der Prüfstelle, den Einrichtungen des Sponsors oder des Auftragsforschungsinstituts, den Laboratorien, den Herstellungsstätten von Prüfpräparaten oder in sonstigen Einrichtungen befinden. Sie dient dem Ziel, die Einhaltung der Regeln der Guten Klinischen Praxis (GCP), der Guten Herstellungspraxis (GMP) oder die Übereinstimmung mit den Angaben der Antragsunterlagen zu überprüfen.

(6) Unerwünschtes Ereignis ist jedes nachteilige Vorkommnis, das einer betroffenen Person widerfährt, der ein Prüfpräparat verabreicht wurde, und das nicht notwendigerweise in ursächlichem Zusammenhang mit dieser Behandlung steht.

(7) Nebenwirkung ist jede nachteilige und unbeabsichtigte Reaktion auf ein Prüfpräparat, unabhängig von dessen Dosierung.

(8) Schwerwiegendes unerwünschtes Ereignis oder schwerwiegende Nebenwirkung ist jedes unerwünschte Ereignis oder jede Nebenwirkung, das oder die tödlich oder lebensbedrohend ist, eine stationäre Behandlung oder deren Verlängerung erforderlich macht oder zu bleibender oder schwerwiegender Behinderung oder Invalidität führt oder eine kongenitale Anomalie oder einen Geburtsfehler zur Folge hat.

(9) Unerwartete Nebenwirkung ist eine Nebenwirkung, die nach Art oder Schweregrad nicht mit der vorliegenden Information über das Prüfpräparat übereinstimmt.

(10) Verblindung ist das bewusste Vorenthalten der Information über die Identität eines Prüfpräparates in Übereinstimmung mit den Angaben des Prüfplanes.

(11) Entblindung ist die Offenlegung der Identität eines verblindeten Prüfpräparates.

Abschnitt 2

Anforderungen an Prüfpräparate

§ 4

Herstellung und Einfuhr

(1) Die Herstellung, die Freigabe sowie die Einfuhr von Prüfpräparaten regelt die Arzneimittel- und Wirkstoffherstellungsverordnung vom 3. November 2006 (BGBl. I S. 2523) in der jeweils geltenden Fassung. Für die Kennzeichnung von Prüfpräparaten gilt § 5.

(2) Der Sponsor muss sicherstellen, dass die Herstellung und Prüfung des Prüfpräparates den Angaben des bei der zuständigen Bundesoberbehörde nach § 7 Abs. 4 Nr. 1 eingereichten Dossiers zum Prüfpräparat entspricht und die Herstellungsbetriebe und Prüflaboratorien für diese Tätigkeiten geeignet sowie zu deren Ausübung berechtigt sind. Bei Verwendung zugelassener Arzneimittel im Sinne des § 5 Abs. 8 gelten die Anforderungen als erfüllt, soweit der Sponsor bis auf die Kennzeichnung keine weiteren Herstellungsvorgänge unternimmt.

§ 5

Kennzeichnung von Prüfpräparaten

(1) Bei Prüfpräparaten muss die Kennzeichnung den Schutz der betroffenen Personen und die Rückverfolgbarkeit sicherstellen, die Identifizierung des Arzneimittels und der Prüfung ermöglichen und eine ordnungsgemäße Verwendung des Arzneimittels gewährleisten.

(2) Prüfpräparate dürfen außer in den Fällen nach den Absätzen 3 bis 5 oder in sonstigen begründeten Fällen nur in den Verkehr gebracht werden, wenn auf den Behältnissen und, soweit verwendet, auf den äußeren Umhüllungen in gut lesbarer Schrift, allgemein verständlich in deutscher Sprache und auf dauerhafte Weise angegeben sind:

1. Name oder Firma und Anschrift des Sponsors und die seines Auftragnehmers (CRO), soweit er nicht selbst Sponsor ist,
2. Telefonnummer des Sponsors und die seines Auftragnehmers (CRO), soweit er nicht selbst Sponsor ist, sofern die Telefonnummern nicht in einem Begleitdokument aufgeführt sind, das der betroffenen Person auszuhändigen ist,
3. Bezeichnung und Stärke des Prüfpräparates,
4. Chargenbezeichnung mit der Abkürzung "Ch.-B." oder Code-Nummer der Prüfung,
5. Darreichungsform,
6. Inhalt nach Gewicht, Volumen oder Stückzahl,
7. Art der Anwendung,
8. Dosierungsanleitung mit Einzel- oder Tagesgaben oder diesbezüglicher Verweis auf ein Begleitdokument oder die Anweisung eines Prüfarztes,
9. Dauer der Verwendbarkeit (Verfalldatum mit dem Hinweis "verwendbar bis" oder soweit die Art des Prüfpräparates dies erlaubt, Datum der Nachtestung) unter Angabe von Monat und Jahr,
10. Prüfplancode, der die Identifizierung der klinischen Prüfung, der Prüfstelle, des Prüfers und des Sponsors ermöglicht, sofern nicht in einem Begleitdokument enthalten, das der betroffenen Person ausgehändigt werden kann,
11. von der europäischen Datenbank vergebene EudraCT-Nummer, sofern diese nicht in einem Begleitdokument enthalten ist,
12. Identifizierungscode der betroffenen Person, und, sofern erforderlich, Kennzeichnung der Einnahmesequenz, sofern nicht in einem Begleitdokument enthalten, das der betroffenen Person ausgehändigt werden kann,
13. Hinweis, dass das Arzneimittel zur klinischen Prüfung bestimmt ist,
14. Aufbewahrungs- oder Lagerungshinweise, sofern dies in der Genehmigung für die klinische Prüfung vorgesehen ist,
15. Hinweis, dass das Prüfpräparat unzugänglich für Kinder aufbewahrt werden soll, sofern das Prüfpräparat dazu bestimmt ist, der betroffenen Person ausgehändigt zu werden,
16. besondere Vorsichtsmaßnahmen für die Beseitigung von nicht verwendeten Prüfpräparaten oder sonstige besondere Vorsichtsmaßnahmen, um Gefahren für die Gesundheit nicht betroffener Personen und die Umwelt zu vermeiden, oder Angaben für die Rückgabe.

Wenn Behältnis und äußere Umhüllung fest verbunden sind, ist die Kennzeichnung auf der äußeren Umhüllung ausreichend. Die Angabe nach Satz 1 Nr. 3 kann im Fall einer Verblindung der Prüfpräparate entfallen oder auf geeignete Weise verschlüsselt werden.

(3) Sofern Behältnis und äußere Umhüllung des Prüfpräparates dauernd zusammengehalten werden sollen und die äußere Umhüllung die unter Absatz 2 Satz 1 aufgeführten Angaben aufweist, muss das Behältnis mindestens die Angaben nach Absatz 2 Satz 1 Nr. 1, 3, 4, 5, 6, 7, 10 und 12 aufweisen, die Angabe nach Absatz 2 Satz 1 Nr. 7 kann bei festen oralen Darreichungsformen entfallen. Absatz 2 Satz 3 gilt entsprechend.

(4) Durchdrückpackungen sind mit den Angaben nach Absatz 2 Nr. 1, 3, 4, 7, 10 und 12 zu versehen, die Angabe nach Absatz 2 Satz 1 Nr. 7 kann bei festen oralen Darreichungsformen entfallen. Absatz 2 Satz 3 gilt entsprechend.

(5) Bei Behältnissen von nicht mehr als zehn Milliliter Volumen und bei Ampullen brauchen die Angaben nach Absatz 2 nur auf den äußeren Umhüllungen gemacht zu werden, jedoch müssen sich auf den Behältnissen und den Ampullen mindestens die Angaben nach Absatz 2 Satz 1 Nr. 1, 3, 4, 7, 10 und 12 befinden. Absatz 2 Satz 3 gilt entsprechend.

(6) Angaben nach Absatz 2, die zusätzlich in einer anderen Sprache wiedergegeben werden, müssen in beiden Sprachversionen inhaltsgleich sein. Weitere Angaben sind zulässig, sofern sie mit der Verwendung

des Prüfpräparates in Zusammenhang stehen, für die gesundheitliche Aufklärung wichtig sind und den Angaben nach Absatz 2 nicht widersprechen.

(7) Wenn die Dauer der Verwendbarkeit nachträglich verlängert werden soll, ist ein zusätzliches Etikett auf dem Behältnis und, soweit verwendet, auf der äußeren Umhüllung anzubringen, das das neue Verfalldatum oder das Datum der Nachtestung sowie die Chargenbezeichnung aufweist. Mit dem Etikett kann das frühere Datum, nicht aber die bereits vorhandene Chargenbezeichnung überdeckt werden.

(8) Bei Prüfpräparaten, die durch die zuständige Bundesoberbehörde zugelassene Arzneimittel sind oder für die die Kommission der Europäischen Gemeinschaften oder der Rat der Europäischen Union eine Genehmigung für das Inverkehrbringen gemäß Artikel 3 Abs. 1 oder 2 der Verordnung (EG) Nr. 726/2004 des Europäischen Parlaments und des Rates vom 31. März 2004 zur Festlegung von Gemeinschaftsverfahren für die Genehmigung und Überwachung von Human- und Tierarzneimitteln und zur Errichtung einer Europäischen Arzneimittel-Agentur (ABl. EU Nr. L 136 S. 1) erteilt hat, und die ohne zusätzliche Herstellungsmaßnahmen zur Verwendung in der klinischen Prüfung bestimmt sind, kann auf besondere Kennzeichnungen auf den Behältnissen und den äußeren Umhüllungen nach den Absätzen 2 bis 7 verzichtet werden, soweit es das Konzept der klinischen Prüfung erlaubt. Angaben nach Absatz 1 können auch in einem Begleitdokument aufgeführt werden.

§ 6

Entblindung in Notfallsituationen und Rücknahme

Bei verblindeten Prüfpräparaten muss der Sponsor ein Verfahren zur unverzüglichen Entblindung etablieren, das eine sofortige Identifizierung und, sofern erforderlich, eine unverzügliche Rücknahme der Prüfpräparate ermöglicht. Dabei ist sicherzustellen, dass die Identität eines verblindeten Prüfpräparates nur so weit offen gelegt wird, wie dies erforderlich ist.

Abschnitt 3

Genehmigung durch die Bundesoberbehörde und Bewertung durch die Ethik-Kommission

§ 7

Antragstellung

(1) Der Sponsor reicht in schriftlicher Form bei der für das zu testende Prüfpräparat zuständigen Bundesoberbehörde einen Antrag auf Genehmigung der klinischen Prüfung und bei der zuständigen Ethik-Kommission einen Antrag auf zustimmende Bewertung der klinischen Prüfung ein. Die dem Antrag beizufügenden Unterlagen können in deutscher oder in englischer Sprache abgefasst sein, soweit im Folgenden nichts anderes bestimmt ist. Antrag und Unterlagen sind zusätzlich auf einem elektronischen Datenträger einzureichen. Bei multizentrischen klinischen Prüfungen, die im Geltungsbereich des Arzneimittelgesetzes in mehr als einer Prüfstelle erfolgen, erhält jede weitere nach Landesrecht für einen Prüfer zuständige Ethik-Kommission (beteiligte Ethik-Kommission) zeitgleich eine Kopie des Antrags und der Unterlagen. Die nach § 42 Abs. 1 Satz 2 des Arzneimittelgesetzes zuständige Ethik-Kommission ist federführend für die Bearbeitung.

(2) Dem Antrag an die zuständige Ethik-Kommission und dem Antrag an die zuständige Bundesoberbehörde müssen vom Antragsteller die folgenden Angaben und Unterlagen beigefügt werden:

1. Kopie des Bestätigungsschreibens für die von der Europäischen Datenbank vergebene EudraCT-Nummer des Prüfplans,
2. vom Sponsor oder seinem Vertreter unterzeichnetes Begleitschreiben in deutscher Sprache, das die EudraCT-Nummer, den Prüfplancode des Sponsors und den Titel der klinischen Prüfung angibt, Besonderheiten der klinischen Prüfung hervorhebt und auf die Fundstellen der diesbezüglichen Informationen in den weiteren Unterlagen verweist,
3. vom Hauptprüfer oder vom Leiter der klinischen Prüfung sowie vom Sponsor oder seinem Vertreter unterzeichneter Prüfplan unter Angabe des vollständigen Titels und des Arbeitstitels der klinischen Prüfung, der EudraCT-Nummer, des Prüfplancodes des Sponsors, der Fassung und des Datums,
4. Name oder Firma und Anschrift des Sponsors und, sofern vorhanden, seines in der Europäischen Union oder in einem Vertragsstaat des Abkommens über den Europäischen Wirtschaftsraum niedergelassenen Vertreters,
5. Namen und Anschriften der Einrichtungen, die als Prüfstelle oder Prüflabor in die klinische Prüfung eingebunden sind, sowie der Hauptprüfer und des Leiters der klinischen Prüfung,
6. Angabe der Berufe von Prüfern, die nicht Arzt sind, der wissenschaftlichen Anforderungen des jeweiligen Berufs und der seine Ausübung voraussetzenden Erfahrungen in der Patientenbetreuung sowie Darlegung, dass der jeweilige Beruf für die Durchführung von Forschungen am Menschen

qualifiziert und Darlegung der besonderen Gegebenheiten der klinischen Prüfung, die die Prüfertätigkeit eines Angehörigen des jeweiligen Berufs rechtfertigen,
7. Prüferinformation,
8. Bezeichnung und Charakterisierung der Prüfpräparate und ihrer Wirkstoffe,
9. Gegenstand der klinischen Prüfung und ihre Ziele,
10. Anzahl, Alter und Geschlecht der betroffenen Personen,
11. Erläuterung der Kriterien für die Auswahl der betroffenen Personen sowie der hierzu zu Grunde gelegten statistischen Erwägungen,
12. Begründung dafür, dass die gewählte Geschlechterverteilung in der Gruppe der betroffenen Personen zur Feststellung möglicher geschlechtsspezifischer Unterschiede bei der Wirksamkeit oder Unbedenklichkeit des geprüften Arzneimittels angemessen ist,
13. Plan für eine Weiterbehandlung und medizinische Betreuung der betroffenen Personen nach dem Ende der klinischen Prüfung,
14. mit Gründen versehene Angaben ablehnender Bewertungen der zuständigen Ethik-Kommissionen anderer Mitgliedstaaten der Europäischen Union oder anderer Vertragsstaaten des Abkommens über den Europäischen Wirtschaftsraum sowie Versagungen beantragter Genehmigungen durch die zuständigen Behörden anderer Mitgliedstaaten der Europäischen Union oder anderer Vertragsstaaten des Abkommens über den Europäischen Wirtschaftsraum; sollten zustimmende Bewertungen einer Ethik-Kommission oder eine Genehmigung durch eine zuständige Behörde mit Auflagen versehen worden sein, sind diese anzugeben,
15. die Bestätigung, dass betroffene Personen über die Weitergabe ihrer pseudonymisierten Daten im Rahmen der Dokumentations- und Mitteilungspflichten nach § 12 und § 13 an die dort genannten Empfänger aufgeklärt werden; diese muss eine Erklärung enthalten, dass betroffene Personen, die der Weitergabe nicht zustimmen, nicht in die klinische Prüfung eingeschlossen werden.

(3) Der zuständigen Ethik-Kommission ist ferner vorzulegen:

1. Erläuterung der Bedeutung der klinischen Prüfung,
2. Bewertung und Abwägung der vorhersehbaren Risiken und Nachteile der klinischen Prüfung gegenüber dem erwarteten Nutzen für die betroffenen Personen und zukünftig erkrankte Personen,
3. Rechtfertigung für die Einbeziehung von Personen nach § 40 Abs. 4 und § 41 Abs. 2 und 3 des Arzneimittelgesetzes in die klinische Prüfung,
4. Erklärung zur Einbeziehung möglicherweise vom Sponsor oder Prüfer abhängiger Personen,
5. Angaben zur Finanzierung der klinischen Prüfung,
6. Lebensläufe oder andere geeignete Qualifikationsnachweise der Prüfer,
7. Angaben zu möglichen wirtschaftlichen und anderen Interessen der Prüfer im Zusammenhang mit den Prüfpräparaten,
8. Angaben zur Eignung der Prüfstelle, insbesondere zur Angemessenheit der dort vorhandenen Mittel und Einrichtungen sowie des zur Durchführung der klinischen Prüfung zur Verfügung stehenden Personals und zu Erfahrungen in der Durchführung ähnlicher klinischer Prüfungen,
9. Informationen und Unterlagen, die die betroffenen Personen erhalten, in deutscher Sprache sowie eine Darstellung des Verfahrens der Einwilligung nach Aufklärung,
10. Beschreibung der vorgesehenen Untersuchungsmethoden und eventuelle Abweichungen von den in der medizinischen Praxis üblichen Untersuchungen,
11. Beschreibung der vorgesehenen Verfahrensweise, mit der verhindert werden soll, dass betroffene Personen gleichzeitig an anderen klinischen Prüfungen oder Forschungsprojekten teilnehmen oder vor Ablauf einer erforderlichen Karenzzeit an der klinischen Prüfung teilnehmen,
12. Beschreibung, wie der Gesundheitszustand gesunder betroffener Personen dokumentiert werden soll,
13. Nachweis einer Versicherung nach § 40 Abs. 1 Satz 3 Nr. 8 und Abs. 3 des Arzneimittelgesetzes,
14. hinsichtlich der Vergütung der Prüfer und der Entschädigung der betroffenen Personen getroffene Vereinbarungen,
15. Erklärung zur Einhaltung des Datenschutzes,
16. alle wesentlichen Elemente der zwischen dem Sponsor und der Prüfstelle vorgesehenen Verträge,
17. Kriterien für das Aussetzen oder die vorzeitige Beendigung der klinischen Prüfung,
18. bei multizentrischen klinischen Prüfungen, die im Geltungsbereich des Arzneimittelgesetzes in mehr als einer Prüfstelle erfolgen, eine Liste der Bezeichnungen und Anschriften der beteiligten Ethik-Kommissionen,
19. eine Zusammenfassung der wesentlichen Inhalte des Prüfplans in deutscher Sprache, wenn der Prüfplan nach Absatz 2 Nr. 3 in englischer Sprache vorgelegt wird.

(4) Der zuständigen Bundesoberbehörde ist ferner vorzulegen:

1. das Dossier zum Prüfpräparat mit folgendem Inhalt:
   a) Unterlagen über Qualität und Herstellung,
   b) Unterlagen über die pharmakologisch-toxikologischen Prüfungen,
   c) vorgesehene Kennzeichnung,
   d) Herstellungserlaubnis,
   e) Einfuhrerlaubnis,
   f) Unterlagen über Ergebnisse von bisher durchgeführten klinischen Prüfungen sowie weitere bekannt gewordene klinische Erkenntnisse,
   g) zusammenfassende Nutzen-Risiko-Bewertung;
2. der Nachweis einer Versicherung nach § 40 Abs. 1 Satz 3 Nr. 8 und Abs. 3 des Arzneimittelgesetzes, wenn es sich bei dem Prüfpräparat um ein xenogenes Zelltherapeutikum handelt;
3. bei Prüfpräparaten, die aus einem gentechnisch veränderten Organismus oder einer Kombination von gentechnisch veränderten Organismen bestehen oder solche enthalten, gemäß Anhang II der Richtlinie 2001/18/EG des Europäischen Parlaments und des Rates vom 12. März 2001 über die absichtliche Freisetzung genetisch veränderter Organismen in die Umwelt und zur Aufhebung der Richtlinie 90/220/EWG des Rates (ABl. EG Nr. L 106 S. 1) eine Darlegung und Bewertung der Risiken für die Gesundheit nicht betroffener Personen und die Umwelt sowie eine Darlegung der vorgesehenen Vorkehrungen und gemäß Anhang III dieser Richtlinie Informationen über den gentechnisch veränderten Organismus, Informationen über die Bedingungen der klinischen Prüfung und über die den gentechnisch veränderten Organismus möglicherweise aufnehmende Umwelt, Informationen über die Wechselwirkungen zwischen dem gentechnisch veränderten Organismus und der Umwelt, ein Beobachtungsplan zur Ermittlung der Auswirkungen auf die Gesundheit nicht betroffener Personen und die Umwelt, eine Beschreibung der geplanten Überwachungsmaßnahmen und Angaben über entstehende Reststoffe und ihre Behandlung sowie über Notfallpläne. Der Sponsor kann insoweit auch auf Unterlagen Bezug nehmen, die ein Dritter in einem vorangegangenen Verfahren vorgelegt hat, sofern es sich nicht um vertrauliche Angaben handelt;
4. Bezeichnung und Anschrift der nach § 42 Abs. 1 Satz 1 und 2 des Arzneimittelgesetzes zuständigen Ethik-Kommission und Bezeichnung und Anschrift der zuständigen Behörden anderer Mitgliedstaaten der Europäischen Union und anderer Vertragsstaaten des Abkommens über den Europäischen Wirtschaftsraum, in denen die klinische Prüfung durchgeführt wird.

(5) Abweichend von Absatz 4 Nr. 1 kann anstelle des Dossiers die Zusammenfassung der Merkmale des Arzneimittels (SmPC) vorgelegt werden, wenn es sich bei dem Prüfpräparat um ein Arzneimittel handelt, das in einem Mitgliedstaat der Europäischen Union zugelassen ist oder für das die Kommission der Europäischen Gemeinschaften oder der Rat der Europäischen Union eine Genehmigung für das Inverkehrbringen gemäß Artikel 3 Abs. 1 oder 2 der Verordnung (EG) Nr. 726/2004 des Europäischen Parlaments und des Rates vom 31. März 2004 zur Festlegung von Gemeinschaftsverfahren für die Genehmigung und Überwachung von Human- und Tierarzneimitteln und zur Errichtung einer Europäischen Arzneimittel-Agentur (ABl. EU Nr. L 136 S. 1) erteilt hat und das in der klinischen Prüfung gemäß der Zusammenfassung der Merkmale des Arzneimittels (SmPC) angewendet werden soll. Soll das zugelassene Arzneimittel abweichend von der Zusammenfassung der Merkmale des Arzneimittels (SmPC) angewendet werden, und handelt es sich dabei ausschließlich um eine Abweichung von dem zugelassenen Anwendungsgebiet, so sind in der Regel keine zusätzlichen Daten zu Qualität, zu den Ergebnissen der pharmakologisch-toxikologischen Prüfung und zu klinischen Ergebnissen vorzulegen; bei anderen Abweichungen sind in Abhängigkeit von der Art der Abweichung zusätzliche Daten zu Qualität, zu den Ergebnissen der pharmakologisch-toxikologischen Prüfung und zu klinischen Ergebnissen nur dann vorzulegen, wenn die in der Zusammenfassung der Merkmale des Arzneimittels (SmPC) enthaltenen Angaben für die im Prüfplan vorgesehenen Anwendungsbedingungen nicht ausreichend sind. Wird das zugelassene Arzneimittel oder sein Wirkstoff von einem anderen als dem in der Zusammenfassung der Merkmale des Arzneimittels (SmPC) bezeichneten Hersteller oder nach einem anderen Verfahren hergestellt, so sind in Abhängigkeit von der Art der Änderungen zusätzliche Daten zur Qualität vorzulegen. Soweit erforderlich, sind darüber hinaus weitere Ergebnisse zu pharmakologisch-toxikologischen Prüfungen und zusätzliche klinische Ergebnisse vorzulegen. Wird das zugelassene Arzneimittel verblindet, so sind in Abhängigkeit von den durchgeführten Maßnahmen zur Verblindung zusätzliche Angaben zur Qualität vorzulegen.

(6) Ist das Prüfpräparat Gegenstand einer dem Sponsor durch die zuständige Bundesoberbehörde bereits genehmigten klinischen Prüfung, so kann der Sponsor auf die im Rahmen des vorhergehenden Genehmigungsverfahrens vorgelegten Unterlagen zum Prüfpräparat Bezug nehmen. Liegen dem Sponsor weitere Ergebnisse zu Qualität und Herstellung, zu pharmakologisch-toxikologischen Prüfungen oder

klinische Ergebnisse vor, die nicht Bestandteil der in Bezug genommenen Unterlagen zum Prüfpräparat sind, so sind diese vorzulegen.

(7) Sofern das Prüfpräparat ein Placebo ist, beschränkt sich der Inhalt des Dossiers zum Prüfpräparat auf die Angaben nach Absatz 4 Nr. 1 Buchstabe a.

§ 8

Bewertung durch die Ethik-Kommission

(1) Die nach § 42 Abs. 1 Satz 1 oder 2 des Arzneimittelgesetzes zuständige Ethik-Kommission bestätigt dem Sponsor innerhalb von zehn Tagen den Eingang des ordnungsgemäßen Antrags unter Angabe des Eingangsdatums oder fordert ihn auf, die von ihr benannten Formmängel innerhalb einer Frist von 14 Tagen zu beheben, wenn Unterlagen zum Antrag ohne Begründung hierfür fehlen oder der Antrag aus sonstigen Gründen nicht ordnungsgemäß ist.

(2) Innerhalb der nach § 42 Abs. 1 Satz 9 des Arzneimittelgesetzes geltenden Frist von höchstens 60 Tagen nach Eingang des ordnungsgemäßen Antrags übermittelt die zuständige Ethik-Kommission dem Sponsor und der zuständigen Bundesoberbehörde ihre mit Gründen versehene Bewertung. Während der Prüfung des Antrags auf zustimmende Bewertung kann die zuständige Ethik-Kommission nur ein einziges Mal zusätzliche Informationen vom Sponsor anfordern. Die Frist wird bis zum Eingang der zusätzlichen Informationen gehemmt. Die Hemmung beginnt mit Ablauf des Tages, an dem die Anforderung von der zuständigen Ethik-Kommission abgesendet wurde.

(3) Betrifft der Antrag eine klinische Prüfung, die im Geltungsbereich des Arzneimittelgesetzes nur in einer einzigen Prüfstelle durchgeführt wird, verkürzt sich die in Absatz 2 genannte Frist auf höchstens 30 Tage. Ist diese klinische Prüfung eine klinische Prüfung der Phase I, die als Teil eines mehrere klinische Prüfungen umfassenden Entwicklungsprogramms auf einer von dieser Ethik-Kommission zustimmend bewerteten klinischen Prüfung desselben Entwicklungsprogramms aufbaut, verkürzt sich die Frist auf 14 Tage. Diese Fristverkürzungen gelten nicht bei klinischen Prüfungen der in Absatz 4 genannten Arzneimittel.

(4) Bei klinischen Prüfungen von somatischen Zelltherapeutika und Arzneimitteln, die gentechnisch veränderte Organismen enthalten, verlängert sich die in Absatz 2 genannte Frist auf 90 Tage; eine weitere Verlängerung der Frist auf insgesamt 180 Tage tritt ein, wenn die zuständige Ethik-Kommission zur Vorbereitung ihrer Bewertung Sachverständige beizieht oder Gutachten anfordert. Für die klinische Prüfung von Gentransfer-Arzneimitteln beträgt die Frist höchstens 180 Tage. Für die Prüfung xenogener Zelltherapeutika gibt es keine zeitliche Begrenzung für den Bewertungszeitraum.

(5) Multizentrische klinische Prüfungen, die im Geltungsbereich des Arzneimittelgesetzes in mehr als einer Prüfstelle durchgeführt werden, bewertet die federführende Ethik-Kommission im Benehmen mit den beteiligten Ethik-Kommissionen. Die beteiligten Ethik-Kommissionen prüfen die Qualifikation der Prüfer und die Geeignetheit der Prüfstellen in ihrem Zuständigkeitsbereich. Ihre diesbezügliche Bewertung muss der federführenden Ethik-Kommission innerhalb von 30 Tagen nach Eingang des ordnungsgemäßen Antrags vorliegen.

§ 9

Genehmigung durch die zuständige Bundesoberbehörde

(1) Die zuständige Bundesoberbehörde bestätigt dem Sponsor innerhalb von zehn Tagen den Eingang des ordnungsgemäßen Antrags unter Angabe des Eingangsdatums oder fordert ihn auf, die von ihr benannten Formmängel innerhalb einer Frist von 14 Tagen zu beheben, wenn Unterlagen zum Antrag ohne Begründung hierfür fehlen oder der Antrag aus sonstigen Gründen nicht ordnungsgemäß ist.

(2) Die Prüfung des ordnungsgemäßen Antrags muss innerhalb der nach § 42 Abs. 2 des Arzneimittelgesetzes jeweils geltenden Frist abgeschlossen werden. Übermittelt die zuständige Bundesoberbehörde dem Sponsor mit Gründen versehene Einwände, kann dieser ein einziges Mal den Antrag innerhalb einer Frist von höchstens 90 Tagen nach Zugang entsprechend ändern. Nach Eingang der Änderung übermittelt die zuständige Bundesoberbehörde dem Sponsor innerhalb von 15 Tagen schriftlich die Genehmigung des Antrags oder, unter Angabe von Gründen, dessen endgültige Ablehnung. Die zuständige Ethik-Kommission erhält davon eine Kopie. Bei den in § 42 Abs. 2 Satz 7 Nr. 2 bis 4 des Arzneimittelgesetzes genannten Arzneimitteln beträgt die in Satz 3 genannte Frist 30 Tage. Für die Prüfung xenogener Zelltherapeutika gibt es keine zeitliche Begrenzung für den Genehmigungszeitraum.

(3) Betrifft der Antrag eine klinische Prüfung der Phase I, die als Teil eines mehrere klinische Prüfungen umfassenden Entwicklungsprogramms auf einer durch die zuständige Bundesoberbehörde genehmigten klinischen Prüfung desselben Entwicklungsprogramms aufbaut, verkürzt sich die jeweils geltende Frist auf

14 Tage, sofern dem Antrag die Angaben nach § 7 Abs. 4 Nr. 1 Buchstabe a und b des bereits genehmigten Antrags unverändert zu Grunde liegen. Diese Fristverkürzung gilt nicht bei klinischen Prüfungen der in Absatz 4 genannten Arzneimittel.

(4) Bei klinischen Prüfungen von Gentransfer-Arzneimitteln, somatischen Zelltherapeutika oder Arzneimitteln, die aus einem gentechnisch veränderten Organismus oder einer Kombination von gentechnisch veränderten Organismen bestehen oder solche enthalten, verlängert sich die Frist des § 42 Abs. 2 Satz 8 Halbsatz 1 des Arzneimittelgesetzes auf höchstens 90 Tage; eine weitere Verlängerung der Frist auf insgesamt höchstens 180 Tage tritt ein, wenn die zuständige Bundesoberbehörde zur Vorbereitung ihrer Entscheidung Sachverständige beizieht oder Gutachten anfordert. Für die Prüfung xenogener Zelltherapeutika gibt es keine zeitliche Begrenzung für den Genehmigungszeitraum. Bei Prüfpräparaten, die aus einem gentechnisch veränderten Organismus oder einer Kombination von gentechnisch veränderten Organismen bestehen oder solche enthalten, entscheidet die zuständige Bundesoberbehörde im Benehmen mit dem Bundesamt für gesundheitlichen Verbraucherschutz und Lebensmittelsicherheit; die Genehmigung der klinischen Prüfung durch die zuständige Bundesoberbehörde umfasst die Genehmigung der Freisetzung dieser gentechnisch veränderten Organismen im Rahmen der klinischen Prüfung.

(5) Zur Vorbereitung ihrer Entscheidung kann die zuständige Bundesoberbehörde die im Antrag nach § 42 Abs. 2 Satz 1 und 2 des Arzneimittelgesetzes enthaltenen oder nach § 10 Abs. 1 geänderten Angaben in der Prüfstelle, der Herstellungseinrichtung des Prüfpräparates, den an der Prüfung beteiligten Laboratorien, den Einrichtungen des Sponsors oder in sonstigen Einrichtungen überprüfen. Zu diesem Zweck können Beauftragte der zuständigen Bundesoberbehörde im Benehmen mit der zuständigen Behörde Betriebs- und Geschäftsräume zu den üblichen Geschäftszeiten betreten, Unterlagen einsehen und, soweit es sich nicht um personenbezogene Daten handelt, hieraus Abschriften oder Ablichtungen anfertigen sowie Auskünfte verlangen.

§ 10

Nachträgliche Änderungen

(1) Änderungen einer von der zuständigen Bundesoberbehörde genehmigten oder von der zuständigen Ethik-Kommission zustimmend bewerteten klinischen Prüfung, die geeignet sind,

1. sich auf die Sicherheit der betroffenen Personen auszuwirken,
2. die Auslegung der wissenschaftlichen Dokumente, auf die die Prüfung gestützt wird, oder die wissenschaftliche Aussagekraft der Studienergebnisse zu beeinflussen,
3. die Art der Leitung oder Durchführung der Studie wesentlich zu verändern,
4. die Qualität oder Unbedenklichkeit der Prüfpräparate zu beeinträchtigen oder
5. bei klinischen Prüfungen mit Arzneimitteln, die aus gentechnisch veränderten Organismen bestehen oder diese enthalten, die Risikobewertung für die Gesundheit nicht betroffener Personen und die Umwelt zu verändern,

darf der Sponsor nur vornehmen, wenn diese Änderungen von der zuständigen Ethik-Kommission zustimmend bewertet wurden, soweit sie die Angaben und Unterlagen nach § 7 Abs. 2 oder 3 betreffen, und wenn sie von der zuständigen Bundesoberbehörde genehmigt wurden, soweit sie die Angaben und Unterlagen nach § 7 Abs. 2 oder 4 betreffen. Die zustimmende Bewertung ist bei der zuständigen Ethik-Kommission, die Genehmigung ist bei der zuständigen Bundesoberbehörde zu beantragen. Der Antrag ist zu begründen.

(2) Die zuständige Ethik-Kommission hat eine Entscheidung über den ordnungsgemäßen Antrag auf zustimmende Bewertung der Änderungen innerhalb von 20 Tagen nach Eingang dem Sponsor und der zuständigen Bundesoberbehörde zu übermitteln. Bei multizentrischen klinischen Prüfungen, die im Geltungsbereich des Arzneimittelgesetzes in mehr als einer Prüfstelle durchgeführt werden, bewertet die federführende Ethik-Kommission die Änderungen im Benehmen mit den beteiligten Ethik-Kommissionen. Die beteiligten Ethik-Kommissionen prüfen die Qualifikation der Prüfer und die Geeignetheit der Prüfstellen in ihrem Zuständigkeitsbereich. Bei Arzneimitteln, die somatische Zelltherapeutika oder Gentransfer-Arzneimittel oder Arzneimittel sind, die gentechnisch veränderte Organismen enthalten oder deren Wirkstoff ein biologisches Produkt menschlichen oder tierischen Ursprungs ist oder biologische Bestandteile menschlichen oder tierischen Ursprungs enthält oder zu seiner Herstellung derartige Bestandteile erfordert, beträgt die Frist 35 Tage. Bei xenogenen Zelltherapeutika gibt es keine zeitliche Begrenzung für den Bewertungszeitraum.

(3) Übermittelt die zuständige Bundesoberbehörde dem Sponsor innerhalb von höchstens 20 Tagen nach Eingang des ordnungsgemäßen Änderungsantrags keine mit Gründen versehenen Einwände gegen die Änderungen, so gelten sie als genehmigt. Die zuständige Bundesoberbehörde kann den Sponsor auffordern,

die von ihm vorgeschlagenen Änderungen nach ihren Maßgaben abzuändern. Bei Arzneimitteln, die somatische Zelltherapeutika oder Gentransfer-Arzneimittel sind, die gentechnisch veränderte Organismen enthalten oder deren Wirkstoff ein biologisches Produkt menschlichen oder tierischen Ursprungs ist oder biologische Bestandteile menschlichen oder tierischen Ursprungs enthält oder zu seiner Herstellung derartige Bestandteile erfordert, beträgt die Frist 35 Tage. Bei xenogenen Zelltherapeutika gibt es keine zeitliche Begrenzung für den Genehmigungszeitraum.

(4) Zusätzliche Prüfstellen im Geltungsbereich des Arzneimittelgesetzes darf der Sponsor nur dann in die klinische Prüfung einbeziehen, wenn die zuständige Ethik-Kommission, die die klinische Prüfung zustimmend bewertet hat, die Einbeziehung der jeweiligen zusätzlichen Prüfstelle zustimmend bewertet. Dem Antrag auf Erteilung der zustimmenden Bewertung sind die auf die zusätzlichen Prüfstellen bezogenen Angaben nach § 7 Abs. 2 Nr. 5 und 8, Abs. 3 Nr. 4, 6 bis 8, 13, 14, 16 und 18 beizufügen. Jede Ethik-Kommission, die nach Landesrecht für einen Prüfer zuständig ist, der in einer zusätzlichen Prüfstelle für die Durchführung der klinischen Prüfung verantwortlich ist, erhält eine Kopie des ursprünglichen Antrags und der Unterlagen auf zustimmende Bewertung der klinischen Prüfung, der zustimmenden Bewertung der in Satz 1 genannten Ethik-Kommission und des Antrags auf zustimmende Bewertung der Einbeziehung der zusätzlichen Prüfstelle. Die federführende Ethik-Kommission setzt sich mit ihr ins Benehmen. Die zustimmende Bewertung gilt als erteilt, wenn die federführende Ethik-Kommission dem Sponsor nicht innerhalb von 30 Tagen nach Eingang des ordnungsgemäßen Antrags begründete Bedenken übermittelt. Die federführende Ethik-Kommission unterrichtet die zuständige Bundesoberbehörde über die Bewertung.

§ 11

Maßnahmen zum Schutz vor unmittelbarer Gefahr

(1) Unbeschadet des § 10 treffen der Sponsor und der Prüfer unverzüglich alle gebotenen Maßnahmen zum Schutz der betroffenen Personen vor unmittelbarer Gefahr, wenn neue Umstände die Sicherheit der betroffenen Personen beeinträchtigen können.

(2) Bei klinischen Prüfungen mit Arzneimitteln, die aus einem gentechnisch veränderten Organismus oder einer Kombination von gentechnisch veränderten Organismen bestehen oder solche enthalten, treffen der Sponsor und der Prüfer unbeschadet des § 10 alle gebotenen Maßnahmen zum Schutz der Gesundheit nicht betroffener Personen und der Umwelt.

Abschnitt 4

Dokumentations- und Mitteilungspflichten, Datenbanken, Inspektionen

§ 12

Anzeige-, Dokumentations- und Mitteilungspflichten des Prüfers

(1) Der Prüfer fügt seiner Anzeige nach § 67 des Arzneimittelgesetzes bei der zuständigen Behörde für jede von ihm durchgeführte klinische Prüfung die folgenden Angaben bei:

1. Name, Anschrift und Berufsbezeichnung des zur Anzeige verpflichteten Prüfers,
2. Bezeichnung der zuständigen Bundesoberbehörde sowie Datum der erteilten Genehmigung und, sofern zutreffend, Daten von Genehmigungen nachträglicher Änderungen nach § 10 Abs. 1,
3. Bezeichnung und Anschrift der nach § 42 Abs. 1 Satz 1 oder 2 des Arzneimittelgesetzes zuständigen Ethik-Kommission sowie Datum ihrer zustimmenden Bewertung und, sofern zutreffend, Daten von Genehmigungen nachträglicher Änderungen nach § 10 Abs. 1,
4. Bezeichnung und Anschrift der für den Prüfer und die Prüfstelle zuständigen beteiligten Ethik-Kommission sowie Datum ihrer diesbezüglichen Bewertung,
5. EudraCT-Nummer des Prüfplans,
6. Name oder Firma und Anschrift des Sponsors und, sofern zutreffend, seines in der Europäischen Union oder in einem Vertragsstaat des Abkommens über den Europäischen Wirtschaftsraum niedergelassenen Vertreters,
7. Name und Anschrift des Leiters der klinischen Prüfung sowie des Hauptprüfers,
8. Name und Anschrift der Prüflaboratorien und anderer Einrichtungen, die vom Prüfer eingebunden worden sind,
9. vollständiger Titel des Prüfplans einschließlich Prüfplancode und Zielsetzung,
10. zu prüfendes Anwendungsgebiet,
11. Art der klinischen Prüfung und ihrer Durchführung, einschließlich Angaben zu den besonderen Merkmalen betroffener Personen, auf die die besonderen Voraussetzungen nach § 41 des Arzneimittelgesetzes Anwendung finden,

12. geplanter Beginn und voraussichtliche Dauer,
13. Bezeichnung, Stärke, Darreichungsform, arzneilich wirksame Bestandteile und Art der Anwendung des Prüfpräparates,
14. Information, ob Regelungen des Betäubungsmittelrechts, des Gentechnikrechts oder des Strahlenschutzrechts zu beachten sind oder es sich um ein somatisches Gentherapeutikum oder Gendiagnostikum handelt,
15. Anzahl und Art der mitgeführten Vergleichspräparate.

(2) Der Prüfer unterrichtet die zuständige Behörde innerhalb von 90 Tagen über die Beendigung der klinischen Prüfung. Wurde die klinische Prüfung durch den Sponsor abgebrochen oder unterbrochen, erfolgt die Unterrichtung innerhalb von 15 Tagen unter Angabe der Gründe für den Abbruch oder die Unterbrechung.

(3) Der Prüfer kann dem Sponsor die Durchführung der Anzeige bei der zuständigen Behörde übertragen und hat dies zu dokumentieren.

(4) Der Prüfer hat den Sponsor unverzüglich über das Auftreten eines schwerwiegenden unerwünschten Ereignisses, ausgenommen Ereignisse, über die laut Prüfplan oder Prüferinformation nicht unverzüglich berichtet werden muss, zu unterrichten und ihm anschließend einen ausführlichen schriftlichen Bericht zu übermitteln. Personenbezogene Daten sind vor ihrer Übermittlung unter Verwendung des Identifizierungscodes der betroffenen Person zu pseudonymisieren.

(5) Über unerwünschte Ereignisse und unerwartete klinisch-diagnostische Befunde, die im Prüfplan für die Bewertung der klinischen Prüfung als entscheidend bezeichnet sind, unterrichtet der Prüfer den Sponsor innerhalb der im Prüfplan angegebenen Fristen. Absatz 1 Satz 2 gilt entsprechend.

(6) Im Fall des Todes einer betroffenen Person übermittelt der Prüfer der zuständigen Ethik-Kommission, bei multizentrischen Studien auch der beteiligten Ethik-Kommission, der zuständigen Bundesoberbehörde sowie dem Sponsor alle für die Erfüllung ihrer Aufgaben erforderlichen zusätzlichen Auskünfte. Personenbezogene Daten sind vor ihrer Übermittlung unter Verwendung des Identifizierungscodes der betroffenen Person zu pseudonymisieren.

(7) Bei klinischen Prüfungen mit Arzneimitteln, die aus einem gentechnisch veränderten Organismus oder einer Kombination von gentechnisch veränderten Organismen bestehen oder solche enthalten, hat der Prüfer den Sponsor unverzüglich über Beobachtungen von in der Risikobewertung nicht vorgesehenen etwaigen schädlichen Auswirkungen auf die Gesundheit nicht betroffener Personen und die Umwelt zu unterrichten.

§ 13

Dokumentations- und Mitteilungspflichten des Sponsors

(1) Der Sponsor hat alle ihm von den Prüfern mitgeteilten unerwünschten Ereignisse ausführlich zu dokumentieren. Diese Aufzeichnungen werden der zuständigen Bundesoberbehörde und den zuständigen Behörden anderer Mitgliedstaaten der Europäischen Union und anderer Vertragsstaaten des Abkommens über den Europäischen Wirtschaftsraum, in deren Hoheitsgebiet die klinische Prüfung durchgeführt wird, auf Anforderung übermittelt. Personenbezogene Daten sind vor ihrer Übermittlung unter Verwendung des Identifizierungscodes der betroffenen Person zu pseudonymisieren.

(2) Der Sponsor hat über jeden ihm bekannt gewordenen Verdachtsfall einer unerwarteten schwerwiegenden Nebenwirkung unverzüglich, spätestens aber innerhalb von 15 Tagen nach Bekanntwerden, die zuständige Ethik-Kommission, die zuständige Bundesoberbehörde und die zuständigen Behörden anderer Mitgliedstaaten der Europäischen Union und anderer Vertragsstaaten des Abkommens über den Europäischen Wirtschaftsraum, in deren Hoheitsgebiet die klinische Prüfung durchgeführt wird, sowie die an der klinischen Prüfung beteiligten Prüfer zu unterrichten. Personenbezogene Daten sind vor ihrer Übermittlung unter Verwendung des Identifizierungscodes der betroffenen Person zu pseudonymisieren.

(3) Der Sponsor hat bei jedem ihm bekannt gewordenen Verdachtsfall einer unerwarteten schwerwiegenden Nebenwirkung, die zu einem Todesfall geführt hat oder lebensbedrohlich ist, unverzüglich, spätestens aber innerhalb von sieben Tagen nach Bekanntwerden, der zuständigen Ethik-Kommission, der zuständigen Bundesoberbehörde und den zuständigen Behörden anderer Mitgliedstaaten der Europäischen Union und anderer Vertragsstaaten des Abkommens über den Europäischen Wirtschaftsraum, in deren Hoheitsgebiet die klinische Prüfung durchgeführt wird, sowie den an der Prüfung beteiligten Prüfern alle für die Bewertung wichtigen Informationen und innerhalb von höchstens acht weiteren Tagen die weiteren relevanten

Informationen zu übermitteln. Personenbezogene Daten sind vor ihrer Übermittlung unter Verwendung des Identifizierungscodes der betroffenen Person zu pseudonymisieren.

(4) Der Sponsor unterrichtet unverzüglich, spätestens aber innerhalb von 15 Tagen nach Bekanntwerden, die zuständige Bundesoberbehörde, die zuständige Ethik-Kommission und die zuständigen Behörden anderer Mitgliedstaaten der Europäischen Union und anderer Vertragsstaaten des Abkommens über den Europäischen Wirtschaftsraum, in deren Hoheitsgebiet die klinische Prüfung durchgeführt wird, über jeden Sachverhalt, der eine erneute Überprüfung der Nutzen-Risiko-Bewertung des Prüfpräparates erfordert. Hierzu gehören insbesondere

1. Einzelfallberichte von erwarteten schwerwiegenden Nebenwirkungen mit einem unerwarteten Ausgang,
2. eine Erhöhung der Häufigkeit erwarteter schwerwiegender Nebenwirkungen, die als klinisch relevant bewertet wird,
3. Verdachtsfälle schwerwiegender unerwarteter Nebenwirkungen, die sich ereigneten, nachdem die betroffene Person die klinische Prüfung bereits beendet hat,
4. Ereignisse im Zusammenhang mit der Studiendurchführung oder der Entwicklung des Prüfpräparates, die möglicherweise die Sicherheit der betroffenen Personen beeinträchtigen können.

(5) Sofern Maßnahmen nach § 11 ergriffen werden, unterrichtet der Sponsor unverzüglich die zuständige Bundesoberbehörde, die zuständige Behörde, die zuständige Ethik-Kommission und die zuständigen Behörden anderer Mitgliedstaaten der Europäischen Union und anderer Vertragsstaaten des Abkommens über den Europäischen Wirtschaftsraum, in deren Hoheitsgebiet die klinische Prüfung durchgeführt wird, über diese sowie die sie auslösenden Umstände.

(6) Der Sponsor hat der zuständigen Ethik-Kommission, der zuständigen Bundesoberbehörde und den zuständigen Behörden anderer Mitgliedstaaten der Europäischen Union und anderer Vertragsstaaten des Abkommens über den Europäischen Wirtschaftsraum, in deren Hoheitsgebiet die klinische Prüfung durchgeführt wird, während der Dauer der Prüfung einmal jährlich oder auf Verlangen eine Liste aller während der Prüfung aufgetretenen Verdachtsfälle schwerwiegender Nebenwirkungen sowie einen Bericht über die Sicherheit der betroffenen Personen vorzulegen.

(7) Erhält der Sponsor bei klinischen Prüfungen mit Arzneimitteln, die aus einem gentechnisch veränderten Organismus oder einer Kombination von gentechnisch veränderten Organismen bestehen oder solche enthalten, neue Informationen über Gefahren für die Gesundheit nicht betroffener Personen und die Umwelt, hat er diese der zuständigen Bundesoberbehörde unverzüglich mitzuteilen.

(8) Der Sponsor unterrichtet die zuständige Behörde, die zuständige Bundesoberbehörde, die zuständige Ethik-Kommission und die zuständigen Behörden anderer Mitgliedstaaten der Europäischen Union und anderer Vertragsstaaten des Abkommens über den Europäischen Wirtschaftsraum, in deren Hoheitsgebiet die klinische Prüfung durchgeführt wird, innerhalb von 90 Tagen über die Beendigung der klinischen Prüfung. Wurde die klinische Prüfung durch den Sponsor abgebrochen oder unterbrochen, erfolgt die Unterrichtung innerhalb von 15 Tagen unter Angabe der Gründe für den Abbruch oder die Unterbrechung.

(9) Der Sponsor übermittelt der zuständigen Bundesoberbehörde und der zuständigen Ethik-Kommission innerhalb eines Jahres nach Beendigung der klinischen Prüfung eine Zusammenfassung des Berichts über die klinische Prüfung, der alle wesentlichen Ergebnisse der klinischen Prüfung abdeckt.

(10) Der Sponsor stellt sicher, dass die wesentlichen Unterlagen der klinischen Prüfung einschließlich der Prüfbögen nach der Beendigung oder dem Abbruch der Prüfung mindestens zehn Jahre aufbewahrt werden. Andere Vorschriften zur Aufbewahrung von medizinischen Unterlagen bleiben unberührt.

§ 14

Mitteilungspflichten der zuständigen Bundesoberbehörde

(1) Die zuständige Bundesoberbehörde unterrichtet die für die Überwachung zuständigen Behörden, die zuständige Ethik-Kommission sowie die Kommission der Europäischen Gemeinschaften unverzüglich und unter Angabe von Gründen über die Anordnung von Abhilfemaßnahmen nach § 42a Abs. 5 des Arzneimittelgesetzes.

(2) Die zuständige Bundesoberbehörde übermittelt der für die Überwachung jeweils zuständigen Behörde auf Ersuchen alle hierfür benötigten Unterlagen.

(3) Die zuständige Bundesoberbehörde übermittelt Angaben an die bei der Europäischen Arzneimittel-Agentur eingerichtete Europäische Datenbank für klinische Prüfungen (EudraCT-Datenbank), insbesondere

1. Angaben zum Antrag auf Genehmigung der klinischen Prüfung durch die zuständige Bundesoberbehörde,
2. Angaben zum Antrag auf zustimmende Bewertung der klinischen Prüfung durch die zuständige Ethik-Kommission,
3. Änderungen des Antrags auf Genehmigung der klinischen Prüfung nach § 9 Abs. 2 Satz 2,
4. nachträgliche Änderungen nach § 10,
5. Beendigung der klinischen Prüfung,
6. Angaben über durchgeführte Inspektionen zur Überprüfung der Übereinstimmung mit der Guten Klinischen Praxis.

Personenbezogene Daten werden nicht übermittelt.

(4) Auf Anfrage der zuständigen Behörden anderer Mitgliedstaaten der Europäischen Union und anderer Vertragsstaaten des Abkommens über den Europäischen Wirtschaftsraum, der Europäischen Arzneimittel-Agentur oder der Europäischen Kommission übermittelt die zuständige Bundesoberbehörde die Informationen nach Absatz 3, die noch nicht in die EudraCT-Datenbank eingegeben wurden. Personenbezogene Daten werden nicht übermittelt.

(5) Die zuständige Bundesoberbehörde übermittelt Angaben zu allen Verdachtsfällen unerwarteter schwerwiegender Nebenwirkungen eines Prüfpräparates unverzüglich an die bei der Europäischen Arzneimittel-Agentur eingerichtete Eudravigilanz-Datenbank.

(6) Bei klinischen Prüfungen von Arzneimitteln, die aus einem gentechnisch veränderten Organismus oder einer Kombination von gentechnisch veränderten Organismen bestehen oder solche enthalten, unterrichtet die zuständige Bundesoberbehörde die Öffentlichkeit über den hinreichenden Verdacht einer Gefahr für die Gesundheit Dritter oder für die Umwelt in ihrem Wirkungsgefüge einschließlich der zu treffenden Vorsichtsmaßnahmen. Wird in den Fällen des Satzes 1 gemäß § 42a des Arzneimittelgesetzes die Genehmigung zurückgenommen oder widerrufen, das befristete Ruhen der Genehmigung oder eine Änderung der Bedingungen für die klinische Prüfung angeordnet und ist diese Maßnahme unanfechtbar geworden oder sofort vollziehbar, so soll die Öffentlichkeit auch hierüber unterrichtet werden. Die §§ 17a und 28a Abs. 2 Satz 2 und 3, Abs. 3 und 4 des Gentechnikgesetzes in der Fassung der Bekanntmachung vom 16. Dezember 1993 (BGBl. I S. 2066), das zuletzt durch Artikel 1 des Gesetzes vom 17. März 2006 (BGBl. I S. 534) geändert worden ist, gelten entsprechend.

§ 15

Inspektionen

(1) Inspektionen im Rahmen der Überwachung laufender oder bereits abgeschlossener klinischer Prüfungen werden nach § 64 Abs. 1 des Arzneimittelgesetzes durch die zuständige Behörde durchgeführt. Inspektionen zur Überprüfung der Übereinstimmung mit den Angaben aus den Unterlagen nach § 7 oder § 10 oder mit den Unterlagen nach § 22 Abs. 2 Nr. 3 des Arzneimittelgesetzes werden durch die zuständige Bundesoberbehörde durchgeführt.

(2) Die Inspektionen erfolgen im Namen der Europäischen Gemeinschaft. Ihre Ergebnisse werden von den anderen Mitgliedstaaten der Europäischen Union anerkannt. Die zuständige Behörde oder Bundesoberbehörde kann die zuständigen Behörden anderer Mitgliedstaaten um Unterstützung bei der Durchführung von Inspektionen bitten und ihrerseits bei von diesen Behörden initiierten Inspektionen Unterstützung leisten. Absatz 1 gilt entsprechend.

(3) Vorbehaltlich der zwischen der Europäischen Union und Drittländern getroffenen Vereinbarungen kann die zuständige Bundesoberbehörde einen mit Gründen versehenen Antrag an die Europäische Kommission richten, mit dem in einem Drittland um eine Inspektion der Prüfstelle, der Einrichtungen des Sponsors oder der Einrichtungen des Herstellers gebeten wird. Betrifft die Inspektion ein Arzneimittel, für das ein Antrag auf Zulassung bei der zuständigen Bundesoberbehörde gestellt wurde, kann diese die Inspektion in dem Drittland in eigener Zuständigkeit durchführen.

(4) Betrifft die Inspektion ein Arzneimittel, für das ein Antrag auf Zulassung nach den Verfahren der Verordnung (EG) Nr. 726/2004 des Europäischen Parlaments und des Rates vom 31. März 2004 zur Festlegung von Gemeinschaftsverfahren für die Genehmigung und Überwachung von Human- und Tierarzneimitteln und zur Errichtung einer Europäischen Arzneimittel-Agentur (ABl. EU Nr. L 136 S. 1) gestellt wurde, so unterliegt sie der Koordinierung durch die Europäische Arzneimittel-Agentur; sie wird durch die zuständige Bundesoberbehörde unter Berücksichtigung der von der Europäischen Arzneimittel-Agentur festgelegten Verfahren durchgeführt.

(5) Eine erneute Inspektion ist durchzuführen, wenn sie von der Europäischen Kommission auf Ersuchen eines anderen von der klinischen Prüfung betroffenen Mitgliedstaats oder der Europäischen Arzneimittel-Agentur aus dem Grund gefordert wird, dass sich bei der Überprüfung der Einhaltung der Guten Klinischen Praxis Unterschiede zwischen den einzelnen Mitgliedstaaten der Europäischen Union gezeigt haben.

(6) Die von der zuständigen Behörde durchgeführte Inspektion ist nach schriftlich festgelegtem Verfahren und nach einem im Voraus festgelegten Plan durchzuführen.

(7) Über das Ergebnis der Inspektion ist zeitnah ein Inspektionsbericht anzufertigen, in dem alle wesentlichen Feststellungen der Inspektion, insbesondere Mängel und Beanstandungen aufzunehmen sind. Im Fall einer Inspektion nach § 64 Abs. 1 des Arzneimittelgesetzes sind im Inspektionsbericht zusätzlich Anordnungen zur Abstellung der festgestellten Mängel und Beanstandungen aufzunehmen. Der von der zuständigen Behörde erstellte Inspektionsbericht wird der inspizierten Einrichtung und dem Sponsor mit der Aufforderung zur Abstellung der Mängel und Beanstandungen übermittelt, wobei der Schutz vertraulicher Angaben sicherzustellen ist. Die zuständige Behörde übermittelt der zuständigen Bundesoberbehörde die nach § 14 Abs. 3 Nr. 6 für die Übermittlung an die EudraCT-Datenbank notwendigen Angaben über durchgeführte Inspektionen. Ergibt die Inspektion, dass die Eignung des für die Durchführung der klinischen Prüfung in einer Prüfstelle verantwortlichen Prüfers oder die Eignung der Prüfstelle nicht gegeben sind, wird der Inspektionsbericht der für diesen Prüfer zuständigen Ethik-Kommission, bei multizentrischen klinischen Prüfungen auch der federführenden Ethik-Kommission, zur Verfügung gestellt. Auf begründetes Ersuchen wird der Inspektionsbericht auch der Europäischen Arzneimittel-Agentur und den zuständigen Behörden anderer Mitgliedstaaten der Europäischen Union zur Verfügung gestellt. Die Bewertung der vom Sponsor übermittelten Antwort zur Abstellung der Mängel und Beanstandungen obliegt der zuständigen Behörde nach einem festgelegten Verfahren.

(8) Bei Gefahr im Verzug ordnet die zuständige Behörde die sofortige Unterbrechung der Prüfung an und übermittelt diese Anordnung unverzüglich dem Sponsor und der zuständigen Bundesoberbehörde. Die zuständige Bundesoberbehörde prüft die Einleitung von Maßnahmen nach § 42a des Arzneimittelgesetzes und informiert die zuständigen Behörden über die von ihr ergriffenen Maßnahmen. Die zuständige Behörde kann, soweit erforderlich, weitere Maßnahmen nach § 69 des Arzneimittelgesetzes in eigener Zuständigkeit ergreifen.

(9) Die zuständige Behörde sowie die zuständige Bundesoberbehörde müssen über ein umfassend geplantes und korrekt geführtes Qualitätssicherungssystem verfügen, das zumindest die Organisationsstrukturen, Verantwortlichkeiten und Verfahren beinhaltet. Das Qualitätssicherungssystem ist vollständig zu dokumentieren und seine Funktionstüchtigkeit zu überwachen. Das für die Durchführung der Inspektionen verantwortliche Personal muss in ausreichender Zahl zur Verfügung stehen und für seine Aufgaben qualifiziert sowie unabhängig und frei von kommerziellen, finanziellen oder anderen Zwängen sein, die seine Entscheidung beeinflussen können. Den mit der Überwachung beauftragten Personen soll Gelegenheit gegeben werden, regelmäßig an fachlichen Fortbildungsveranstaltungen teilzunehmen. Die Qualifikation des Personals ist zu überprüfen.

Abschnitt 5

Übergangs- und Schlussbestimmungen

§ 16

Ordnungswidrigkeiten

Ordnungswidrig im Sinne des § 97 Abs. 2 Nr. 31 des Arzneimittelgesetzes handelt, wer vorsätzlich oder fahrlässig

1. entgegen § 12 Abs. 4 Satz 1 oder Abs. 7 oder § 13 Abs. 2 Satz 1 eine Unterrichtung nicht, nicht richtig, nicht vollständig oder nicht rechtzeitig vornimmt,
2. entgegen § 13 Abs. 3 Satz 1 eine Information nicht, nicht richtig, nicht vollständig oder nicht rechtzeitig übermittelt,
3. entgegen § 13 Abs. 6 eine Liste oder einen Bericht nicht, nicht richtig, nicht vollständig oder nicht rechtzeitig vorlegt oder
4. entgegen § 13 Abs. 7 eine Mitteilung nicht, nicht richtig, nicht vollständig oder nicht rechtzeitig macht.

§ 17

Übergangsbestimmungen

Für klinische Prüfungen von Arzneimitteln, für die vor dem 6. August 2004 die nach § 40 Abs. 1 Satz 2 des Arzneimittelgesetzes in der bis zum 6. August 2004 geltenden Fassung erforderlichen Unterlagen der für den Leiter der klinischen Prüfung zuständigen Ethik-Kommission vorgelegt worden sind, finden die Vorschriften dieser Verordnung keine Anwendung.

§ 18

Inkrafttreten

Die Verordnung tritt am zweiten Tage nach der Verkündung in Kraft.

APPENDIX IX Study Participant Card

PATIENTENAUSWEIS

Studie: GERP-ALS-2007

Bitte tragen Sie diesen Ausweis immer bei sich.

Herr/Frau.. nimmt seit .... an einer klinischen Studie mit dem Wirkstoff Pioglitazon teil. Untersucht wird der Einfluss von Pioglitazon im Vergleich zu einer Therapie mit Placebo bei Patienten mit Amyotropher Lateralsklerose unter Standardtherapie mit Riluzol. Die Behandlungsdauer beträgt 18 Monate. Die Studie ist doppelblind angelegt. Der Patient/die Patientin nimmt täglich 45 mg Pioglitazon oder Placebo.

Begleitmedikation:

Andere Präparate aus der gleichen Substanzklasse wie das Prüfmedikament dürfen während der Studie nicht eingenommen werden.

Bitte ändern Sie die medikamentöse Behandlung nicht ohne

Rücksprache mit dem Prüfarzt (Kontaktadresse siehe nächste Seite).

Bei einem Notfall, bei Erkrankungen oder bei Änderungen der Begleitmedikation informieren Sie bitte den nachfolgend genannten Prüfarzt.

Prüfarzt: (Name, Adresse, Tel.-Nr. angeben / Stempel)

Besuchstermine:

	Visite 1	Visite 2
Datum		
Uhrzeit		

	Visite 3	Visite 4
Datum		
Uhrzeit		

	Visite 5	Visite 6
Datum		
Uhrzeit		
